# Genome-wide RIP-Chip analysis of translational repressor-bound mRNAs in the *Plasmodium* gametocyte

**DOI:** 10.1186/s13059-014-0493-0

**Published:** 2014-11-03

**Authors:** Ana Guerreiro, Elena Deligianni, Jorge M Santos, Patricia AGC Silva, Christos Louis, Arnab Pain, Chris J Janse, Blandine Franke-Fayard, Celine K Carret, Inga Siden-Kiamos, Gunnar R Mair

**Affiliations:** Instituto de Medicina Molecular, Faculdade de Medicina da Universidade de Lisboa, Av. Prof. Egas Moniz, 1649-028 Lisbon, Portugal; Institute of Molecular Biology and Biotechnology (IMBB), Foundation of Research and Technology (FORTH), N. Plastira 100, Heraklio, Crete P.C. 71110 Greece; Pathogen Genomics Laboratory, Computational Bioscience Research Center, King Abdullah University of Science and Technology, Thuwal-Jeddah, Saudi Arabia; Department of Parasitology, Leiden University Medical Centre, Leiden, The Netherlands; Parasitology, Department of Infectious Diseases, University of Heidelberg Medical School, Im Neuenheimer Feld 324, 69120 Heidelberg, Germany

## Abstract

**Background:**

Following fertilization, the early proteomes of metazoans are defined by the translation of stored but repressed transcripts; further embryonic development relies on *de novo* transcription of the zygotic genome. During sexual development of *Plasmodium berghei*, a rodent model for human malaria species including *P. falciparum*, the stability of repressed mRNAs requires the translational repressors DOZI and CITH. When these repressors are absent, *Plasmodium* zygote development and transmission to the mosquito vector is halted, as hundreds of transcripts become destabilized. However, which mRNAs are direct targets of these RNA binding proteins, and thus subject to translational repression, is unknown.

**Results:**

We identify the maternal mRNA contribution to post-fertilization development of *P. berghei* using RNA immunoprecipitation and microarray analysis. We find that 731 mRNAs, approximately 50% of the transcriptome, are associated with DOZI and CITH, allowing zygote development to proceed in the absence of RNA polymerase II transcription. Using GFP-tagging, we validate the repression phenotype of selected genes and identify mRNAs relying on the 5′ untranslated region for translational control. Gene deletion reveals a novel protein located in the ookinete crystalloid with an essential function for sporozoite development.

**Conclusions:**

Our study details for the first time the *P. berghei* maternal repressome. This mRNA population provides the developing ookinete with coding potential for key molecules required for life-cycle progression, and that are likely to be critical for the transmission of the malaria parasite from the rodent and the human host to the mosquito vector.

**Electronic supplementary material:**

The online version of this article (doi:10.1186/s13059-014-0493-0) contains supplementary material, which is available to authorized users.

## Background

In multicellular eukaryotes (metazoans) early post-fertilization development of the newly formed zygote is orchestrated by proteins encoded by mRNAs provided by the egg [1]. Following fertilization, translation of stored, but previously translationally silent transcripts is instrumental in shaping the proteome of the early embryo when transcription is absent or low. Only after maternal-to-zygotic transition are mRNA templates for protein translation transcribed from the diploid, zygotic genome. Transcriptional activation relies on specific DNA binding factors like the *Drosophila* protein Zelda, a zinc finger DNA binding protein that recognizes promoters containing domains known as TAG team sites [2–4], while Nanog, Pou5f1, and SoxB1 activate zygotic gene expression in zebrafish [5].

Sexual development (gamete differentiation, fertilization, and ookinete formation) in the rodent malaria parasite *Plasmodium berghei* - a unicellular protozoan with a haploid life cycle and a model organism for human malaria parasites - coincides with transmission of the parasite from the mammalian host to the mosquito vector. Male and female sexual precursor cells (gametocytes) that develop inside the red blood cell apart from, but in parallel with asexually replicating forms, are taken up into the mosquito midgut during a blood meal where they rapidly differentiate into mature, free gametes. Fertilization of the immotile female by a flagellated male results in the formation of a round, diploid zygote that within 18 to 24 h transforms into a morphologically distinct cell type: the elongated and motile and unicellular ookinete. This specialized cell escapes the blood meal by penetrating the peritrophic membrane surrounding the blood meal, traverses the midgut epithelium and establishes the replicating oocyst that can give rise to thousands of sporozoites.

Gametogenesis and fertilization depend on kinase-mediated signaling events and surface proteins that ensure male–female recognition and fertilization, or function in flagellar motility of the male [6–8]; such proteins are already present in gametocytes. Transcriptome changes during gametogenesis are relatively small [9] and it is unknown whether any of the encoded proteins contribute to gamete maturation or fertilization. On the other hand, a large proportion of the female gametocyte (FG) transcriptome is assumed to be translationally repressed to provide mRNAs for zygote-to-ookinete transformation inside the mosquito midgut [10,11]. First identified for the ookinete surface protein P28 [12], a comparative transcriptome and proteome study suggested nine additional genes to be under translational control in gametocytes [13]. Storage of translationally quiescent *p28* in the FG requires the RNA binding proteins DOZI and CITH, and *p28* was shown to co-IP with both [10,11]. DOZI and CITH belong to the DDX6 helicase family and the LSM14 group, and although evolutionarily distant from many eukaryotes, are highly conserved proteins with homologs including Dhh1p and Scd6 from yeast, or Rck54 and Lsm14 from humans [10,11]. Distinct from P-bodies (cytoplasmic processing bodies which are involved in RNA degradation) or stress granules (stalled translation pre-initiation complexes), DOZI and CITH-defined mRNPs of *P. berghei* could not be shown to co-immunoprecipitate known RNA degradation factors such as the decapping enzyme, or eukaryotic translation initiation factors (eIF) 4G and eI4A, while both cap-binding eIF4E and poly(A) binding protein were [11]. The *Plasmodium* complex therefore most likely constitutes neither RNA degradation sites nor stress granules, but functions as an mRNA storage granule that prevents mRNA translation and allows long-term storage of this mRNA population while preventing RNA degradation. Consistent with such an interpretation, DOZI and CITH gene deletion mutants suffer a loss of hundreds of mRNAs [10,11]; one-quarter of those were found to be translated in the ookinete by SILAC [14]. But whether hundreds of maternal transcripts are indeed assembled into cytoplasmic mRNPs in the FG defined by DOZI and CITH is unknown, and merely suspected. The loss of mRNAs in null mutants could also be due to spurious downstream effects that are not caused by transcripts being direct targets for repression. Irrespective of the true nature of the effect, DOZI and CITH mutants develop into normal gametes that produce zygotes but are unable to develop into ookinetes [11]. This phenotype is entirely FG-dependent as DOZI::GFP and CITH::GFP protein expression is restricted to females [10,11]. Males lack the machinery to silence for example *p28* [15,16]; as a consequence, crossed with wild type females they produce viable ookinetes [11].

Here, using microarray profiling of RNA-immunoprecipitation (RIP) eluates we identify the global *P. berghei* gametocyte transcriptome that is stably associated with DOZI and CITH. Presenting the first RIP-Chip approach for *Plasmodium*, we demonstrate that zygote morphogenesis is largely transcription-independent and driven by maternal factors contained in DOZI/CITH mRNPs; our data reveal specific groups of maternal transcripts whose encoded proteins are responsible for the morphological and functional changes observed during zygote-to-ookinete transition. Using *in situ* tagging of five RIP-identified genes with GFP, we show that translational repression is dependent on 5′ untranslated regions (UTRs) rather than 3′ UTRs, and identify a novel crystalloid protein that plays an essential role for sporogony to occur.

## Results

### RNA immunoprecipitation reveals that DOZI and CITH associate with known translationally repressed transcripts

Among the more than 300 mRNAs that become de-stabilized in the absence of DOZI or CITH [10,11] are those encoding the well-characterized adhesins P25 and P28*,* and the ApiAP2 transcription factor AP2-o*.* All three mRNAs are transcribed but not translated in the FG [10,11,17]. In the absence of DOZI or CITH these transcripts become de-stabilized resulting in almost undetectable transcript expression. On a developmental level, DOZI and CITH knockout parasites produce zygotes that fail to develop further into the motile ookinete stage [10,11]. Transgenic parasites that express DOZI or CITH with a C-terminal Green-Fluorescent-Protein (GFP) tag behave like wild type forms; they produce normal numbers of motile ookinetes *in vitro* [10,11] and establish mosquito infections that transmit readily into the subsequent rodent host upon mosquito bite (unpublished observations). Clonal lines of these transgenic parasites were used in the present study and are referred to as DOZI::GFP and CITH::GFP. As an additional experimental control, we employed a reference transgenic line (GFPCON); it expresses soluble GFP in the cytoplasm of all parasite stages [18].

**Figure 1:**
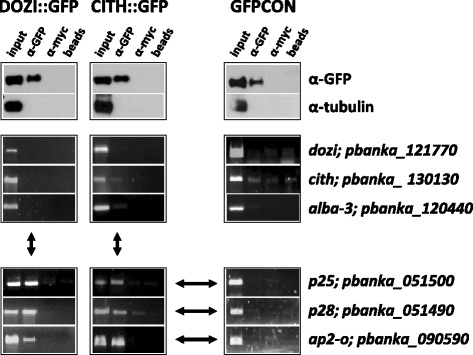
**DOZI and CITH-GFP tagged proteins bind known translationally repressed mRNA, but not translating transcripts.** Western blots of DOZI::GFP, CITH::GFP, and GFPCON (259.2 reference line that expresses cytoplasmic GFP throughout the life cycle) after IP of Nycodenz-purified gametocytes with anti-GFP (Roche); c-myc; or beads only (top panels). RT-PCR of co-eluted mRNAs (middle and lower panels). *p25*, *p28*, and *ap2-o* which are transcribed in the FG but translated only following fertilization elute with DOZI::GFP and CITH::GFP, while *cith* and *dozi* do not. Cytoplasmic GFP does not co-IP any of the shown mRNAs. Cartoons on the left side of the figure illustrate translation (middle panels) or translational silencing (lower panels).

To identify DOZI and CITH-associated mRNAs we prepared enriched gametocyte-stage fractions from all three parasite lines (DOZI::GFP, CITH::GFP, and GFPCON) by low-speed density gradient centrifugation and generated whole cell lysates using the detergent NP-40. Immunoprecipitation (IP) was performed with monoclonal anti-GFP antibodies in the presence of protease and RNase inhibitors. We have shown that DOZI and CITH are only required for the functionality of the FG [10,11] as is true for homologs in higher eukaryotes. Males in the absence of either protein will mate with wild type females and develop into healthy ookinetes. As such, it was not necessary to perform the IP on sorted males and sorted females. The specificity of each IP was determined by probing western blots for the GFP-tagged proteins and an alpha-tubulin control. Figure 1A shows that DOZI::GFP, CITH::GFP, and GFP exclusively IP with the anti-GFP antibody, while all control lanes are negative. RT-PCRs of co-eluted mRNAs using primers specific for the known translationally repressed genes *p25*, *p28*, and *ap2-o* show that these transcripts are clearly enriched in the anti-GFP fraction when compared to the two control IPs; these mRNAs associate almost exclusively with DOZI::GFP and CITH::GFP gametocytes, and rarely emerge from the control IPs performed with anti-myc antibodies or beads alone (Figure 1B).

*dozi*, *cith*, and *alba-3* which are known to be translated in the FG [6,10,11,19], are not present in the IP fraction suggesting that only translationally silenced mRNAs associate with DOZI and CITH in gametocytes, while translating ones are excluded from these mRNPs. In addition, the control IP using GFPCON parasite line was RT-PCR negative for translationally repressed as well as translating transcripts; the GFP-tag itself therefore does not enrich any of these mRNAs proving that only DOZI::GFP and CITH::GFP, but not GFP alone, bind known translationally silenced mRNAs.

### Global transcriptome of DOZI- and CITH-IPs

We next sought to identify the genome-wide extent of mRNAs bound by DOZI::GFP and CITH::GFP in blood stage gametocytes. To this end, total RNA generated from two independent DOZI::GFP and two independent CITH::GFP RIP experiments were processed for microarray profiling on a *P. berghei* Affymetrix custom designed tiling array (RMSANGER). We compared total RNA from input to anti-GFP-eluates in DOZI::GFP and CITH::GFP parasite lines.

**Figure 2:**
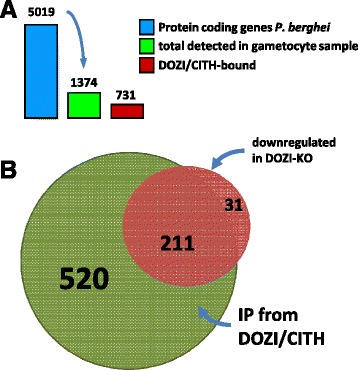
**Identification of DOZI/CITH-associated transcripts by microarray. (A)** Number of transcripts detected by microarray analyses. **(B)** Overlap of mRNAs previously shown to be downregulated in DOZI-depleted cells with the RIP-Chip transcriptome [10].

In total, 1,374 (27%) out of 5,028 *P. berghei* protein coding transcripts were detected on the array (75th percentile; [20] Version 11.1, 12 May 2014), a number consistent with previously reported data showing by microarray analysis that 24% of *P. falciparum* transcripts were found expressed in gametocytes [21]. Of these, 731 (53%) were bound to DOZI or CITH (Figure 2A). A total of 551 mRNAs were identified in the CITH::GFP IP eluate, 488 in the DOZI::GFP IP eluate; 308 were common to both while 243 were detected only in CITH and 180 only in DOZI. Out of 731, 98% of genes are conserved across all other malaria species (716 are found in *P. chabaudi chabaudi*, 716 in *P. falciparum* 3D7, 690 in *P. knowlesi* strain H, 705 in *P. vivax* Sal-1, and 720 *P. yoelii* 17X; Additional file 1: Table S1). We refer to the entire set of 731 mRNAs bound by DOZI and CITH as DOZI/CITH-associated (D/C-bound from here on) transcripts or the ‘repressome’.

Eighty-five percent of all D/C-bound transcripts were not detected as translated protein in the *P. berghei* FG proteome [6] (that is, 468/551 for CITH and 421/488 for DOZI) suggesting that, indeed, the majority of enriched mRNAs are not translated in the FG. When we included *P. falciparum* proteome data from early (I and II) and mature (IV and V) gametocyte stages [22,23], 80% of the 731 D/C-bound transcripts lacked protein evidence in this combined *P. berghei*-*P. falciparum* gametocyte proteome.

We had previously identified a loss of certain transcripts in *dozi* null mutants [10]. Originally encompassing 370 different transcripts in 2006, this number was revised to 242 by genome re-annotation and further sequencing efforts (plasmodb version 10.0); 87% (211) of those were found enriched by the RIP-Chip approach, 31 were not (Figure 2B and Additional file 1: Table S1), suggesting that, indeed, cells rely on DOZI to store and maintain this mRNA population.

### Validation of RIP-Chip data set by RT-PCR and GFP-tagging of novel, uncharacterized genes reveals 5′ UTR dependent repression and a novel crystalloid body protein

The data set of 731 genes associated with DOZI or CITH contained a number of transcripts characterized previously in the context of translational repression (Additional file 1: Table S1): these are mRNAs found to co-IP with DOZI [10], and genes whose untranslated regions had been verified experimentally to silence a GFP transgene in FG [16].

**Figure 3:**
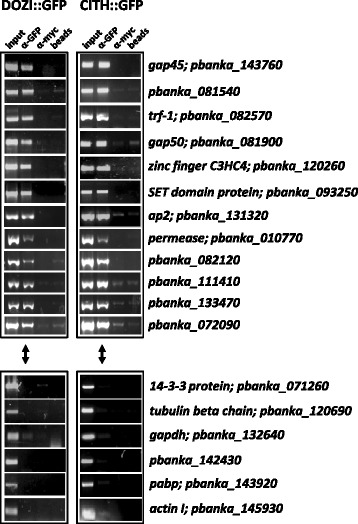
**Validation of DOZI and CITH-bound mRNAs.** Top panel are RIP-Chip-identified DOZI/CITH-bound mRNAs, while the lower panel shows mRNAs detected exclusively in the input fraction.

Here, we validated the RIP-Chip data in two independent IP experiments (DOZI::GFP and CITH::GFP) followed by detection of specific transcripts by RT-PCR. For this analysis we randomly selected 12 D/C-associated mRNAs, as well as six transcripts that were present in the input material but absent from the D/C-bound list. Every transcript, analyzed by RT-PCR, mirrored the microarray results, confirming the enrichment of certain mRNAs in the D/C-bound fraction while excluding others (Figure 3).

**Figure 4:**
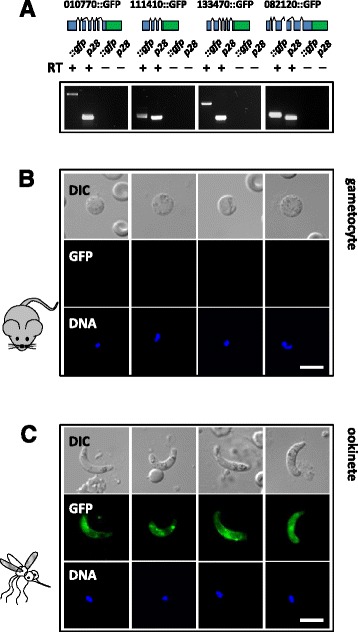
**Validation of translational repression by** ***in situ*** **GFP-tagging. (A)** Four genes were tagged C-terminally with GFP (top panels). These transgene transcripts are detected by RT-PCR in gametocytes. **(B)** GFP-tagged proteins cannot be detected in blood stage gametocytes, but is strong in ookinetes. **(C)** Parasite stages are shown by differential interference contrast (DIC), GFP immunofluorescence and Hoechst labeling of the nuclei. Scale bar = 5 μm.

From the above list, four uncharacterized protein coding genes from the D/C-bound list were selected for further experimental analysis of translational control by tagging each protein *in situ* with GFP at the C-terminus in order to monitor translation by fluorescence microscopy in gametocytes and ookinetes. These are PBANKA_082120, PBANKA_133470, PBANKA_111410 (conserved in *Plasmodium* but without a functional annotation), and PBANKA_010770 (a putative zinc/iron permease). In transgenic lines the GFP-fusion transcripts were readily detected in blood stage gametocytes by RT-PCR (Figure 4A) while GFP-fluorescence signal was absent (Figure 4B). Ookinetes on the other hand were strongly fluorescent (Figure 4C) confirming translational activation during post-fertilization development. Each of the four mRNAs were translationally repressed by virtue of their 5′ UTRs or elements within their open reading frames (ORF) as the 3′ UTR in all constructs was taken from the *P. berghei dhfr/ts* gene known to allow translation of *p28* in gametocytes [16]. Previously, only *p25* was shown to be repressed in a 5′ UTR-dependent manner [16]. The results here suggest that both mechanisms may frequently be employed.

**Figure 5:**
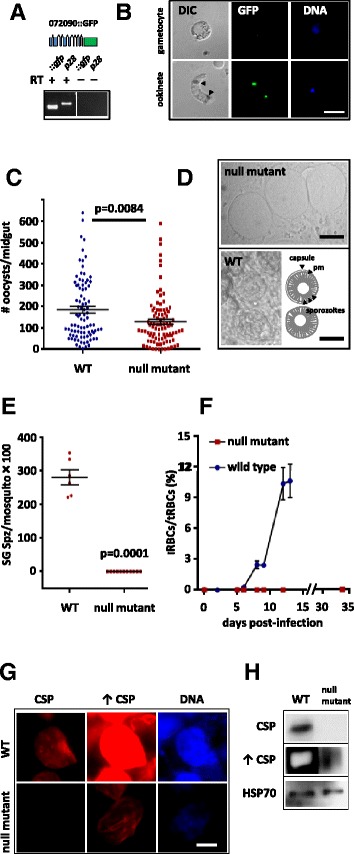
**PBANKA_072090 is translationally repressed in gametocytes, translated in ookinetes and an essential factor for sporozoite development. (A)** RT-PCR analysis shows correctly spliced, GFP-tagged mRNA in blood stage gametocytes. *p28* serves as control gene. **(B)** Live imaging of *pbanka_072090*::*gfp* parasites shows no expression in gametocytes while GFP (in green) is localized to discrete foci in ookinetes retrieved from a mosquito blood meal. Arrowheads indicate DIC-visible hemozoin clusters. Scale bar = 5 μm. **(C)** Null mutants parasites show reduced oocyst numbers on days 12 to 13 p.i. Wild type (WT; six independent experiments, *n = 88*; null mutant five independent experiments, *n = 92*). All values here (and below) are mean ± SEM; *P* values were obtained by Mann–Whitney test. **(D)** Mutant oocysts lack signs of sporulation and appear empty. On the contrary, WT oocysts have already formed sporozoites by day 16 p.i. (arrows). The plasma membrane (pm) of the oocyst is surrounded by a protective capsule. Scale bar = 30 μm. **(E)** Mutant parasites fail to develop sporozoite (Spz) and colonize salivary glands (SG). Absolute numbers of sporozoites per mosquito from five independent experiments are presented for both WT (*n = 6*) and null mutant (*n = 11*) parasites. **(F)** Mice bitten by mutant-infected mosquitoes do not develop blood stage infections (*n = 3*). iRBC = infected red blood cells; tRBC = total red blood cells **(G)** Immunofluorescence assay of oocyst-infected midguts at day 14 p.i. shows strongly reduced CSP expression in mutant parasites; DNA replication is also decreased as detected by Hoechst-33342 staining. ↑ CSP indicates extended exposure of same CSP image to the left. Scale bar = 20 μm. **(H)** Western blot analysis of oocyst-infected midguts from day 13 p.i. confirms reduced CSP expression in mutants. HSP70 serves as parasite loading control. ↑ CSP indicates extended exposure of same CSP blot above; when CSP can be detected in mutant oocysts, signal from WT oocyst is already saturated.

As a fifth gene, we GFP-tagged PBANKA_072090, a conserved *Plasmodium* protein with unknown function. Transcribed in gametocytes (Figure 5A), the fusion protein is only expressed in ookinetes (Figure 5B) and a target for repression via its 5′ UTR or perhaps its ORF. The tagged protein showed typical crystalloid localization (Figure 5B) known for members of the *Plasmodium* LCCL/LAP protein family (LCCL, Limulus factor C, Coch-5b2, and Lgl1) [24,25]. The crystalloid is a stage-specific organelle essential to sporozoite formation; established in the ookinete and often associated with malaria pigment, cells lacking the crystalloid or the crystalloid-resident protein PbSR fail to undergo sporogony (a process that allows the parasite to increase its population in the mosquito). In order to provide functional insights for this novel gene, we generated a gene deletion mutant by stable double crossover recombination event (Additional file 2: Figure S1). Although oocysts in the mutant were established in high numbers, they never produced sporozoites and remained empty throughout the course of the infection (Figure 5C and D). Consequently, the gene deletion mutant failed to colonise the salivary glands of the mosquito (Figure 5E), a prerequisite to transmit the infection to the subsequent rodent host. Mosquito bite-back experiments failed to cause infection (Figure 5F). The mutant parasite line suffered an absolute, 100% transmission defect. On a molecular level, mutant oocysts are characterized by a lack of DNA replication and expression of the sporozoite surface marker circumsporozoite protein (CSP) (Figure 5G and H).

In summary, GFP tagging of randomly selected D/C-bound transcripts confirm translational repression in gametocytes but protein expression in ookinetes and highlight a novel putative crystalloid protein. The number of proteins without a functional annotation (313/731; that is, 43%) represents a promising repertoire for identifying novel, developmentally regulated genes that enable *Plasmodium* sexual development and transmission to the mosquito vector.

### Translational and transcriptional contribution to ookinete formation

**Figure 6:**
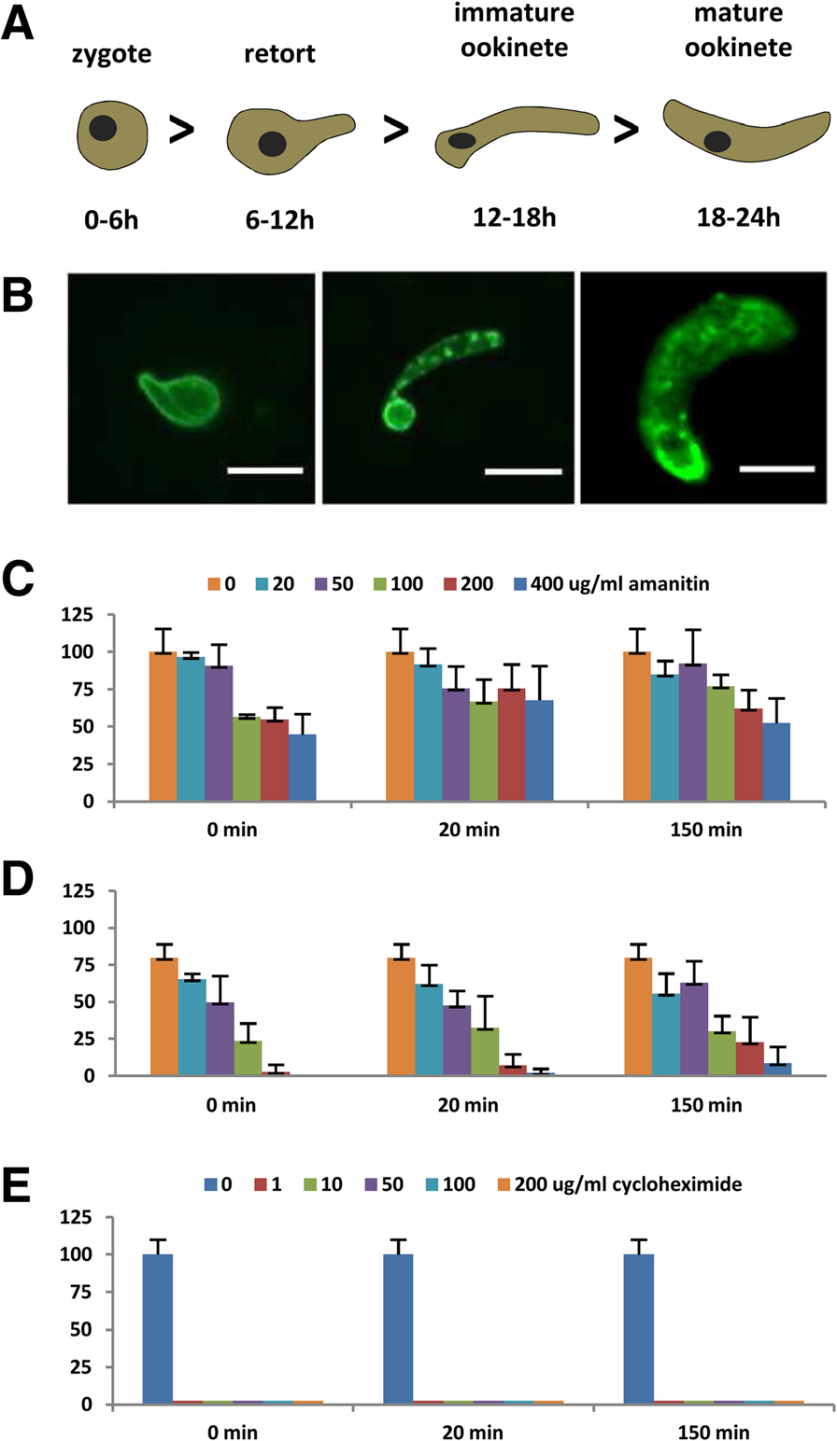
**Zygote to ookinete conversion occurs in the presence of transcription inhibitor - translation is essential. (A)** Schematic of zygote to ookinete transformation. **(B)** Representative images of developmental forms observed: retort (left and middle) or mature ookinete (right) cultured for 19 h with 400 mg/mL of α-amanitin (left and middle) or without (right) are labeled with anti-P28 and observed under a fluorescent microscope. Scale bar = 2.5 μm **(C)** Ookinete conversion rates (% of control) and **(D)** percentage mature ookinetes in the presence of α-amanitin. **(E)** Cycloheximide completely arrests post-fertilization development (% of control). Inhibitor concentrations are indicated on the figures as well as incubation times.

The large number of D/C-bound mRNAs raised the question whether zygote development could proceed with maternally supplied transcripts without the need for *de novo* synthesis of mRNAs. To test this, we added transcription and protein translation inhibitors to *in vitro* parasite cultures and determined zygote to ookinete development by quantifying ookinete conversion rates and scoring the development of ookinetes (Figure 6A and B).

Addition of α-amanitin - a well-characterized inhibitor of eukaryotic and *Plasmodium* RNA polymerase II (RNAP II) [26–29] - to a wild type *P. berghei* ookinete culture medium at three different time points after induction of gametogenesis resulted in a measurable but surprisingly modest decrease in conversion rates (that is, the percentage of parasite zygotes that develop into retort or mature ookinetes). At 20 and 50 μg.mL^-1^ ookinete conversion rates remained high and above 75% of the control at each time point; the lowest conversion rate (45% of control) was observed at the high concentration of 400 μg.mL^-1^ added at the very start of the experiment (Figure 6C). While conversion rates only slightly decreased as α-amanitin concentration increased, the percentage of fully developed ookinetes was above 60% at 20 and 50 μg.mL^-1^ of α-amanitin but dropped below 20% at 400 μg.mL^-1^ (Figure 6D). Our data indicate that *de novo* transcription - shown to be completely inhibited at 20 μg.mL^-1^ of α-amanitin in *P. falciparum* [29] - does not play a major role for post-fertilization development of the zygote *per se* but affects the potential of ookinetes to reach maturity. The inhibitory effect on ookinete maturation observed at elevated drug concentrations could be due to RNA polymerase III inhibition [30], which is responsible for the transcription of the 5S ribosomal RNA and transfer RNAs and could therefore affect translation.

The transformation of zygotes into ookinetes in the presence of α-amanitin was in stark contrast to results obtained from inhibition of protein translation with cycloheximide (a translational elongation inhibitor); these experiments resulted in a complete arrest of the transformation of zygotes into ookinetes at each tested concentration at every time point (Figure 6E). Protein synthesis is thus essential for ookinete development. In addition, among the most abundant transcripts detected in the gametocytes transcriptome but not associated with DOZI/CITH are those mRNAs encoding ribosomal proteins (81/92 of all detected ribosomal *Plasmodium* proteins, see Additional file 1: Table S1) supporting the notion that the molecular machinery for the translation of maternally supplied mRNAs is already provided by the female gametocyte. Combined, these data show that the initial morphological changes that occur during zygote to ookinete transformation do not rely on *de novo* transcription but depend on translation of mRNAs provided by the FG.

### Gene Ontology analysis of the gametocyte repressome

**Figure 7:**
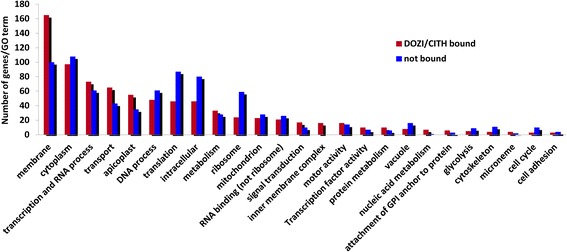
**Gene Ontology enrichment analysis of RIP-Chip identified mRNAs.**

To highlight groups of genes or pathways that play important roles during the 24-h developmental progression of the zygote into the motile ookinete, we performed a Gene Ontology (GO) analysis. Annotated GO terms were obtained from GeneDB [31] for *P. berghei* genes and if unavailable, annotations from syntenic orthologous *P. falciparum* genes were used [31]. In total, 150 out of 731 D/C-bound transcripts were not annotated with a GO term, leaving 581 D/C-enriched mRNAs to be categorized into Biological Process (BP), Cellular Component (CC), and Molecular Function (MF). The distribution of D/C-bound transcripts differed from the non-D/C-bound mRNAs detected in gametocytes for categories like membrane and inner membrane complex, microneme, attachment of GPI, transport, and more (Figure 7).

This was investigated in more detail using a hypergeometric test. The enriched gene pool was found to be significantly more abundant than expected by chance for the following GO terms: membrane, protein transport, isomerase activity, ribonucleoprotein complex and RNA processing, isomerase activity, and mitochondrion (Additional file 3: Table S2). In addition, manual gene set enrichment analysis revealed that adhesins, trafficking, proteins involved in gliding motility, transporters, chaperones, kinases, RNA binding and metabolism, splicing, microtubule, proteasome and the redox system were also significantly enriched (Additional file 4: Table S3).

Specifically, the enrichment analysis suggested a functional role for proteins involved in trafficking (hypergeometric test, *P* = 0.002 FDR corrected, Additional file 4: Table S3) like Sec61α, β and γ of the ER membrane protein translocator/translocon, Sec31 of the transitional (t) ER, Sec24a and b, SNAPs and SNAREs involved in vesicle trafficking, along with seven out of 10 Rabs. In addition, proteins functioning as adhesins like p25 and p28 that allow host-cell interactions [32], most members of the CPW-WPC multigene family of largely uncharacterized adhesins [33], ookinete microneme secreted proteins like CTRP (Circumsporozoite and thrombospondin-related protein) essential to ookinete motility [34–36], WARP (von Willebrand factor A-domain-related protein), SOAP (secreted ookinete adhesive protein), and several PSOPs (putative secreted ookinete proteins) reported to participate in midgut epithelium recognition [37–39], PPLP3 (*Plasmodium* perforin-like protein 3) involved in midgut invasion [40], and CelTOS (Cell-traversal protein for ookinetes and sporozoites) involved in cell traversal at both mosquito and liver stages [41] are enriched in the D/C-bound fraction (hypergeometric test, *P* = 0.029 FDR corrected, Additional file 4: Table S3). These findings are consistent with previous reports and expression studies that described surface adhesins and micronemal proteins as key molecules in midgut recognition and cell traversal of ookinetes [42,43].

As mentioned above, the crystalloid is a poorly understood organelle of *Plasmodium* parasites, established in the ookinete and lost in the oocyst. It contains proteins important for the development, infectivity and transmission of future sporozoites. LCCL (Limulus factor C, Coch-5b2, Lgl1) proteins localize to the crystalloid and three were recently reported to be translationally repressed in gametocytes [44]. All six members were detected in our analysis to be transcribed in the gametocyte, while only the three mRNAs experimentally found to be translationally repressed were also D/C-bound.

The mature ookinete is a motile cell and we found many genes of the gliding motility apparatus as well as genes involved in the formation of the inner membrane complex (IMC) (see for example [45,46] and Ginsburg, Hagai. ‘Malaria Parasite Metabolic Pathways’ [47]). Visual inspection and enrichment analyses of our data set revealed that the majority of proteins related to gliding motility are D/C-bound (Additional file 1: Table S1 and Additional file 4: Table S3). The gliding motility machinery relies on more than 40 proteins: some intimately (such as the actin-myosin motor as well as actin and actin binding proteins), others more peripherally associated (such as the proteins of the IMC and the subpellicular complex beneath the plasma membrane) (Additional file 5: Figure S2). Those associated with the IMC are membrane-anchored alveolins, GAPMs (glideosome associated proteins with multiple membrane spans), and SPMs (proteins associated with subpellicular microtubules); all of them belong to multigene families. We found a total of 40 mRNAs of ‘gliding motility-associated’ genes in the input fraction; of those, 26 were significantly enriched in the D/C-bound fractions (hypergeometric test, *P* = 0.001 FDR corrected, Additional file 4: Table S3). Out of these, seven encode proteins that have been genetically deleted or conditionally downregulated in *P. berghei* and resulted in ookinete developmental defects whereas formation of FG was normal (see Additional file 1: Table S1), thereby providing functional relevance to our findings. Transcripts encoding actin or actin-binding proteins however, were not found associated with DOZI or CITH in the RIP-Chip.

Finally, several proteases have been shown to play a role in invasive stages of *Plasmodium* and protease activity has been linked to the apical complex of these stages. Ookinetes have an apical complex that is associated with invasion and traversal of midgut epithelial cells. While specific proteases of the ookinete have not been yet identified, we found eight transcripts encoding proteases that were bound by D/C: five of the six known *P. berghei* plasmepsins (plasmepsins VI to X), including three reported to be expressed in exo-erythrocytic stages (PBANKA_040970, plasmepsin VI; PBANKA_051760, plasmepsin VII; and PBANKA_132910, plasmepsin VIII) and three of the eight rhomboids [48] (PBANKA_070270, ROM3; PBANKA_111780, ROM10; and PBANKA_110650, ROM4), suggesting a functional but yet to be defined role of these proteins in development.

## Discussion

Translational control by RNA binding proteins provides important regulatory mechanisms during *Plasmodium* parasite transmission - both from and to the mosquito vector [10,11,49,50]. Such mechanisms are key to maintaining a transmission-competent parasite population, and allowing rapid developmental progression in the next host. Fertilization and zygote formation occur within 30 min following a mosquito blood meal on an infected host; zygotes develop into mature ookinetes within 18 to 24 h after mating. By storing mRNAs separate from the translational machinery, the malaria parasite can quickly react to the sudden changes in environmental conditions, promoting survival in the hostile mosquito midgut while initiating an entirely novel developmental program. Storage of mRNAs for early sexual development is a key strategy in multicellular organisms to rapidly instigate post-fertilization development prior to maternal to zygote transition, when transcription from the diploid genome is still absent or low [4,51,52]. However, in multicellular organisms this is not sufficient for morphogenesis owing perhaps to the smaller number of regulated mRNAs. Maternal mRNPs defined by Car-1 and CGH-1 in *Caenorhabditis elegans* are required for cell division in the embryo [53] but account for less than 5% of the hermaphrodite gonad transcriptome [54]. In the malaria parasite *P. berghei* on the other hand DOZI and CITH associate with more than 50% of all detected transcripts. Therefore the generation of an entirely new life cycle form (unicellular) is possible with, but also critically depends on, mRNAs provided by the sexual precursor cell and requires little additional transcription of protein coding genes. Our result using α-amanitin in *in vitro* ookinete cultures strongly support this fact.

How translational repression is achieved in *P. berghei* is not understood. In yeast, an interaction of Scd6 (the yeast CITH homolog) through its C-terminal RGG domain with eIF4G blocks translation initiation [55]. Such domains are absent in CITH, but *P. berghei* and *P. falciparum* Alba-domain proteins contain up to 14 RGG domains and were found to co-IP with DOZI and CITH [11]; their precise function within the D/C-defined mRNPs is however still unknown. It is plausible that like in *Trypanosoma brucei* they are able to control translation; in addition to PABP and 4E, Alba-domain proteins were found to interact with the ribosomal protein P0 [56]. Although not found to share the same mRNP, trypanosome Scd6 and Dhh1 also regulate mRNA translation and stability [57–59]. *dhh1* is an essential gene in *T. brucei* bloodstream and insect forms [60], most likely due to this protozoan and related kinetoplastids relying largely on post-transcriptional gene regulatory mechanisms involving RNA regulons [61,62]. But as in *P. berghei* Dhh1 is clearly involved in the developmental regulation of genes during the transmission of the parasite between insect and mammalian host, which requires the transformation of the procyclic to the bloodstream form [58]; transcripts that encode functionally coupled proteins, for example those involved in oxidative phosphorylation, were identified to change identically following expression of a Dhh1 mutant.

The roles of DOZI and CITH as translational regulators therefore are evolutionarily ancient ones, but adapted to diverse organisms as well as developmental and environmental situations, and which is accomplished through the recruitment of additional protein factors that determine the ultimate fate of the regulated mRNAs. In *P. berghei* gametocytes DOZI and CITH bind half of all detected transcripts; whether mRNA binding occurs directly or indirectly is unknown, but very likely requires specific RNA binding proteins that will organize transcripts encoding functionally related proteins into RNA regulons [63,64]; translationally activated together they could orchestrate the formation of specific ookinete organelles such as the crystalloid or the gliding motility machinery. In yeast for example, each of the five proteins belonging to the Pumilio RNA binding family binds distinct mRNA populations that encode functionally related proteins [65]; they include, for example, mitochondrial (bound by Puf3) or nucleolar proteins (bound by Puf4). It was suggested in *Drosophila* that Puf-family Pumilio proteins could be responsible for the degradation of maternally supplied mRNAs following activation of the zygotic genome [52] while in *C. elegans* Car-1 binds PUF-5, -6, and -7 [66]. In *P. falciparum*, Pumilio2/Puf2 binds and confers silencing of *p25*, *p28*, and *plasmepsin iv* [67]. In *P. berghei* Puf2 was never found associated with DOZI or CITH in gametocytes, the reason why is unclear. Perhaps different mechanisms or timing of translational repression prior to maternal to zygotic transition exist between the rodent *P. berghei* and the human *P. falciparum* parasites. It is possible that the methodology we used does not permit the pull-down of Puf2. Alternatively, and an explanation we favor considering this being a fundamental developmental process, Puf2 and DOZI/CITH may not co-localize within the cell as D/C-defined mRNP foci are dynamic structures that may change composition from the initial assembly to the generation of a stable granule that is maintained over longer time periods. Mass-spectrometric analyses of factors bound to DOZI and CITH in *P. berghei* did identify several RNA binding proteins that could mediate direct targeting of certain transcripts for long-term storage [11]. One such factor is the *P. berghei* Homolog of Musashi identified in DOZI and CITH IP eluates; it contains several RNA recognition motifs but binds a much smaller mRNA population (unpublished observations). Nevertheless, factors like Homolog of Musashi could direct specific mRNAs to D/C-defined mRNPs in the gametocyte and recruit additional proteins while excluding others such as translation initiation factors. When, but also where, these binding events first occur is unknown. We have tried to identify a common motif that could be shared by all the transcripts identified here as enriched in the D/C-IP fractions to explain specific targeting to the mRNPs, but could not find any. To define the full repertoire of RNA binding proteins that are involved in translational regulation throughout the *Plasmodium* life cycle, global cross-linking and CLIP approaches will be needed; these have resulted in the identification of hundreds of RNA binding proteins in yeast [68] and mammalian cell lines [69–71] and defined certain cell types and responses to changes in environmental conditions.

Fully-formed ookinetes are banana-shaped, motile cells that are capable of traversing the mosquito midgut epithelium to establish an infection of the mosquito vector. The transformation of the zygote (round and immobile) into the ookinete requires largely unknown molecular processes that direct the rapid morphological remodeling, establish the necessary molecular machineries and processes underlying trafficking of proteins essential for motility (gliding motility apparatus), cell-cell adhesion (adhesins), cell traversal (perforins, proteases) as well as those decorating the surface with putative functions allowing recognition of the extracellular environment or immune evasion (like P25 and P28) [32]. It is striking that factors involved in all these steps but also key members of basic biological processes likely to quickly transduce developmental and differentiation signals, like transporters, kinases, Rabs, and other proteins involved in trafficking, are D/C-bound, further emphasizing the vital role of these molecules for successful zygote development and function in a different microenvironment.

Interestingly, 26 proteins of the repressome were already found to be important for ookinete development and function, including 19 for which gene deletion directly impaired ookinete development (please see [72] and Additional file 1: Table S1). These findings, along with our functional data, strongly support our view that the repressome is translated following gametocyte transmission to the mosquito vector and is key for ookinete morphogenesis.

When zygotic transcription is initiated after fertilization from the newly formed ookinete is unknown, and this timing varies from *Drosophila* to mouse [73]. The transcription factor AP2-o is translationally silenced in a DOZI/CITH-dependent manner. Once translated in the ookinete, AP2-o was found responsible for the transcriptional activation of only 15 genes [17]; they encode invasins such as Chitinase, SIAP (sporozoite invasion-associated protein 1), Cap380 (oocyst capsule protein), and PSOP2 and 7 (secreted ookinete proteins), that participate in ookinete and later mosquito stages infectious functions and therefore do not directly affect the formation of the ookinete itself. Of the eight AP2 DNA binding proteins identified in the gametocyte transcriptome, four were in the D/C-bound fraction including *ap2-o*. Together, all these AP2-proteins could be responsible for the transcriptional activation of a larger set of genes whose proteins define the mature ookinete.

## Conclusions

Our data provide the first comprehensive genome-wide analysis of *Plasmodium* DOZI and CITH-associated transcripts in blood stage gametocytes. Upon fertilization and zygote formation, development of the zygote into the ookinete proceeds through different stages in order to secure mosquito infection. Here we have shown that the translational repressors DOZI and CITH associate with over 700 *Plasmodium* conserved mRNAs, an increasing number of which are known to be translationally repressed. The D/C-bound mRNAs encode proteins involved in essential functions for the success of ookinete differentiation and function, which includes gliding motility, cell-cell adhesion, cell traversal, putative mosquito immune evasion as well as more fundamental processes like cellular trafficking. Our data further support that malaria parasites employ RNA binding protein-mediated translational control during life cycle progression that may involve RNA species-specific RNA binding proteins to regulate transcripts in a coordinated manner as RNA regulons. In particular, sexual development and transmission between hosts accompanied with large environmental changes can benefit from such strategies. We show that proteins translated from previously stored, maternal transcripts in the sexual precursor cells are the major contributors to early post-fertilization developmental success in this protozoan, exactly like in higher eukaryotes, where 35% to 75% of maternally supplied mRNAs ensure successful development of the zygote prior to maternal to zygotic transition [73]. The repressome reported here not only furthers our understanding of this complex biological process in malaria parasites but also highlights a large number of putative candidates with transmission blocking capacities.

## Material and methods

### Animal work

Animal work was performed in strict accordance with the recommendations of the Portuguese official Veterinary Directorate, which complies with the Portuguese Law (Portaria 1005/92). The Experiments on Animal Act strictly comply with the European Guideline 86/609/EEC and follow the FELASA (Federation of European Laboratory Animal Science Associations) guidelines and recommendations concerning laboratory animal welfare. All animal experiments were approved by the Portuguese official veterinary department for welfare licensing and the IMM Animal Ethics Committee. Animal experiments in LUMC (Leiden, The Netherlands) were approved by the Animal Experiments Committee of the Leiden University Medical Center (DEC 10099; 12042; 12120). All experiments were carried out using Swiss-OF1, Balb/c ByJ or C57Bl/6 female mice (aged 6 to 8 weeks, bred at Charles River, France or Harlan Laboratories, Inc.). All efforts were made to ensure minimal suffering to the animals.

### *Anopheles stephensi* mosquito maintenance

*A. stephensi* were bred at the insectary of the Instituto de Medicina Molecular (IMM). All life cycle associated experiments (mosquito infection and *in vivo* mouse infection) presented in this paper were performed as previously described [50].

### *P. berghei* ANKA lines

The following *P. berghei* ANKA parasite lines were used in the present work: line 683 cl1 (WT DOZI::GFP; RMgm-133) [10] expressing a C-terminally GFP-tagged version of *dozi* (PBANKA_121770); line 909 cl1 (WT CITH::GFP; RMgm-358) [11] expressing a C-terminally GFP-tagged version of *cith* (PBANKA_130130); line GFPCON [18]; line 820cl1m1cl1 [11]; and line cl15cy1 (WT, reference parent line of *P. berghei* ANKA) [74].

### Gametocyte enrichment, preparation of parasite lysates, and immunoprecipitation

Parasites were grown to a parasitemia of below 3% and collected by heart puncture. Gametocytes were enriched by Nycodenz gradient purification as described [10] and protein lysates prepared by NP-40 (0.5%) rupture on ice in 50 mM NaCl, 150 mM TrisHcl. Immunoprecipitation of DOZI::GFP, CITH::GFP, and GFP were performed with mouse monoclonal anti-GFP antibodies from Roche (catalogue # 11814460001); the control antibody was a mouse monoclonal anti-cmyc antibody (catalogue # 11667149001). Antibody-mRNP complexes were captured on protein G sepharose beads (GE Healthcare) and eluted with 2 × SDS-PAGE loading dye for protein and western analyses, or 1 mL TRIzol for RNA isolation. From the initial lysates one-quarter (50 μL) was kept as input sample, one-quarter was further processed for anti-GFP, one-quarter for anti-c-myc, and one-quarter for beads-only IPs. The nature of the RIP experiment results in minimal RNA co-elution in the control samples, which is why no loading control across the four samples is provided. The differences in output of known translationally repressed mRNAs (that is, *p25*, *p28*, and *ap2-0*) and known translated mRNAs (that is, *dozi*, *cith*, and *alba-3*) act as internal controls of the procedure during RT-PCR.

### Reverse transcriptase PCR of input and IP eluates

Total RNA from input and RIP samples was prepared with TRizol following the manufacturer’s recommendations and purified RNA was resuspended eluted into a final volume of 100 μL. The RNA concentration of the input sample was determined by Nanodrop™ spectrophotometry (Thermo Fisher Scientific, Waltham, MA, USA). Total input RNA was used for cDNA synthesis. Equivalent volumes were used for the three IP samples. After DNase-I treatment, cDNA synthesis was performed with a mixture of oligo d(T)s and random hexamers with SuperScript-II (Invitrogen). cDNA samples were diluted 1/20 and used in semi-quantitative PCR to detect the following transcripts: *p25*, *p28*, *ap2-o*, *dozi*, and *cith*; oligonucleotide primers used are shown in Additional file 6: Table S4. RT-PCR was performed using Fermentas Taq DNA polymerase as follows: an initial step of 95°C for 3 min, then 35 to 40 cycles of 95°C for 10 s, 43°C for 30 s, 62°C for 1 min, and a final step at 62°C for 10 min. Optimization of the RT-PCR experiments resulted in four washes being performed. As *p28* is highly expressed in gametocytes, to completely get rid of *p28* amplification in control lanes would require more washes but would also result in losing less abundant RNAs in the specific IPs. We chose to use the RT-PCR technique to clearly show that the anti-GFP IPs provides an enrichment of mRNAs as compared to the control samples.

An additional control consisted in performing IPs under identical conditions with lysates from the parasite reference line GFPCON [18]; this line expresses soluble GFP under the constitutive translation elongation factor 1α promoter. Total RNA, cDNA, and PCR (same primers) were performed as for the DOZI::GFP and CITH::GFP lines. For all PCRs, an RT-negative control was performed.

### RIP-Chip/microarray analyses

The entire amount of recovered RNA per IP was reverse transcribed using v/v random hexamers and T7 promoter primer poly(dT) and SupersciptII (both from Invitrogen) as recommended by the manufacturer. After generating the second strand, cDNA was amplified using the genomiphy kit (GE Healthcare). A total of 40 ng of total RNA was used as starting material to generate at least 6 μg of ds cDNA. Amplified cDNA was purified using G50 column (GE Healthcare). Three μg of amplified and purified cDNA was fractioned using DNaseI (Invitrogen) for 1 h at 37°C, then end-labeled using the Roche Terminal transferase kit and dCTP-Biotin. All samples (total RNA, amplified cDNA, and biotin labeled amplified cDNA) were quantitated using a Nanodrop™ (Thermo Fisher Scientific, Waltham, MA, USA) spectrophotometer. RNA quality and integrity were assessed on selected samples by electrophoresis.

Biotin-labeled, amplified cDNA (1.5 μg) from input and GFP-fractions was hybridized to the RMSANGER Affymetrix custom tiling array. A total of two independent replicates were used for each sample in this experiment. The chips were hybridized at 45°C for 18 h in the hybridization buffer provided by the manufacturer. After hybridization, the chips were washed, stained, and scanned according to Affymetrix recommendations. The CEL files were processed in R using the Bioconductor software suite [75]. Background correction was done using RMA [76] and data analyzed using the Limma package (R/Bioconductor) after remapping all the 3.2 million probes onto the newly annotated and updated *P. berghei* genome ([31], May 2014 release). Two contrasts of interest were computed and tested: CITH IP *vs*. CITH Input and DOZI IP *vs*. DOZI Input. Enrichment was considered when IP values were equal or greater than Input. FDR adjustment for multiple testing was performed using the method of Benjamini and Hochberg. Gene ontology (GO) terms enrichment analysis was performed as follows: GO IDs were extracted from the *P. berghei* ANKA annotation gff file (available in GeneDB) and the D/C-bound gene lists were tested against a filtered universal list of 2,133 *P. berghei* genes with annotated GO terms. Enriched GO terms (*P* value <0.05) were identified through conditional hypergeometric testing using GOstats R package [77].

### Data availability

The microarray data have been submitted to the ArrayExpress database [78] and were assigned the identifier E-MTAB-2900.

### Reverse transcriptase PCR of input and IP eluates; confirmation of microarray data

To confirm initial microarray data, additional IPs targeting DOZI::GFP and CITH::GFP were performed and cDNA prepared as described above. Genes identified in the microarray analyses were examined by semi-quantitative RT-PCR (see Figure 3) using primers listed in Additional file 6: Table S4.

### Bioinformatic analyses of RIP-Chip data

Transcriptome and proteome data were compared using R software. Data files were from [19,20] or the following publications: proteome of male/female *P. berghei* gametocytes [6]; mRNA loss in *dozi*- and *cith*- null mutants [10,11]; Hall 47mer RNA motif identified in putatively repressed transcripts [13]; translational repression transgene experiments [16]; *P. falciparum* gametocyte proteome data [79].

### Generation of GFP-tagged *P. berghei* ANKA parasites

*In situ* C-terminal GFP-tagging of PBANKA_010770, PBANKA_082120, PBANKA_111410, PBANKA_133470, and PBANKA_072090 was performed by single cross-over homologous recombination into the corresponding locus using constructs pLIS0097, pLIS0080, pLIS0085, pLIS0084, and pLIS0081, respectively. All constructs contain the *tgdhfr*/*ts* selectable marker under the control of *P. berghei dhfr/ts* 5′ and 3′ UTRs. Primers used to amplify the targeting regions of PBANKA_010770, PBANKA_082120, PBANKA_111410, PBANKA_133470, and PBANKA_072090, corresponding to the 3′ end of the ORF excluding the stop codon are listed in Additional file 6: Table S4. Targeting regions were cloned upstream and in frame with the GFP. Plasmids were linearized with AflII (pLIS0097), ClaI (pLIS0080), BsmI (pLIS0085), SnaBI (pLIS0084), or AflII (pLIS0081) and transfected into line cl15cy1 using published methods [80]. Transcription of *gfp* fusion genes was confirmed by RT-PCR using RNA from mixed blood stage forms of each mutant parasite line using the background WT line (cl15cy1) as negative control. Primers used for these RT-PCRs are listed in Additional file 6: Table S4.

### Generation of *P. berghei* PBANKA_072090 null mutants

To disrupt PBANKA_072090, we constructed the replacement construct pLIS0092 containing the pyrimethamine resistant *Toxoplasma gondii* (*tg*) *dhfr/ts* as a selectable marker cassette. See Additional file 2: Figure S1 and Additional file 6: Table S4 for details of the construct. Target sequences for homologous recombination were PCR-amplified from *P. berghei* WT genomic DNA using primers specific for the 5′ or 3′ flanking regions. The PCR-amplified target sequences were cloned upstream or downstream of the selectable marker to allow for integration of the linearized construct into the genomic locus by homologous recombination. DNA construct used for transfection was obtained after digestion of the replacement construct with the appropriate restriction enzymes. Transfection, selection, and cloning of mutant parasite lines were performed as described [81]. Correct deletion of the PBANKA_072090 gene was confirmed by diagnostic PCR and Southern analysis of FIGE-separated chromosomes (Additional file 2: Figure S1); chromosomes were hybridized with a probe recognizing the *tgdhfr*/*ts* selectable marker cassette. Absence of mRNA was determined by RT-PCR analysis (Additional file 2: Figure S1) using RNA collected from infected blood containing asexual blood stages and gametocytes. Two cloned lines were used for further phenotype analyses: 2099cl1m7 (PBANKA_072090 null mutant-a, used here) and 2100cl1m1 (PBANKA_072090 null mutant-b, data available upon request).

### Live imaging of gametocytes and ookinetes

Live imaging of gametocytes of the GFP-tagged parasite lines was performed after collecting iRBCs from infected mice, incubating with 1 μg/mL of Hoechst-33342/PBS and visualizing under a Leica DM5000B fluorescence microscope. Live imaging of blood meal-derived ookinetes was performed after collecting blood meals from fully engorged *A. stephensi* female mosquitoes at 16 h post infection, incubating with 1 μg/mL of Hoechst-33342/PBS and visualizing under a Leica DM5000B fluorescence microscope.

### Immunofluorescence assays (IFAs) of oocysts

To detect CSP expression in PBANKA_072090 null mutant oocysts, parasites at day 14 p.i. were stained with 3D11 mouse anti-PbCSP [82] (10 μg/mL) as primary antibody and goat anti-mouse IgG-Cy™3 (Jackson ImmunoResearch Laboratories, Inc., #115-166-003; 1:400) as secondary antibody. In these IFAs, samples were fixed with 4% PFA/PBS for 10 min at RT and simultaneously permeabilized and blocked for 1 h at RT with a mixture of 0.5% TritonX-100/PBS and 1% BSA/PBS. All antibody incubations were done in permeabilizing/blocking solution for 1 h at RT and 5 ug/mL of Hoechst-33342/PBS was used to stain nuclei. Images were taken with a Leica DM5000B or Zeiss Axiovert 200 M fluorescence microscope and processed using ImageJ 1.47n software (imagej.nih.gov/ij).

### Western analysis of CSP expression in PBANKA_072090 null mutant oocysts

To determine CSP expression, PBANKA_072090 null mutant-infected midguts were dissected at day 13 p.i. and resuspended in 1X Laemmli buffer. Samples were adjusted to 200 mM DTT, boiled and loaded onto SDS-PAGE gels. Nitrocellulose membranes were blocked for 1 h at RT with 5% skim milk/PBS-Tween 20 (0.05%), probed overnight at 4°C with 3D11 mouse anti-CSP [82], 0.17 μg/mL in blocking solution) or parasite-specific 2E6 mouse monoclonal anti-PbHSP70 [83], 7.5 μg/mL in blocking solution) as primary antibodies, and 1 h at RT with goat anti-mouse IgG-HRP (Santa Cruz Biotechnology, Inc.®, #sc-2005, 1:5,000-1:10,000 in PBS-Tween 20 (0.05%)) as secondary antibody. Westerns were developed with Immobilon™ Western Chemiluminescent HRP Substrate (Millipore, #P36599). Staining with the antibody recognizing *P. berghei* HSP70 was used as loading control.

### α-amanitin and cycloheximide treatments of ookinete culture

Infected blood of the *P. berghei* ANKA strain 234 was diluted in RPMI 1640 (pH = 8) and α-amanitin was added at different time points (0, 20, and 150 min from the beginning of exflagellation) at a final concentration of 20, 50, 100, 200, and 400 μg/mL. The zygote starts to form at 20 min post activation and is completed 150 min post activation.

To test the effect of cycloheximide on ookinete development, cultures were seeded as above and the drug was added at the same time intervals in final concentrations of 1, 10, 50, 100, and 200 μg/mL for cycloheximide.

To quantify ookinete conversion rates (the percentage of female gametes that converted to ookinetes), cultures were labeled with an antibody specific for the P28 surface protein expressed on the surface of macrogametes, zygotes and ookinetes. Briefly, following a 19 h-ookinete culture in presence of α-amanitin (400 μg/mL final concentration added at time 0) for retorts or without drug for mature ookinete forms, 5 μL were incubated with anti-P28 (1:100) and anti-mouse 488 (1:300, Invitrogen) antibodies in PBS. The cultures were incubated in the dark for 1 to 1.5 h at room temperature then placed on a coverslip and visualized using a Zeiss Axioskop 2 plus epifluorescent microscope.

### Oocyst production, sporozoite production, and transmission experiments

Oocyst and sporozoite production of the PBANKA_072090 null mutant parasites was analyzed by performing standard mosquito infections. Naïve female Balb/c ByJ mice were infected intraperitoneally (IP) with 10^6^ infected red blood cells (iRBCs) of each line. On days 4 to 5 post-infection (p.i.), these mice were anesthetized and *Anopheles stephensi* female mosquitoes allowed to feed for 30 min. Twenty-four hours after feeding, mosquitoes were anesthetized by cold shock and unfed mosquitoes were removed. Oocyst and sporozoite numbers were counted at days 11 to 13 and 20 to 22 after mosquito infection, respectively. Oocysts were counted after mercurochrome staining and measured using ImageJ 1.47n software (imagej.nih.gov/ij). Sporozoites were counted in pools of three to 24 mosquitoes. To test the infectivity of sporozoites, 10 infected mosquitoes were allowed to feed for 30 min on anesthetized naïve female Balb/c ByJ mice on days 20 to 21 p.i. Successful feeding was confirmed by the presence of blood in the abdomen of mosquitoes. Blood stage parasitemia in these mice were followed up to 33 days post-bite.

## Additional files

Additional file 1: Table S1. Lists the full RIP-ChIP data set. Additional file 2: Figure S1. Presents the strategy employed for the generation of PBANKA_072090 null mutants_a and _b. Only data pertaining to mutant _a are shown. Data for mutant _b are available upon request. Additional file 3: Table S2. Reports the GO terms enriched in *Plasmodium* genes found associated with DOZI and/or CITH. Additional file 4: Table S3. Reports the gene enrichment analysis of *P. berghei* genes found associated with DOZI and/or CITH. Additional file 5: Figure S2. Presents a schematic of the gliding motility apparatus in *Plasmodium* parasites and lists the D/C-bound transcripts involved in this process. Additional file 6: Table S4. List the primers used in GFP-tagged parasite lines and KO lines generation, as well as RT-PCRs.

## Abbreviations

CB: Crystalloid body
CITH: CAR-I and Trailer Hitch Homolog
DOZI: Development Of Zygote Inhibited
FG: Female gametocytes
mRNP: Messenger ribonucleoprotein
UTR: Untranslated regions
