# Supplementary material for: Genome-wide RIP-Chip analysis of translational repressor-bound mRNAs in the Plasmodium gametocyte
Source: Genome Biol. 2014 Nov 3;15(11):493. doi: 10.1186/s13059-014-0493-0 (PMC4234863; doi:10.1186/s13059-014-0493-0)
Supplement: Additional file 1: Table S1. — Lists the full RIP-ChIP data set. [file 13059_2014_493_MOESM1_ESM.pdf]

Table S1: Full RIP-ChIP dataset. PB ID: *P. berghei* gene names; Product Description: *P. berghei* product description; FC DOZI/WT: fold change expression from the DOZI-KO vs. wild type [Mair et al., 2006]; Confidence: corresponds to the false discovery rate (expected percent of false predictions in the set of predictions (see www.plasmodb.org); PF ID: *P. falciparum* gene names; PF Product Description: *P. falciparum* product description; Manual Annotation: obtained from literature scan, BLASTs etc; CITH: CITH immunoprecipitation, 0, not enriched, 1, enriched; DOZI: DOZI immunoprecipitation, 0, not enriched, 1, enriched; DOZI/CITH: 0, not enriched, 1, enriched in one IP, 2, enriched in both IPs; Gene Deletion Phenotype (RMgmDB, www.pberghei.eu); PB and PF annotated Gene Ontology (GO) Molecular Function, Biological Process and Cellular Component.

		FC								Gene Deletion Phenotype	*P. berghei* annotated GO			syntenic *P. falciparum* orthologs annotated GO		
PBANKA	Product Description	DOZI/WT	Confidence	PF ID	PF Product Description	Manual Annotation	CITH	DOZI	DOZI/CIT	in mosquito and liver stages	Molecular Function	Biological Process	Cellular Component	Molecular Function	Biological Process	Cellular Component
PBANKA_134780	20 kDa chaperonin, putative (CPN20)	-2.73	0.75	PF3D7_1333000	20 kDa chaperonin (CPN20)	chaperone	1	1	2	na	ATP binding, chaperone binding	protein folding	apicoplast			
PBANKA_071420	ClpB protein, putative (ClpB1)	-1.63	0.7	PF3D7_0816600	ClpB protein, putative (ClpB1)	chaperone	1	1	2	na	ATP binding, nucleoside-triphosphatase activity, protein binding	protein metabolic process	apicoplast			
PBANKA_051500	25 kDa ookinete surface antigen precursor (P25)	-5.59	0.75	PF3D7_1031000	25 kDa ookinete surface antigen precursor (Pfs25)	adhesin	1	1	2	different in fertilization, ookinete and oocyst	null	null	cell surface, membrane			
PBANKA_051490	28 kDa ookinete surface protein (P28)	-2.62	0.75	PF3D7_1030900	28 kDa ookinete surface protein (Pfs28)	adhesin	1	1	2	different in fertilization, ookinete and oocyst	null	null	cell surface, membrane			
PBANKA_051170	topoisomerase, putative			PF3D7_1027600	topoisomerase, putative	DNA replication	1	1	2	na	ATP binding, DNA binding, catalytic activity	DNA metabolic process	chromosome			
PBANKA_081680	glycolipid transfer protein, putative	-5.27	0.75	PF3D7_0915800	glycolipid transfer protein, putative	lipid metabolism	1	1	2	na	glycolipid binding, glycolipid transporter activity	glycolipid transport	cytoplasm			
PBANKA_102870	conserved Plasmodium protein, unknown function			PF3D7_1414000	conserved protein, unknown function	unknown function	1	1	2	na	null	null	cytoplasm, nucleus			
PBANKA_071290	high mobility group protein, putative (HMGB2)	-3.31	0.71	PF3D7_0817900	high mobility group protein (HMGB2)	chromatin	1	1	2	na	DNA binding	null	cytoplasm, nucleus			
PBANKA_092000	multiprotein bridging factor type 1, putative			PF3D7_1128200	multiprotein bridging factor type 1, putative	transcription	1	1	2	na	sequence-specific DNA binding	null	cytosol, nuclear periphery			
PBANKA_103840	selenoprotein, putative (Sel1)			PF3D7_1403600	selenoprotein (Sel1)	redoxmeth	1	1	2	na	null	null	endoplasmic reticulum			
PBANKA_060930	GPI mannosyltransferase I, putative			PF3D7_1210900	GPI mannosyltransferase I (PIG-M)	GPI	1	1	2	na	transferase activity, transferring hexosyl groups	GPI anchor biosynthetic process	endoplasmic reticulum membrane, integral to membrane			
PBANKA_083680	Pb-fam-1 protein			PF3D7_1147800.1/2	merozoite adhesive erythrocytic binding protein (MAEBL)	contaminant	1	1	2	na	null	null	host cell membrane			
PBANKA_133890	glideosome associated protein with multiple membrane spans 1, putative	-4.14	0.75	PF3D7_1323700	glideosome associated protein with multiple membrane spans 1 (GAPM1)	gliding motility	1	1	2	KO not successful	null	null	inner membrane complex			
PBANKA_090710	inner membrane complex protein 1b (IMC1b)	-3.1	0.73	PF3D7_1141900	inner membrane complex protein 1b, putative (IMC1b)	gliding motility	1	1	2	different in fertilization, ookinete and oocyst	null	null	inner membrane complex			
PBANKA_124060	inner membrane complex protein 1g, putative (IMC1G)	-4.13	0.75	PF3D7_0525800	membrane skeletal protein IMC1-related	gliding motility	1	1	2	na	null	null	inner membrane complex			
PBANKA_143660	inner membrane complex protein 1h (IMC1h)	-2.99	0.73	PF3D7_1221400	membrane skeletal protein, putative (ALV3)	gliding motility	1	1	2	different in fertilization, ookinete, oocyst, sporozoite and liver stage	null	null	inner membrane complex			
PBANKA_130290	copper transporter, putative			PF3D7_1439000	copper transporter putative	transporter	1	1	2	na	copper ion transmembrane transporter activity	copper ion transport	integral to membrane			
PBANKA_123130	metabolite/drug transporter, putative	-3.99	0.75	PF3D7_0516500	metabolite/drug transporter, putative	transporter	1	1	2	not different from wild type	tetracycline:hydrogen antiporter activity	response to antibiotic, tetracycline transport	integral to membrane			
PBANKA_123780	multidrug resistance protein, putative (MDR1)	-2.4	0.7	PF3D7_0523000	multidrug resistance protein (MDR1)	transporter	1	1	2	na	ATP binding, ATPase activity, coupled to transmembrane movement of substances	transport	integral to membrane			

PBANKA_082140	ADP-ribosylation factor-like protein	-4.56	0.75	PF3D7_0920500	ADP-ribosylation factor, putative	trafficking	1	1	2	na	GTP binding	small GTPase mediated signal transduction	intracellular
PBANKA_051850	ADP-ribosylation factor, putative	-4.45	0.75	PF3D7_1034700	ADP-ribosylation factor, putative	trafficking	1	1	2	na	GTP binding	intracellular protein transport, small GTPase mediated signal transduction	intracellular
PBANKA_100830	conserved Plasmodium protein, unknown function			PF3D7_1436300	translocon component PTEX150 (PTEX150)	trafficking	1	1	2	na	GTP binding	null	intracellular
PBANKA_142370	GDP-mannose 4,6-dehydratase, putative	-2.74	0.75	PF3D7_0813800	GDP-mannose 4,6-dehydratase, putative	mannose metabolism	1	1	2	na	GDP-mannose 4,6-dehydratase activity, coenzyme binding	GDP-mannose metabolic process	intracellular
PBANKA_122330	GTPase, Rab18, putative			PF3D7_0807300	Rab GTPase 18 (RAB18)	trafficking	1	1	2	na	GTP binding, GTPase activity, protein binding	intracellular protein transport, nucleocytoplasmic transport, small GTPase mediated signal transduction	intracellular
PBANKA_141890	Rab GTPase 11a (Rab11a)	-4.21	0.75	PF3D7_1320600	Rab GTPase 11a (RAB11a)	trafficking	1	1	2	KO not successful	GTP binding, GTPase activity, protein binding	intracellular protein transport, nucleocytoplasmic transport, small GTPase mediated signal transduction	intracellular
PBANKA_030800	Rab5a, GTPase, putative			PF3D7_0211200	Rab GTPase 5a (RAB5a)	trafficking	1	1	2	na	GTP binding, GTPase activity, protein binding	intracellular protein transport, nucleocytoplasmic transport, small GTPase mediated signal transduction	intracellular
PBANKA_041820	Rab7, putative			PF3D7_0903200	Rab GTPase 7 (Rab7)	trafficking	1	1	2	na	GTP binding, GTPase activity, protein binding	intracellular protein transport, nucleocytoplasmic transport, small GTPase mediated signal transduction	intracellular
PBANKA_114350	ribosome biogenesis protein MRT4, putative			PF3D7_1367600	ribosome biogenesis protein MRT4, putative	ribosome assembly	1	1	2	na	null	ribosome biogenesis	intracellular
PBANKA_144580	small GTPase Rab2, putative			PF3D7_1231100	Rab GTPase 2 (RAB2)	trafficking	1	1	2	na	GTP binding, GTPase activity, protein binding	intracellular protein transport, nucleocytoplasmic transport, small GTPase mediated signal transduction	intracellular
PBANKA_071400	meiotic recombination protein DMC1-like protein, putative			PF3D7_0816800	meiotic recombination protein dmc1-like protein	DNA repair	1	1	2	different in fertilization, ookinete, oocyst, sporozoite and liver stage	ATP binding, DNA binding, DNA-dependent ATPase activity, protein binding	reciprocal meiotic recombination	intracellular, nucleus
PBANKA_070910	60S ribosomal protein L22, putative			PF3D7_0821700	60S ribosomal protein L22, putative	ribosome	1	1	2	na	structural constituent of ribosome	translation	intracellular, ribosome
PBANKA_134670	60S ribosomal protein L23, putative			PF3D7_1331800	60S ribosomal protein L23, putative	ribosome	1	1	2	na	structural constituent of ribosome	translation	intracellular, ribosome
PBANKA_090490	conserved Plasmodium protein, unknown function			PF3D7_1144100	conserved protein, unknown function	unknown function	1	1	2	na	structural constituent of ribosome	translation	intracellular, ribosome
PBANKA_124560	mitochondrial ribosomal protein S16 precursor, putative			PF3D7_0531200	mitochondrial ribosomal protein S16 precursor, putative	ribosome mito	1	1	2	na	structural constituent of ribosome	translation	intracellular, ribosome
PBANKA_061600	mitochondrial ribosomal protein S8 precursor, putative			PF3D7_0718400	mitochondrial ribosomal protein S8 precursor, putative	ribosome mito	1	1	2	na	structural constituent of ribosome	translation	intracellular, ribosome
PBANKA_101810	ABC transporter, putative			PF3D7_1426500	ABC transporter, (EPP family), putative	transporter	1	1	2	na	ATP binding, ATPase activity	null	membrane
PBANKA_070670	dicarboxylate/tricarboxylate carrier, putative (DTC)	-5.45	0.75	PF3D7_0823900	dicarboxylate/tricarboxylate carrier (DTC)	transporter	1	1	2	na	binding	transport	membrane
PBANKA_010210	dihydroorotate dehydrogenase, putative (DHODH)	-2.58	0.75	PF3D7_0603300	dihydroorotate dehydrogenase, mitochondrial precursor (DHODH)	pyrimidine metabolism	1	1	2	na	dihydroorotate dehydrogenase activity, dihydroorotate oxidase activity	'de novo' pyrimidine nucleobase biosynthetic process, UMP biosynthetic process	membrane
PBANKA_135900	sec61 alpha subunit, putative			PF3D7_1346100	Sec61 alpha subunit, PfSec61 (SEC61)	trafficking	1	1	2	na	P-P-bond-hydrolysis-driven protein transmembrane transporter activity	protein transport	membrane
PBANKA_030690	Sec61-gamma subunit of protein translocation complex, putative	-3.5	0.75	PF3D7_0210000	secretory complex protein 61 gamma subunit (Sec61-gamma)	trafficking	1	1	2	na	P-P-bond-hydrolysis-driven protein transmembrane transporter activity	protein targeting	membrane
PBANKA_052350	clathrin assembly protein, putative			PF3D7_0423100	clathrin assembly protein, putative	trafficking	1	1	2	na	protein binding, protein transporter activity	intracellular protein transport, vesicle-mediated transport	membrane coat
PBANKA_143230	cell traversal protein for ookinetes and sporozoites (CelTOS)			PF3D7_1216600	cell traversal protein for ookinetes and sporozoites (CelTOS)	adhesin	1	1	2	different in fertilization, ookinete, oocyst, sporozoite and liver stage	null	null	microneme
PBANKA_060710	tim10 homologue, putative			PF3D7_1208600	tim10 homologue, putative	trafficking	1	1	2	na	null	protein import into mitochondrial inner membrane	mitochondrial intermembrane space protein transporter complex
PBANKA_110260	zinc binding protein, putative			PF3D7_0502900	mitochondrial inner membrane TIM10 associated protein, putative	trafficking	1	1	2	na	null	protein import into mitochondrial inner membrane	mitochondrial intermembrane space protein transporter complex
PBANKA_082880	cytochrome c oxidase, putative			PF3D7_0928000.1/2	cytochrome c oxidase, putative	redoxmeth	1	1	2	na	cytochrome-c oxidase activity	null	mitochondrion
PBANKA_135570	myosin A (MyoA)	-4.46	0.75	PF3D7_1342600	myosin A (MyoA)	gliding motility	1	1	2	(mutated) different in fertilization, ookinete, oocyst and sporozoite	ATP binding, motor activity	null	myosin complex
PBANKA_144940	Splicing factor 3B subunit 3, putative (SF3B3)			PF3D7_1234800	Splicing factor 3B subunit 3, putative (SF3B3)	mRNA	1	1	2	na	nucleic acid binding	null	nucleus
PBANKA_090590	transcription factor with AP2 domain(s) (AP2-O)	-3.76	0.75	PF3D7_1143100	transcription factor with AP2 domain(s) (AP2-O)	transcription	1	1	2	different in fertilization, ookinete, oocyst and sporozoite	sequence-specific DNA binding transcription factor activity	regulation of transcription, DNA-dependent	nucleus
PBANKA_092880	U2 snRNP auxiliary factor, small subunit, putative			PF3D7_1119300	U2 snRNP auxiliary factor, small subunit, putative	mRNA	1	1	2	na	RNA binding, zinc ion binding	null	nucleus
PBANKA_130820	1-acyl-sn-glycerol-3-phosphate acyltransferase, putative (LPAAT)			PF3D7_1444300	apicoplast 1-acyl-sn-glycerol-3-phosphate acyltransferase, putative	fatty acids synth	1	1	2	KO not successful	transferase activity, transferring acyl groups	metabolic process	null

PBANKA_100210	6-cysteine protein (P36)	-2.55	0.73	PF3D7_0404400	6-cysteine protein (P36)	adhesin	1	1	2	different in liver stage	null	null	null	null	null	null
PBANKA_100260	6-cysteine protein (P41)			PF3D7_0404900	6-cysteine protein (P41)	adhesin	1	1	2	not different from wild type	null	null	null	null	null	null
PBANKA_145330	acyl-CoA synthetase, putative	-2.63	0.75	PF3D7_1238800	acyl-CoA synthetase, PfACS11 (ACS11)	lipid metabolism	1	1	2	na	catalytic activity	metabolic process	null			
PBANKA_122560	alpha/beta hydrolase, putative	-1.96	0.73	PF3D7_0805000	alpha/beta hydrolase, putative	chaperone	1	1	2	na	null	null	null	null	null	apicoplast
PBANKA_121690	ATP-dependent RNA helicase, putative			PF3D7_0321600	ATP-dependent RNA helicase, putative	mRNA	1	1	2	na	ATP binding, ATP-dependent helicase activity, helicase activity, nucleic acid binding	null	null			
PBANKA_041570	autophagy-related protein 3, putative (ATG3)	-1.86	0.72	PF3D7_0905700.1/2/3	autophagy-related protein 3, putative (ATG3)	autophagy	1	1	2	na	null	null	null	null	null	null
PBANKA_090040	BIR protein					BIR protein	1	1	2	na	null	null	null	na	na	na
PBANKA_146520	BIR protein					BIR protein	1	1	2	na	null	null	null			
PBANKA_021500	BIR protein, pseudogene					BIR protein	1	1	2	na	null	null	null	na	na	na
PBANKA_060620	blood stage antigen 41-3 precursor, putative	-5.88	0.75	PF3D7_1207700	blood stage antigen 41-3 precursor		1	1	2	na	null	null	null	null	null	null
PBANKA_141110	branched-chain alpha keto-acid dehydrogenase, putative			PF3D7_1312600	branched-chain alpha keto-acid dehydrogenase, putative	TCA	1	1	2	na	oxidoreductase activity, acting on the aldehyde or oxo group of donors, disulfide as acceptor	metabolic process	null			
PBANKA_081590	BSD domain, putative			PF3D7_0914900	BSD domain, putative	transcription	1	1	2	na	null	null	null	null	null	null
PBANKA_102440	calmodulin, putative	-2.14	0.75	PF3D7_1418300	calmodulin, putative	Ca2+ binding	1	1	2	na	null	null	null	calcium ion binding	null	null
PBANKA_113320	cdc2-related kinase 2 (CRK2)			PF3D7_1356900	protein kinase 5 (PK5)	kinase	1	1	2	different in liver stage	ATP binding, protein serine/threonine kinase activity, protein tyrosine kinase activity	protein phosphorylation	null			
PBANKA_124400	cell cycle regulator protein, putative			PF3D7_0529500	cell cycle regulator protein, putative	RNA binding	1	1	2	na	RNA binding	null	null			
PBANKA_112190	chorismate synthase, putative (CS)			PF3D7_0623000	chorismate synthase (CS)	shikimate	1	1	2	na	chorismate synthase activity	aromatic amino acid family biosynthetic process	null			
PBANKA_041290	circumsporozoite- and TRAP-related protein (CTRP)			PF3D7_0315200	circumsporozoite- and TRAP-related protein (CTRP)	adhesin	1	1	2	different in fertilization, ookinete and oocyst	null	null	null	null	null	null
PBANKA_123520	conserved Plasmodium protein, unknown function			PF3D7_0520400	conserved Plasmodium protein, unknown function	unknown function	1	1	2	na	DNA binding	regulation of transcription, DNA-dependent	null			
PBANKA_030580	conserved Plasmodium protein, unknown function			PF3D7_0208700.1/2	conserved Plasmodium protein, unknown function	unknown function	1	1	2	na	DNA-directed RNA polymerase activity	transcription, DNA-dependent	null			
PBANKA_020360	conserved Plasmodium protein, unknown function	-6.26	0.75	PF3D7_0110000	conserved Plasmodium protein, unknown function	unknown function	1	1	2	na	null	null	null	null	null	null
PBANKA_030160	conserved Plasmodium protein, unknown function	-5.31	0.75	PF3D7_0203800	conserved Plasmodium protein, unknown function	phototransduction pathway	1	1	2	na	null	null	null	null	null	null
PBANKA_030660	conserved Plasmodium protein, unknown function	-2.39	0.74	PF3D7_0209500	conserved Plasmodium protein, unknown function	unknown function	1	1	2	na	null	null	null	GTP binding, GTPase activity, translation initiation factor activity	translation, translational initiation	cytoplasm, intracellular
PBANKA_031070	conserved Plasmodium protein, unknown function			PF3D7_0213800	conserved Plasmodium protein, unknown function	unknown function	1	1	2	na	null	null	null	null	null	null
PBANKA_031210	conserved Plasmodium protein, unknown function			PF3D7_0215400	conserved Plasmodium protein, unknown function	unknown function	1	1	2	na	null	null	null	null	null	membrane
PBANKA_031480	conserved Plasmodium protein, unknown function			PF3D7_0218100	conserved Plasmodium membrane protein, unknown function	unknown function	1	1	2	na	null	null	null	hydrolase activity, metal ion binding, nucleotide binding	transport	integral to membrane
PBANKA_031490	conserved Plasmodium protein, unknown function			PF3D7_0218200	conserved Plasmodium protein, unknown function	unknown function	1	1	2	na	null	null	null	rRNA (guanine-N1-)-methyltransferase activity	ribosomal large subunit assembly	apicoplast
PBANKA_040250	conserved Plasmodium protein, unknown function	-4.21	0.75	PF3D7_0303900	conserved Plasmodium protein, unknown function	unknown function	1	1	2	na	null	null	null	null	null	null
PBANKA_041720	conserved Plasmodium protein, unknown function	-3.16	0.75	PF3D7_0904200	conserved Plasmodium protein, unknown function	unknown function	1	1	2	na	null	null	null	null	null	apicoplast, membrane
PBANKA_050520	conserved Plasmodium protein, unknown function			PF3D7_1021000	conserved Plasmodium protein, unknown function	unknown function	1	1	2	na	null	null	null	glutaminyl-tRNA synthase (glutamine-hydrolyzing) activity	translation	null
PBANKA_050610	conserved Plasmodium protein, unknown function					proteasome	1	1	2	na	null	null	null	na	na	na
PBANKA_050750	conserved Plasmodium protein, unknown function	-1.77	0.7	PF3D7_1023300	conserved Plasmodium protein, unknown function	unknown function	1	1	2	na	null	null	null	null	null	membrane

PBANKA_051300	conserved Plasmodium protein, unknown function			PF3D7_1028900	conserved Plasmodium protein, unknown function	unknown function	1	1	2	na	null	null	null	null	null	membrane
PBANKA_052110	conserved Plasmodium protein, unknown function			PF3D7_1038200	conserved Plasmodium protein, unknown function	unknown function	1	1	2	na	null	null	null	null	null	null
PBANKA_060330	conserved Plasmodium protein, unknown function			PF3D7_1204400	conserved Plasmodium membrane protein, unknown function	unknown function	1	1	2	na	null	null	null	na	na	na
PBANKA_061240	conserved Plasmodium protein, unknown function	-4.07	0.75	PF3D7_0411000	conserved Plasmodium protein, unknown function	unknown function	1	1	2	na	null	null	null	null	null	apicoplast, integral to membrane, membrane
PBANKA_061780	conserved Plasmodium protein, unknown function	-2.09	0.75	PF3D7_0720300	conserved Plasmodium protein, unknown function	unknown function	1	1	2	na	null	null	null	null	null	null
PBANKA_062030	conserved Plasmodium protein, unknown function	-4.34	0.75	PF3D7_0722800	conserved Plasmodium protein, unknown function	unknown function	1	1	2	na	null	null	null	null	null	null
PBANKA_062040	conserved Plasmodium protein, unknown function	-3.41	0.75	PF3D7_0722900	conserved Plasmodium protein, unknown function	unknown function	1	1	2	na	null	null	null	null	null	null
PBANKA_070330	conserved Plasmodium protein, unknown function	-3.25	0.75	PF3D7_0827400	conserved Plasmodium protein, unknown function	unknown function	1	1	2	na	null	null	null	null	null	cytoplasm
PBANKA_070490	conserved Plasmodium protein, unknown function			PF3D7_0825700	conserved Plasmodium protein, unknown function	unknown function	1	1	2	na	null	null	null	null	null	null
PBANKA_071220	conserved Plasmodium protein, unknown function	-2.64	0.75	PF3D7_0818600	conserved Plasmodium protein, unknown function	unknown function	1	1	2	different in oocyst, sporozoite and liver stage	null	null	null	hydrolase activity	null	membrane
PBANKA_072090	conserved Plasmodium protein, unknown function	-4.34	0.75	PF3D7_0418800	conserved Plasmodium protein, unknown function	unknown function	1	1	2	na	null	null	null	null	null	cytoplasm, membrane
PBANKA_080290	conserved Plasmodium protein, unknown function			PF3D7_0705200	conserved Plasmodium protein, unknown function	unknown function	1	1	2	na	null	null	null	null	null	null
PBANKA_080380	conserved Plasmodium protein, unknown function			PF3D7_0706200	conserved Plasmodium protein, unknown function	unknown function	1	1	2	na	null	null	null	null	null	null
PBANKA_081330	conserved Plasmodium protein, unknown function	-3.35	0.75	PF3D7_0912200	conserved Plasmodium membrane protein, unknown function	unknown function	1	1	2	na	null	null	null	null	null	integral to membrane, membrane
PBANKA_081540	conserved Plasmodium protein, unknown function	-5.12	0.75	PF3D7_0914400	conserved Plasmodium protein, unknown function	unknown function	1	1	2	na	null	null	null	nucleotide binding	null	cytoplasm
PBANKA_082120	conserved Plasmodium protein, unknown function			PF3D7_0920300	conserved Plasmodium protein, unknown function	unknown function	1	1	2	na	null	null	null	null	null	cytoplasm
PBANKA_082320	conserved Plasmodium protein, unknown function	-3.1	0.73	PF3D7_0922300	conserved Plasmodium protein, unknown function	unknown function	1	1	2	na	null	null	null	null	null	null
PBANKA_082590	conserved Plasmodium protein, unknown function			PF3D7_0925100	conserved Plasmodium protein, unknown function	unknown function	1	1	2	na	null	null	null	null	null	integral to membrane, membrane
PBANKA_082740	conserved Plasmodium protein, unknown function	-4.21	0.75	PF3D7_0926600	conserved Plasmodium membrane protein, unknown function	unknown function	1	1	2	na	null	null	null	null	null	integral to membrane, membrane
PBANKA_083280	conserved Plasmodium protein, unknown function			PF3D7_0932000	conserved Plasmodium protein, unknown function	unknown function	1	1	2	na	null	null	null	null	null	integral to membrane, membrane
PBANKA_083470	conserved Plasmodium protein, unknown function			PF3D7_0933900	conserved Plasmodium protein, unknown function	unknown function	1	1	2	na	null	null	null	ATP binding, DNA-directed DNA polymerase activity, RNA binding, aspartic-type endopeptidase activity, magnesium ion binding, nucleotidyltransferase activity, peptidase activity, ribonuclease H activity, zinc ion binding	DNA integration, DNA recombination, proteolysis, transposition	cytoplasm, nucleus
PBANKA_090170	conserved Plasmodium protein, unknown function			PF3D7_1147400	conserved Plasmodium protein, unknown function	unknown function	1	1	2	na	null	null	null	protein farnesyltransferase activity, zinc ion binding	protein farnesylation	protein farnesyltransferase complex
PBANKA_090300	conserved Plasmodium protein, unknown function			PF3D7_1146100	conserved Plasmodium protein, unknown function	unknown function	1	1	2	na	null	null	null	null	null	membrane
PBANKA_090620	conserved Plasmodium protein, unknown function			PF3D7_1142800	conserved Plasmodium protein, unknown function	unknown function	1	1	2	na	null	null	null	null	null	null
PBANKA_090780	conserved Plasmodium protein, unknown function	-1.93	0.73	PF3D7_1141200	conserved Plasmodium protein, unknown function	unknown function	1	1	2	na	null	null	null	transferase activity	null	cytoplasm, membrane
PBANKA_091320	conserved Plasmodium protein, unknown function			PF3D7_1135300	conserved Plasmodium membrane protein, unknown function	unknown function	1	1	2	na	null	null	null	null	null	integral to membrane
PBANKA_091670	conserved Plasmodium protein, unknown function	-3.42	0.75	PF3D7_1131500	conserved Plasmodium protein, unknown function	unknown function	1	1	2	na	null	null	null	ATP binding, hydrolase activity	null	cytoplasm
PBANKA_093260	conserved Plasmodium protein, unknown function			PF3D7_1115100	conserved Plasmodium protein, unknown function	unknown function	1	1	2	na	null	null	null	null	null	null
PBANKA_100170	conserved Plasmodium protein, unknown function			PF3D7_0404200	conserved Plasmodium protein, unknown function	unknown function	1	1	2	na	null	null	null	null	null	null
PBANKA_100190	conserved Plasmodium protein, unknown function					unknown function	1	1	2	na	null	null	null	null	null	membrane
PBANKA_102680	conserved Plasmodium protein, unknown function			PF3D7_1415900	conserved Plasmodium protein, unknown function	nucleic acid binding	1	1	2	na	null	null	null	zinc ion binding	null	nucleus

PBANKA_103300	conserved Plasmodium protein, unknown function			PF3D7_1409400	conserved Plasmodium membrane protein, unknown function	unknown function	1	1	2	na	null	null	null	na	na	na
PBANKA_103880	conserved Plasmodium protein, unknown function			PF3D7_1403200	conserved Plasmodium protein, unknown function	unknown function	1	1	2	na	null	null	null	null	null	extracellular region, membrane
PBANKA_110410	conserved Plasmodium protein, unknown function	-8.06	0.75	PF3D7_0504500	conserved Plasmodium protein, unknown function	unknown function	1	1	2	na	null	null	null	null	attachment of GPI anchor to protein	cytoplasm
PBANKA_110600	conserved Plasmodium protein, unknown function			PF3D7_0506400	conserved Plasmodium protein, unknown function	unknown function	1	1	2	na	null	null	null	null	null	null
PBANKA_110930	conserved Plasmodium protein, unknown function	-3.59	0.75	PF3D7_0509700	conserved Plasmodium protein, unknown function	unknown function	1	1	2	na	null	null	null	null	null	apicoplast
PBANKA_110990	conserved Plasmodium protein, unknown function	-2.15	0.73	PF3D7_0510300	conserved Plasmodium protein, unknown function	unknown function	1	1	2	na	null	null	null	null	null	cytoplasm
PBANKA_111020	conserved Plasmodium protein, unknown function	-4.6	0.75	PF3D7_0510700	conserved Plasmodium protein, unknown function	unknown function	1	1	2	na	null	null	null	null	null	null
PBANKA_111270	conserved Plasmodium protein, unknown function			PF3D7_0513000	conserved Plasmodium protein, unknown function	unknown function	1	1	2	na	null	null	null	null	null	null
PBANKA_111500	conserved Plasmodium protein, unknown function	-3.83	0.75	PF3D7_0515400	conserved Plasmodium protein, unknown function	unknown function	1	1	2	na	null	null	null	null	null	null
PBANKA_112590	conserved Plasmodium protein, unknown function	-2.28	0.75			unknown function	1	1	2	na	null	null	null	na	na	na
PBANKA_113230	conserved Plasmodium protein, unknown function			PF3D7_1356000	conserved Plasmodium protein, unknown function	unknown function	1	1	2	na	null	null	null	ATP binding, hydrolase activity	null	null
PBANKA_113580	conserved Plasmodium protein, unknown function			PF3D7_1359500	conserved Plasmodium protein, unknown function	unknown function	1	1	2	na	null	null	null	null	null	null
PBANKA_113740	conserved Plasmodium protein, unknown function			PF3D7_1361300	conserved Plasmodium protein, unknown function	unknown function	1	1	2	na	null	null	null	null	null	membrane
PBANKA_113860	conserved Plasmodium protein, unknown function	-4.57	0.73	PF3D7_1362600	conserved Plasmodium protein, unknown function	unknown function	1	1	2	na	null	null	null	null	null	null
PBANKA_114380	conserved Plasmodium protein, unknown function	-4.19	0.75	PF3D7_1367900	conserved Plasmodium protein, unknown function	unknown function	1	1	2	na	null	null	null	null	null	apicoplast, membrane
PBANKA_120450	conserved Plasmodium protein, unknown function			PF3D7_1006300	conserved Plasmodium protein, unknown function	unknown function	1	1	2	na	null	null	null	null	null	null
PBANKA_120930	conserved Plasmodium protein, unknown function			PF3D7_1010900	conserved Plasmodium protein, unknown function	unknown function	1	1	2	na	null	null	null	ATP binding, protein kinase activity	protein phosphorylation	Golgi apparatus, cytoplasm, cytoplasmic vesicle, membrane
PBANKA_121440	conserved Plasmodium protein, unknown function	-3.47	0.75	PF3D7_1016000	conserved Plasmodium protein, unknown function	unknown function	1	1	2	na	null	null	null	ATP binding, protein binding	null	cytoplasm, plasma membrane
PBANKA_122530	conserved Plasmodium protein, unknown function	-2.93	0.72	PF3D7_0805300	conserved Plasmodium protein, unknown function	unknown function	1	1	2	na	null	null	null	N-acetylgalactosaminyl-proteoglycan 3-beta-glucuronosyltransferase activity, zinc ion binding	null	intracellular, plasma membrane
PBANKA_122550	conserved Plasmodium protein, unknown function	-2.7	0.73	PF3D7_0805100	conserved Plasmodium protein, unknown function	unknown function	1	1	2	na	null	null	null	null	null	null
PBANKA_122750	conserved Plasmodium protein, unknown function			PF3D7_0802700	conserved Plasmodium protein, unknown function	unknown function	1	1	2	na	null	null	null	null	null	null
PBANKA_122990	conserved Plasmodium protein, unknown function	-2.38	0.72	PF3D7_0615200	conserved Plasmodium protein, unknown function	unknown function	1	1	2	na	null	null	null	null	null	null
PBANKA_123900	conserved Plasmodium protein, unknown function			PF3D7_0524200	conserved Plasmodium membrane protein, unknown function	unknown function	1	1	2	na	null	null	null	null	null	integral to membrane, membrane
PBANKA_124010	conserved Plasmodium protein, unknown function	-3.8	0.75	PF3D7_0525300	conserved Plasmodium protein, unknown function	unknown function	1	1	2	na	null	null	null	null	null	null
PBANKA_124240	conserved Plasmodium protein, unknown function			PF3D7_0527600	conserved Plasmodium protein, unknown function	DNA replication	1	1	2	na	null	null	null	null	null	null
PBANKA_124260	conserved Plasmodium protein, unknown function			PF3D7_0528000	conserved Plasmodium protein, unknown function	proteasome	1	1	2	na	null	null	null	null	null	cytoplasm
PBANKA_124520	conserved Plasmodium protein, unknown function	-2.94	0.73	PF3D7_0530800	CPW-WPC family protein	adhesin	1	1	2	na	null	null	null	null	null	null
PBANKA_130980	conserved Plasmodium protein, unknown function			PF3D7_1446100	conserved Plasmodium protein, unknown function	unknown function	1	1	2	na	null	null	null	null	null	cytoplasm
PBANKA_131130	conserved Plasmodium protein, unknown function	-2.11	0.75	PF3D7_1447500	conserved Plasmodium protein, unknown function	unknown function	1	1	2	na	null	null	null	null	null	cytoplasm
PBANKA_131140	conserved Plasmodium protein, unknown function			PF3D7_1447600	conserved Plasmodium protein, unknown function	unknown function	1	1	2	na	null	null	null	null	null	null
PBANKA_131260	conserved Plasmodium protein, unknown function			PF3D7_1448900	conserved Plasmodium protein, unknown function	unknown function	1	1	2	na	null	null	null	DNA binding, metal ion binding	regulation of transcription, DNA-dependent, transcription, DNA-dependent	nucleus

PBANKA_131850	conserved Plasmodium protein, unknown function	-5.23	0.75	PF3D7_1454800	conserved Plasmodium protein, unknown function	unknown function	1	1	2	na	null	null	null	null	null	null
PBANKA_131860	conserved Plasmodium protein, unknown function	-3.25	0.73	PF3D7_1454900	conserved Plasmodium protein, unknown function	unknown function	1	1	2	not different from wild type	null	null	null	null	null	membrane
PBANKA_131900	conserved Plasmodium protein, unknown function			PF3D7_1455300	conserved Plasmodium protein, unknown function	unknown function	1	1	2	KO not successful	null	null	null	DNA binding, flap endonuclease activity, single-stranded DNA specific 5'-3' exodeoxyribonuclease activity	DNA recombination, mismatch repair, response to DNA damage stimulus	nucleus
PBANKA_132080	conserved Plasmodium protein, unknown function	-5.48	0.75	PF3D7_1457100	conserved Plasmodium protein, unknown function	unknown function	1	1	2	na	null	null	null	null	null	membrane
PBANKA_132180	conserved Plasmodium protein, unknown function			PF3D7_1458100	conserved Plasmodium protein, unknown function	unknown function	1	1	2	na	null	null	null	nucleotide binding, transferase activity	null	membrane
PBANKA_132390	conserved Plasmodium protein, unknown function			PF3D7_1460200	conserved Plasmodium protein, unknown function	unknown function	1	1	2	na	null	null	null	null	null	membrane
PBANKA_132540	conserved Plasmodium protein, unknown function			PF3D7_1461700	conserved Plasmodium protein, unknown function	unknown function	1	1	2	na	null	null	null	ATP binding, protein binding, zinc ion binding	intracellular protein transmembrane transport, protein import, protein targeting	cytoplasm, plasma membrane
PBANKA_132940	conserved Plasmodium protein, unknown function	-4.02	0.75	PF3D7_1466000	conserved Plasmodium protein, unknown function	unknown function	1	1	2	na	null	null	null	null	null	null
PBANKA_132990	conserved Plasmodium protein, unknown function			PF3D7_1466500	conserved Plasmodium protein, unknown function	unknown function	1	1	2	na	null	null	null	null	null	null
PBANKA_133400	conserved Plasmodium protein, unknown function			PF3D7_1470800	conserved Plasmodium protein, unknown function	unknown function	1	1	2	na	null	null	null	null	null	null
PBANKA_133470	conserved Plasmodium protein, unknown function	-3.7	0.75	PF3D7_1471500	conserved Plasmodium membrane protein, unknown function	unknown function	1	1	2	na	null	null	null	null	null	integral to membrane, membrane
PBANKA_133620	conserved Plasmodium protein, unknown function			PF3D7_1473000	conserved Plasmodium protein, unknown function	unknown function	1	1	2	na	null	null	null	null	null	membrane
PBANKA_133810	conserved Plasmodium protein, unknown function			PF3D7_1322900	conserved Plasmodium protein, unknown function	unknown function	1	1	2	na	null	null	null	null	null	null
PBANKA_134230	conserved Plasmodium protein, unknown function	-3.45	0.75	PF3D7_1327100	conserved Plasmodium protein, unknown function	unknown function	1	1	2	na	null	null	null	beta-galactosidase activity, carbohydrate binding, cation binding	carbohydrate metabolic process	beta-galactosidase complex, extracellular region
PBANKA_134370	conserved Plasmodium protein, unknown function			PF3D7_1328600	conserved Plasmodium protein, unknown function	unknown function	1	1	2	na	null	null	null	null	null	cytoplasm
PBANKA_135380	conserved Plasmodium protein, unknown function	-7.77	0.75	PF3D7_1340400	conserved Plasmodium protein, unknown function	unknown function	1	1	2	na	null	null	null	null	null	null
PBANKA_135490	conserved Plasmodium protein, unknown function	-4.62	0.75	PF3D7_1341800	conserved Plasmodium protein, unknown function	gliding motility	1	1	2	na	null	null	null	nucleotide binding	null	cytoplasm
PBANKA_135610	conserved Plasmodium protein, unknown function	-3.07	0.75	PF3D7_1343100	conserved Plasmodium protein, unknown function	unknown function	1	1	2	na	null	null	null	null	null	null
PBANKA_136040	conserved Plasmodium protein, unknown function			PF3D7_1347600	conserved Plasmodium protein, unknown function	unknown function	1	1	2	na	null	null	null	null	null	null
PBANKA_136140	conserved Plasmodium protein, unknown function			PF3D7_1348600	conserved Plasmodium protein, unknown function	unknown function	1	1	2	na	null	null	null	null	null	cytoplasm
PBANKA_136460	conserved Plasmodium protein, unknown function			PF3D7_1351900	conserved Plasmodium protein, unknown function	unknown function	1	1	2	na	null	null	null	ATP binding, hydrolase activity	null	integral to membrane
PBANKA_140090	conserved Plasmodium protein, unknown function			PF3D7_1302400	conserved Plasmodium membrane protein, unknown function	unknown function	1	1	2	na	null	null	null	null	null	endoplasmic reticulum, integral to membrane
PBANKA_141480	conserved Plasmodium protein, unknown function	-3.46	0.75	PF3D7_1316300	conserved Plasmodium protein, unknown function	unknown function	1	1	2	na	null	null	null	null	null	null
PBANKA_141520	conserved Plasmodium protein, unknown function	-3.5	0.75	PF3D7_1316700	conserved Plasmodium protein, unknown function	unknown function	1	1	2	na	null	null	null	nucleotide binding	null	null
PBANKA_141540	conserved Plasmodium protein, unknown function	-2.34	0.73	PF3D7_1316900	conserved Plasmodium protein, unknown function	chromatin	1	1	2	na	null	null	null	protein binding	DNA repair, regulation of transcription, DNA-dependent, response to DNA damage stimulus, transcription, DNA-dependent	nucleus
PBANKA_141740	conserved Plasmodium protein, unknown function	-5.81	0.75	PF3D7_1318900	conserved Plasmodium protein, unknown function	unknown function	1	1	2	na	null	null	null	ATP binding	null	null
PBANKA_141930	conserved Plasmodium protein, unknown function	-2.85	0.75	PF3D7_1321000	conserved Plasmodium protein, unknown function	unknown function	1	1	2	na	null	null	null	null	transport	integral to membrane, plasma membrane
PBANKA_142200	conserved Plasmodium protein, unknown function	-1.89	0.73	PF3D7_0715700	conserved Plasmodium protein, unknown function	unknown function	1	1	2	na	null	null	null	DNA binding, nucleotidyltransferase activity	null	null
PBANKA_142290	conserved Plasmodium protein, unknown function	-5.15	0.75	PF3D7_0814600	conserved Plasmodium protein, unknown function	unknown function	1	1	2	na	null	null	null	ATP binding, transferase activity	null	null
PBANKA_142860	conserved Plasmodium protein, unknown function			PF3D7_1212600.1	conserved Plasmodium membrane protein, unknown function	unknown function	1	1	2	na	null	null	null	null	null	integral to membrane, membrane
PBANKA_143030	conserved Plasmodium protein, unknown function			PF3D7_1214500	conserved Plasmodium protein, unknown function	unknown function	1	1	2	na	null	null	null	null	null	apicoplast

PBANKA_143070	conserved Plasmodium protein, unknown function			PF3D7_1214900	conserved Plasmodium membrane protein, unknown function	unknown function	1	1	2	na	null	null	null	L-malate dehydrogenase activity, binding	glycolysis, malate metabolic process, tricarboxylic acid cycle	integral to membrane
PBANKA_143500	conserved Plasmodium protein, unknown function			PF3D7_1219800	conserved Plasmodium protein, unknown function	unknown function	1	1	2	na	null	null	null	null	null	integral to membrane, plasma membrane
PBANKA_143630	conserved Plasmodium protein, unknown function	-2.46	0.73	PF3D7_1221100	conserved Plasmodium protein, unknown function	unknown function	1	1	2	na	null	null	null	null	null	null
PBANKA_143640	conserved Plasmodium protein, unknown function			PF3D7_1221200	conserved Plasmodium protein, unknown function	unknown function	1	1	2	na	null	null	null	ATP binding, ATPase activity, coupled to transmembrane movement of substances, chloride channel activity, chloride ion binding	transport	integral to membrane
PBANKA_143770	conserved Plasmodium protein, unknown function			PF3D7_1222800	conserved Plasmodium protein, unknown function	unknown function	1	1	2	na	null	null	null	null	null	cytoplasm
PBANKA_144420	conserved Plasmodium protein, unknown function			PF3D7_1229600	conserved Plasmodium protein, unknown function	unknown function	1	1	2	na	null	null	null	protein binding	null	apicoplast, cytoplasm
PBANKA_144480	conserved Plasmodium protein, unknown function	-4.42	0.75	PF3D7_1230100	conserved Plasmodium protein, unknown function	unknown function	1	1	2	na	null	null	null	null	null	null
PBANKA_144640	conserved Plasmodium protein, unknown function			PF3D7_1231700	conserved Plasmodium protein, unknown function	unknown function	1	1	2	na	null	null	null	null	null	null
PBANKA_145210	conserved Plasmodium protein, unknown function			PF3D7_1237500	conserved Plasmodium protein, unknown function	unknown function	1	1	2	na	null	null	null	null	null	apicoplast, cytoplasm, membrane
PBANKA_145390	conserved Plasmodium protein, unknown function	-1.76	0.7	PF3D7_1239400	conserved Plasmodium protein, unknown function	unknown function	1	1	2	na	null	null	null	ATP binding, ligase activity	translation	cytoplasm
PBANKA_146130	conserved Plasmodium protein, unknown function	-5.22	0.75	PF3D7_1248400	conserved Plasmodium protein, unknown function	unknown function	1	1	2	na	null	null	null	null	null	apicoplast
PBANKA_146330	conserved Plasmodium protein, unknown function	-5.38	0.75	PF3D7_1250400	conserved Plasmodium protein, unknown function	unknown function	1	1	2	na	null	null	null	ATP binding, actin binding, calmodulin binding, motor activity	null	cytoplasm, myosin complex
PBANKA_100440	conserved Plasmodium protein, unknown function	-1.75	0.73	PF3D7_0406800	conserved Plasmodium protein, unknown function	unknown function	1	1	2	na	null	translation	null			
PBANKA_132380	conserved Plasmodium protein, unknown function	-5.5	0.75			unknown function	1	1	2	na	protein binding	null	null			
PBANKA_052190	conserved Plasmodium protein, unknown function			PF3D7_0420500	conserved Plasmodium protein, unknown function	phosphatase	1	1	2	na	protein tyrosine/serine/threonine phosphatase activity	protein dephosphorylation	null			
PBANKA_093040	conserved Plasmodium protein, unknown function			PF3D7_1117600.1/2	conserved Plasmodium protein, unknown function	translation	1	1	2	na	translation release factor activity	translational termination	null			
PBANKA_101540	CPW-WPC family protein	-2.61	0.73	PF3D7_1429300	CPW-WPC family protein	adhesin	1	1	2	na	null	null	null	null	null	null
PBANKA_112320	CPW-WPC family protein			PF3D7_0624300	CPW-WPC family protein	adhesin	1	1	2	na	null	null	null	null	null	membrane
PBANKA_135250	CPW-WPC family protein	-3.01	0.75	PF3D7_1338800	CPW-WPC family protein	adhesin	1	1	2	na	null	null	null	null	null	null
PBANKA_134630	CPW-WPC family protein (UIS19)	-3.77	0.73	PF3D7_1331400	CPW-WPC family protein	adhesin	1	1	2	na	null	null	null	null	null	apicoplast
PBANKA_094340	CPW-WPC family protein, putative	-2.54	0.75	PF3D7_1103500	CPW-WPC family protein	adhesin	1	1	2	na	null	null	null	null	null	apicoplast
PBANKA_144930	CPW-WPC family protein, putative	-2.93	0.74	PF3D7_1234700	CPW-WPC family protein	adhesin	1	1	2	na	null	null	null	null	null	null
PBANKA_132170	cysteine proteinase, putative			PF3D7_1458000	cysteine proteinase falcipain 1	protease	1	1	2	na	cysteine-type peptidase activity	proteolysis	null			
PBANKA_081910	cytochrome b5, putative			PF3D7_0918100	cytochrome b5-like heme/steroid binding protein, putative	utilization phospholipids	1	1	2	na	heme binding	null	null			
PBANKA_141020	cytochrome c2 precursor,putative			PF3D7_1311700	cytochrome c2 precursor, putative	redoxmeth	1	1	2	na	electron carrier activity, heme binding, iron ion binding	null	null			
PBANKA_051200	DHHC-type zinc finger protein, putative	-3.85	0.75	PF3D7_1027900	DHHC-type zinc finger protein, putative	palmytoylation; prenylation	1	1	2	na	zinc ion binding	null	null			
PBANKA_133460	diacylglycerol kinase, putative			PF3D7_1471400	diacylglycerol kinase, putative	utilization phospholipids	1	1	2	na	diacylglycerol kinase activity	protein kinase C-activating G-protein coupled receptor signaling pathway	null			
PBANKA_080620	e3 ubiquitin-protein ligase rbx1, putative (RBX1)			PF3D7_0319100	ubiquitin-protein ligase, putative	proteasome	1	1	2	na	protein binding, zinc ion binding	null	null			
PBANKA_122950	endonuclease III homologue, putative			PF3D7_0614800	endonuclease III homologue, putative	DNA repair	1	1	2	na	4 iron, 4 sulfur cluster binding, endonuclease activity	base-excision repair	null			
PBANKA_124280	eukaryotic translation initiation factor 3, subunit 6, putative			PF3D7_0528200	eukaryotic translation initiation factor 3, subunit 6, putative	translation	1	1	2	na	null	null	null	null	null	null
PBANKA_062250	exonuclease I, putative			PF3D7_0725000	exonuclease I, putative	DNA repair	1	1	2	na	DNA binding, nuclease activity	DNA repair	null			

PBANKA_052390	glideosome associated protein with multiple membrane spans 2, putative (GAPM2)			PF3D7_0423500	glideosome associated protein with multiple membrane spans 2 (GAPM2)	gliding motility	1	1	2	na	null	null	null	metal ion binding, protein binding	transport	actomyosin, actin part, inner membrane complex, integral to membrane, membrane
PBANKA_103540	glideosome associated protein with multiple membrane spans 3, putative	-2.56	0.7	PF3D7_1406800	glideosome associated protein with multiple membrane spans 3 (GAPM3)	gliding motility	1	1	2	na	null	null	null	null	null	inner membrane complex, integral to membrane, membrane
PBANKA_111530	glideosome-associated protein 40, putative (GAP40)	-4.69	0.75	PF3D7_0515700	glideosome-associated protein 40, putative (GAP40)	gliding motility	1	1	2	na	null	null	null	null	null	null
PBANKA_143760	glideosome-associated protein 45, putative	-4.44	0.74	PF3D7_1222700	glideosome-associated protein 45 (GAP45)	gliding motility	1	1	2	different in fertilization and ookinete	null	null	null	null	null	extrinsic to membrane, host cell membrane, inner membrane complex
PBANKA_145730	glucose inhibited division protein a homologue, putative			PF3D7_1244000	glucose inhibited division protein a homologue, putative	translation	1	1	2	na	flavin adenine dinucleotide binding	tRNA processing	null			
PBANKA_136430	glycerol kinase, putative	-2.86	0.73	PF3D7_1351600	glycerol kinase (GK)	glycolysis	1	1	2	KO not successful	glycerol kinase activity	carbohydrate metabolic process, glycerol-3-phosphate metabolic process	null			
PBANKA_136090	GTPase, putative			PF3D7_1348100	Rab14 GTPase, putative	trafficking	1	1	2	na	GTP binding	protein transport, small GTPase mediated signal transduction	null			
PBANKA_102520	h/aca ribonucleoprotein complex subunit 4, putative (CBF5)			PF3D7_1417500	pseudouridine synthase, putative	RNA	1	1	2	na	RNA binding, pseudouridine synthase activity	RNA processing, pseudouridine synthesis	null			
PBANKA_110190	HCNGP-like protein, putative			PF3D7_0502100	HCNGP-like protein	transcription	1	1	2	na	null	null	null	null	null	null
PBANKA_031000	heat shock 40 kDa protein, putative			PF3D7_0213100	heat shock protein 40, putative	chaperone	1	1	2	na	heat shock protein binding, unfolded protein binding	protein folding	null			
PBANKA_021480	heat shock protein, putative	-3.38	0.75			chaperone	1	1	2	KO not successful	heat shock protein binding, unfolded protein binding	protein folding	null			
PBANKA_120200	inner membrane complex protein 1c, putative (IMC1C, ALV5)	-4.82	0.75	PF3D7_1003600	membrane skeletal protein IMC1-related (ALV5)	gliding motility	1	1	2	na	null	null	null	null	cytoskeleton organization	inner membrane complex, subpellicular network
PBANKA_121910	inner membrane complex protein 1d, putative (IMC1D)	-10.18	0.75	PF3D7_0708600	heat shock protein 86 family protein	gliding motility	1	1	2	na	null	null	null	null	response to heat, response to unfolded protein	null
PBANKA_040270	inner membrane complex protein 1e, putative (IMC1E, ALV2)	-6.85	0.75	PF3D7_0304100	membrane skeletal protein IMC1-related (ALV2)	gliding motility	1	1	2	na	null	null	null	null	null	inner membrane complex
PBANKA_041760	LCCL domain-containing protein (CCp4)	-5.57	0.75	PF3D7_0903800	LCCL domain-containing protein (CCp4)	adhesin	1	1	2	different in oocyst and sporozoite; (tagged) different in fertilization and ookinete	null	null	null	null	null	null
PBANKA_131950	LCCL domain-containing protein CCP2 (CCP2)	-7.76	0.75	PF3D7_1455800	LCCL domain-containing protein (CCp2)	adhesin	1	1	2	different in fertilization, ookinete, oocyst and sporozoite	receptor binding	cell adhesion, signal transduction	null			
PBANKA_111770	malate dehydrogenase, putative (MDH)			PF3D7_0618500	malate dehydrogenase (MDH)	TCA	1	1	2	na	binding, oxidoreductase activity, acting on the CH-OH group of donors, NAD or NADP as acceptor	glycolysis	null			
PBANKA_070710	membrane skeletal protein, putative	-5.38	0.75	PF3D7_0823500	membrane skeletal protein IMC1-related	gliding motility	1	1	2	na	null	null	null	null	null	null
PBANKA_081570	membrane transporter, putative	-4.44	0.75	PF3D7_0914700	transporter, putative	transporter	1	1	2	na	null	null	null	null	null	null
PBANKA_124210	methionine aminopeptidase, putative	-1.99	0.74	PF3D7_0527300	methionine aminopeptidase 1a, putative (MetAP1a)	post-translational modification	1	1	2	na	aminopeptidase activity, metalloexopeptidase activity	cellular process, proteolysis	null			
PBANKA_082600	methyltransferase, putative			PF3D7_0925200	rRNA processing and telomere maintaining methyltransferase, putative	RNA	1	1	2	na	null	null	null	null	null	null
PBANKA_092110	methyltransferase, putative	-1.61	0.7	PF3D7_1127300	methyltransferase, putative	RNA	1	1	2	na	tRNA (guanine-N7-)-methyltransferase activity	tRNA modification	null			
PBANKA_051520	MORN repeat-containing protein 1, putative (MORN1)			PF3D7_1031200	MORN repeat-containing protein 1 (MORN1)	lipid metabolism	1	1	2	na	null	null	null	null	null	null
PBANKA_141430	Myb1 protein, putative			PF3D7_1315800	Myb1-related transcription factor (MYB1)	transcription	1	1	2	na	DNA binding	null	null			
PBANKA_145950	myosin light chain 1, putative,myosin A tail domain interacting protein M'	-3.21	0.74	PF3D7_1246400	myosin light chain 1,myosin A tail domain interacting protein (MTIP)	gliding motility	1	1	2	(mutated) different in fertilization and ookinete	null	null	null	null	null	inner membrane complex
PBANKA_114340	NADH-cytochrome b5 reductase, putative			PF3D7_1367500	NADH-cytochrome b5 reductase, putative	fatty acids synth	1	1	2	na	electron carrier activity, oxidoreductase activity	null	null			
PBANKA_124110	nucleolar Jumonji domain interacting protein, putative	-3.72	0.75	PF3D7_0526300	nucleolar Jumonji domain interacting protein, putative	ribosome	1	1	2	na	null	null	null	null	null	null
PBANKA_090310	nucleolar preribosomal assembly protein, putative	-2.02	0.74	PF3D7_1146000	nucleolar preribosomal assembly protein, putative	ribosome assembly	1	1	2	na	null	null	null	null	null	null
PBANKA_103470	nucleolar preribosomal GTPase, putative			PF3D7_1407500	nucleolar preribosomal GTPase, putative	ribosome assembly	1	1	2	na	null	null	null	null	null	null
PBANKA_103340	oxidoreductase, putative			PF3D7_1409100	aldo-keto reductase, putative	redoxmeth	1	1	2	na	oxidoreductase activity	oxidation-reduction process	null			
PBANKA_020060	Pb-fam-1 protein					Pb-fam-1 protein	1	1	2	na	null	null	null	na	na	na

PBANKA_021490	Pb-fam-1 protein					Pb-fam-1 protein	1	1	2	na	null	null	null	na	na	na
PBANKA_110090	Pb-fam-1 protein	-1.64	0.71			Pb-fam-1 protein	1	1	2	na	null	null	null			
PBANKA_093220	peptidyl-prolyl cis-trans isomerase, putative			PF3D7_1115600	peptidyl-prolyl cis-trans isomerase (CYP19B)	chaperone	1	1	2	not different from wild type	peptidyl-prolyl cis-trans isomerase activity	null	null			
PBANKA_124330	peptidyl-prolyl cis-trans isomerase, putative (CYP23)			PF3D7_0528700	peptidyl-prolyl cis-trans isomerase (CYP23)	chaperone	1	1	2	na	peptidyl-prolyl cis-trans isomerase activity	null	null			
PBANKA_112040	Pfs77 homologue, putative	-4.93	0.75	PF3D7_0621400	Pf77 protein (ALV7)	gliding motility	1	1	2	na	null	null	null	null	null	inner membrane complex
PBANKA_050270	PHF5-like protein, putative			PF3D7_1018500	PHF5-like protein, putative	mRNA	1	1	2	na	null	null	null	null	null	null
PBANKA_143490	phosphatidylethanolamine-binding protein, putative			PF3D7_1219700	raf kinase inhibitor (RKIP)		1	1	2	na	null	null	null	phosphatidylethanolamine binding, protein kinase regulator activity	null	null
PBANKA_133370	phosphodiesterase delta (PDEdelta)	-2.25	0.74	PF3D7_1470500	phosphodiesterase delta, putative (PDEdelta)	purine metabolism	1	1	2	different in fertilization, ookinete and oocyst	3',5'-cyclic-nucleotide phosphodiesterase activity	signal transduction	null			
PBANKA_135590	phosphoenolpyruvate carboxykinase, putative (PEPCK)	-2.49	0.71	PF3D7_1342800	phosphoenolpyruvate carboxykinase (PEPCK)	TCA	1	1	2	na	ATP binding, phosphoenolpyruvate carboxykinase (ATP) activity	gluconeogenesis	null			
PBANKA_050690	phospholipid scramblase, putative			PF3D7_1022700	phospholipid scramblase 1, putative	transporter	1	1	2	na	null	null	null	null	null	null
PBANKA_040970	plasmepsin VI	-2.73	0.73	PF3D7_0311700	plasmepsin VI	plasmepsin, protease	1	1	2	different in oocyst and sporozoite	aspartic-type endopeptidase activity	proteolysis	null			
PBANKA_051760	plasmepsin VII, putative	-5.22	0.75	PF3D7_1033800	plasmepsin VII	plasmepsin, protease	1	1	2	na	aspartic-type endopeptidase activity	proteolysis	null			
PBANKA_132910	plasmepsin VIII, putative	-3.59	0.75	PF3D7_1465700	plasmepsin VIII	plasmepsin, protease	1	1	2	na	aspartic-type endopeptidase activity	proteolysis	null			
PBANKA_020040	Plasmodium exported protein, unknown function					unknown function	1	1	2	na	null	null	null	na	na	na
PBANKA_072260	Plasmodium exported protein, unknown function	-3.76	0.75			unknown function	1	1	2	na	null	null	null			
PBANKA_146510	Plasmodium exported protein, unknown function					unknown function	1	1	2	na	null	null	null			
PBANKA_120510	PPPDE peptidase, putative	-6.22	0.75	PF3D7_1006900	PPPDE peptidase, putative	protease	1	1	2	na	null	null	null	null	null	null
PBANKA_051410	pre-mRNA-splicing factor ATP-dependent RNA helicase PRP22, putative (PRP22)			PF3D7_1030100	pre-mRNA-splicing factor ATP-dependent RNA helicase PRP22, putative (PRP22)	mRNA	1	1	2	na	ATP binding, RNA binding, helicase activity	null	null			
PBANKA_083070	procollagen lysine 5-dioxygenase, putative	-3.61	0.75	PF3D7_0930000	procollagen lysine 5-dioxygenase, putative	post-translational modification	1	1	2	na	null	null	null	null	null	null
PBANKA_141450	protein kinase, putative	-3.93	0.75	PF3D7_1316000	protein kinase, putative	kinase	1	1	2	KO not successful	ATP binding, protein serine/threonine kinase activity, protein tyrosine kinase activity	protein phosphorylation	null			
PBANKA_131880	protein phosphatase 7, putative	-2.1	0.75	PF3D7_1455100	protein phosphatase, putative	phosphatase	1	1	2	na	protein tyrosine/serine/threonine phosphatase activity	protein dephosphorylation	null			
PBANKA_142700	protein phosphatase, putative			PF3D7_0810500	protein phosphatase, putative	phosphatase	1	1	2	na	catalytic activity	null	null			
PBANKA_031100	protein transport protein Sec31, putative (SEC31)			PF3D7_0214100	protein transport protein sec31 (SEC31)	trafficking	1	1	2	na	null	null	null	null	ER to Golgi vesicle-mediated transport, translocation of peptides or proteins into host cell cytoplasm	COPII vesicle coat, ER to Golgi transport vesicle, Maurer's cleft, host cell cytoplasm
PBANKA_070900	protein-transport protein sec61 beta 1 subunit, putative			PF3D7_0821800	secretory complex protein 61 beta subunit (Sec61-beta)	trafficking	1	1	2	na	null	null	null	protein binding, protein transporter activity	intracellular protein transport, protein transport by the Sec complex	Sec61 translocon complex, endoplasmic reticulum membrane
PBANKA_146000	rac-beta serine/threonine protein kinase, putative (PKB)			PF3D7_1246900	RAC-beta serine/threonine protein kinase (PKB)	kinase	1	1	2	KO not successful	ATP binding, protein serine/threonine kinase activity, protein tyrosine kinase activity	protein phosphorylation	null			
PBANKA_093950	Rad51 homolog, putative	-7.81	0.75	PF3D7_1107400	Rad51 homolog (RAD51)	DNA repair	1	1	2	na	ATP binding, DNA-dependent ATPase activity, damaged DNA binding, protein binding	DNA repair	null			
PBANKA_051380	RAP protein, putative			PF3D7_1029800	RAP protein, putative	mRNA	1	1	2	na	null	null	null	null	null	null
PBANKA_140730	recombinase, putative			PF3D7_1308800	recombinase, putative		1	1	2	na	DNA binding	DNA integration, DNA recombination	null			
PBANKA_123010	ribonuclease, putative, fragment						1	1	2	na	null	null	null	RNA binding, ribonuclease activity	RNA processing	null
PBANKA_031580	ribosome associated membrane protein RAMP4, putative			PF3D7_0219400.1/2	ribosome associated membrane protein RAMP4, putative	translation	1	1	2	na	null	null	null	null	null	membrane
PBANKA_112140	RNA methyltransferase, putative			PF3D7_0622500	RNA methyltransferase, putative	RNA	1	1	2	na	methyltransferase activity	rRNA processing	null			

PBANKA_051470	RNA methyltransferase, putative			PF3D7_1030700	RNA methyltransferase, putative	RNA	1	1	2	na	RNA binding, RNA methyltransferase activity	RNA processing	null			
PBANKA_142950	RNA polymerase subunit 8c, putative			PF3D7_1213700	RNA polymerase subunit 8c, putative	mRNA	1	1	2	na	null	transcription, DNA-dependent	null			
PBANKA_071790	RNA-binding protein, putative			PF3D7_0416000	RNA binding protein, putative	mRNA	1	1	2	na	nucleic acid binding	null	null			
PBANKA_120080	RNA-binding protein, putative	-1.66	0.7	PF3D7_1002400.1/2	RNA binding protein, putative	mRNA	1	1	2	na	nucleic acid binding	null	null			
PBANKA_070750	RWD domain-containing protein, putative			PF3D7_0823100	RWD domain-containing protein, putative	proteasome	1	1	2	na	null	null	null	null	null	null
PBANKA_082310	S-adenosylmethionine synthetase, putative	-4.07	0.75	PF3D7_0922200	S-adenosylmethionine synthetase (SAMS)		1	1	2	na	ATP binding, methionine adenosyltransferase activity	one-carbon metabolic process	null			
PBANKA_123360	secreted ookinete protein, putative (PSOP13)			PF3D7_0518800	secreted ookinete protein, putative (PSOP13)	adhesin	1	1	2	different in oocyst and sporozoite	null	null	null	protein binding	null	nucleus
PBANKA_142170	secreted ookinete protein, putative (PSOP20)	-5.61	0.75	PF3D7_0715400	secreted ookinete protein, putative (PSOP20)	adhesin	1	1	2	not different from wild type	null	null	null	null	null	null
PBANKA_112900	secreted ookinete protein, putative (PSOP6)	-2.92	0.74	PF3D7_0630200	secreted ookinete protein, putative (PSOP6)	adhesin	1	1	2	KO not successful	null	null	null	nucleotide binding	null	cytoplasm
PBANKA_102700	serine C-palmitoyltransferase, putative	-3.3	0.75	PF3D7_1415700	serine C-palmitoyltransferase, putative	lipid metabolism	1	1	2	na	pyridoxal phosphate binding, transferase activity, transferring nitrogenous groups	biosynthetic process	null			
PBANKA_030500	serine repeat antigen 2 (SERA2)						1	1	2	not different from wild type	cysteine-type peptidase activity	proteolysis	null			
PBANKA_031140	serine/threonine protein kinase, putative	-4.17	0.75	PF3D7_0214600	serine/threonine protein kinase, putative	kinase	1	1	2	KO not successful	ATP binding, protein serine/threonine kinase activity, protein tyrosine kinase activity	protein phosphorylation	null			
PBANKA_082850	serine/threonine protein phosphatase, putative			PF3D7_0927700	serine/threonine protein phosphatase, putative	phosphatase	1	1	2	na	hydrolase activity	null	null			
PBANKA_145310	sphingomyelin phosphodiesterase, putative	-5.54	0.75	PF3D7_1238600	sphingomyelin phosphodiesterase, putative	lipid metabolism	1	1	2	na	null	null	null	null	null	null
PBANKA_112400	sphingomyelin synthase 2, putative (SMS2)	-7.33	0.75	PF3D7_0625100	sphingomyelin synthase, putative	lipid metabolism	1	1	2	na	null	null	null	null	null	null
PBANKA_081070	subpellicular microtubule protein 1, putative (SPM1)			PF3D7_0909500	subpellicular microtubule protein 1, putative (SPM1)	gliding motility	1	1	2	na	null	null	null	null	null	null
PBANKA_082570	telomeric repeat binding factor 1, putative			PF3D7_0924800	telomeric repeat binding factor 1, putative (TRF1)	telomerase	1	1	2	na	DNA binding	null	null			
PBANKA_132090	thioredoxin, putative (TRX1)			PF3D7_1457200	thioredoxin 1 (TRX1)	redoxmeth	1	1	2	na	electron carrier activity, protein disulfide oxidoreductase activity	cell redox homeostasis, glycerol ether metabolic process	null			
PBANKA_131320	transcription factor with AP2 domain(s), putative (ApiAP2)	-2.49	0.75	PF3D7_1449500	transcription factor with AP2 domain(s), putative (ApiAP2)	transcription	1	1	2	na	sequence-specific DNA binding transcription factor activity	regulation of transcription, DNA-dependent	null			
PBANKA_134760	translation initiation factor 6, putative			PF3D7_1332800	translation initiation factor 6, putative	translation	1	1	2	na	ribosome binding	mature ribosome assembly	null			
PBANKA_124460	triose phosphate transporter, putative			PF3D7_0530200	triose phosphate transporter (iTPT)	transporter	1	1	2	na	null	null	null	glucose 6-phosphate:phosphate antiporter activity, triose-phosphate:phosphate antiporter activity	glucose import	apicoplast, integral to plastid inner membrane, membrane
PBANKA_080650	triosephophate isomerase, putative			PF3D7_0318800	triosephophate isomerase, putative	glycolysis	1	1	2	na	triose-phosphate isomerase activity	metabolic process	null			
PBANKA_103360	tRNA-dihydrouridine synthase, putative	-1.87	0.73	PF3D7_1408900	tRNA-dihydrouridine synthase, putative	translation	1	1	2	na	flavin adenine dinucleotide binding, tRNA dihydrouridine synthase activity	oxidation-reduction process, tRNA processing	null			
PBANKA_071200	U3 small nucleolar ribonucleoprotein, putative			PF3D7_0818800	U3 small nucleolar ribonucleoprotein protein, putative	mRNA	1	1	2	na	null	null	null	null	RNA processing	small nucleolar ribonucleoprotein complex
PBANKA_081610	ubiquitin conjugating enzyme, putative			PF3D7_0915100	ubiquitin conjugating enzyme, putative	proteasome	1	1	2	na	small conjugating protein ligase activity	post-translational protein modification, regulation of protein metabolic process	null			
PBANKA_102970	ubiquitin conjugating enzyme, putative			PF3D7_1412900.1	ubiquitin conjugating enzyme, putative	proteasome	1	1	2	na	small conjugating protein ligase activity	post-translational protein modification, regulation of protein metabolic process	null			
PBANKA_040420	ubiquitin-conjugating enzyme e2, putative			PF3D7_0305700	ubiquitin conjugating enzyme E2, putative	proteasome	1	1	2	na	small conjugating protein ligase activity	post-translational protein modification, regulation of protein metabolic process	null			
PBANKA_122890	von willebrand factor a-domain-related protein (WARP)	-5.64	0.75	PF3D7_0801300	von Willebrand factor A-domain related protein (WARP)	adhesin	1	1	2	not different from wild type	null	null	null	LBD domain binding, cell adhesion molecule binding, heparin binding	cell adhesion	microneme lumen
PBANKA_060860	zinc finger protein, putative	-4.67	0.75	PF3D7_1210200	zinc finger protein, putative	nucleic acid binding	1	1	2	na	nucleic acid binding, zinc ion binding	null	null			
PBANKA_113490	zinc finger protein, putative	-6.57	0.75	PF3D7_1358600	zinc finger protein, putative	RNA binding	1	1	2	na	nucleic acid binding, zinc ion binding	null	null			
PBANKA_143340	zinc finger protein, putative			PF3D7_1217800	zinc finger protein, putative	Ca2+ binding	1	1	2	na	null	null	null	zinc ion binding	null	cytoplasm, nucleus

PBANKA_145800	zinc finger protein, putative			PF3D7_1244800	cytoplasmic translation machinery associated protein, putative	translation	1	1	2	na	null	null	null	null	null	null
PBANKA_010830	zinc finger protein, putative	-3.31	0.75	PF3D7_0609800	zinc finger protein, putative	nucleic acid binding	1	1	2	na	zinc ion binding	null	null			
PBANKA_092730	zinc finger, DHHC-type, putative	-2.88	0.75	PF3D7_1121000	zinc finger, DHHC-type, putative	palmytoylation; prenylation	1	1	2	na	zinc ion binding	null	null			
PBANKA_112550	conserved protein, unknown function			PF3D7_0626700	conserved protein, unknown function	unknown function	1	1	2	na	null	null	null	null	null	null
PBANKA_090010	conserved rodent malaria protein, unknown function					unknown function	1	1	2	na	null	null	null	na	na	na
PBANKA_093150	folate transporter 2, putative (FT2)	-4.21	0.75	PF3D7_1116500	folate transporter 2 (FT2)	transporter	1	1	2	na	folic acid transporter activity	null	plasma membrane			
PBANKA_144510	ATP-dependent protease subunit ClpQ, putative (ClpQ)			PF3D7_1230400	ATP-dependent protease subunit ClpQ (ClpQ)	protease	1	1	2	na	threonine-type endopeptidase activity	proteolysis involved in cellular protein catabolic process	proteasome core complex			
PBANKA_061160	ribonucleoside-diphosphate reductase, large subunit, putative			PF3D7_1437200	ribonucleoside-diphosphate reductase, large subunit	pyrimidine metabolism	1	1	2	na	ATP binding, protein binding, ribonucleoside-diphosphate reductase activity, thioredoxin disulfide as acceptor	DNA replication, oxidation-reduction process	ribonucleoside-diphosphate reductase complex			
PBANKA_140800	h/aca ribonucleoprotein complex subunit 1, putative (GAR1)			PF3D7_1309500	small nucleolar ribonucleoprotein, putative	RNA	1	1	2	na	rRNA binding	rRNA processing	small nucleolar ribonucleoprotein complex			
PBANKA_100310	pre-mRNA-processing-splicing factor 8, putative (PRPF8)			PF3D7_0405400	pre-mRNA-processing-splicing factor 8, putative (PRPF8)	mRNA	1	1	2	na	null	nuclear mRNA splicing, via spliceosome	spliceosomal complex			
PBANKA_083300	profilin, putative (PFN)			PF3D7_0932200	profilin, putative (PFN)	gliding motility	1	0	1	(mutated) different in fertilization and ookinete	actin binding	cytoskeleton organization	actin cytoskeleton			
PBANKA_071690	conserved Plasmodium protein, unknown function			PF3D7_0414900	conserved ARM repeats protein, unknown function	centrosome, chromatin	0	1	1	na	binding	null	anchored to membrane, rhoptry			
PBANKA_110790	triose phosphate transporter, putative			PF3D7_0508300	triose phosphate transporter (oTPT)	transporter, apicoplast	1	0	1	na	null	null	apicoplast, membrane			
PBANKA_143270	conserved Plasmodium protein, unknown function			PF3D7_1217100.1/2	conserved Plasmodium protein, unknown function	meiosis	0	1	1	na	ATP binding, DNA binding, catalytic activity	DNA metabolic process	chromosome			
PBANKA_101140	DNA topoisomerase II, putative			PF3D7_1433500	DNA topoisomerase II, putative	DNA replication	0	1	1	na	ATP binding, DNA topoisomerase (ATP-hydrolyzing) activity	DNA topological change	chromosome			
PBANKA_136000	DNA topoisomerase III, putative			PF3D7_1347100	DNA topoisomerase III, putative	DNA replication	0	1	1	na	DNA topoisomerase type I activity	DNA topological change	chromosome			
PBANKA_113720	Sec24 subunit, putative			PF3D7_1361100	Sec24 subunit a (SEC24a)	trafficking	0	1	1	na	protein binding, zinc ion binding	ER to Golgi vesicle-mediated transport, intracellular protein transport	COPII vesicle coat			
PBANKA_100280	Sec24-like protein, putative			PF3D7_0405100	Sec24 subunit b (SEC24b)	trafficking	1	0	1	na	protein binding	ER to Golgi vesicle-mediated transport, intracellular protein transport	COPII vesicle coat			
PBANKA_030910	peptide chain release factor subunit 1, putative			PF3D7_0212300	peptide chain release factor subunit 1, putative	translation	0	1	1	na	translation release factor activity, codon specific	translational termination	cytoplasm			
PBANKA_111050	translationally controlled tumor protein homolog, putative			PF3D7_0511000	translationally controlled tumor protein homolog, putative	trafficking	1	0	1	na	null	null	cytoplasm			
PBANKA_060280	ubiquitin-conjugating enzyme e2, putative			PF3D7_1203900	ubiquitin conjugating enzyme E2, putative (UBC)	proteasome	1	0	1	na	ubiquitin-protein ligase activity	post-translational protein modification, regulation of protein metabolic process	cytoplasm			
PBANKA_130990	leucine aminopeptidase, putative			PF3D7_1446200	M17 leucyl aminopeptidase (LAP)	protease	0	1	1	na	aminopeptidase activity, manganese ion binding, metalloexopeptidase activity	protein metabolic process, proteolysis	cytoplasm, intracellular			
PBANKA_123210	pre-mRNA-splicing factor, putative (SR1)			PF3D7_0517300	pre-mRNA-splicing factor (SR1)	mRNA	0	1	1	na	nucleic acid binding	RNA splicing	cytoplasm, nucleus			
PBANKA_103620	RNA-binding protein, putative			PF3D7_1406000	RNA binding protein, putative	mRNA	0	1	1	na	RNA binding	RNA processing	cytoplasm, nucleus			
PBANKA_132950	protein serine/threonine phosphatase, putative			PF3D7_1466100	protein serine/threonine phosphatase	phosphatase	1	0	1	different in fertilization, ookinete, oocyst and sporozoite	iron ion binding, manganese ion binding, phosphoprotein phosphatase activity	null	cytoplasm, nucleus			
PBANKA_082340	phosphoglycerate kinase, putative (PGK)			PF3D7_0922500	phosphoglycerate kinase (PGK)	glycolysis	0	1	1	na	phosphoglycerate kinase activity	glycolysis	cytosol			
PBANKA_110860	SNAP protein, putative			PF3D7_0509000	SNAP protein (soluble N-ethylmaleimide-sensitive factor Attachment Protein), putative	trafficking	0	1	1	na	null	intracellular protein transport	Golgi apparatus, endoplasmic reticulum			
PBANKA_120940	conserved Plasmodium protein, unknown function			PF3D7_1011000	conserved protein, unknown function	gliding motility	0	1	1	(tagged) different in fertilization, ookinete, oocyst, sprorozoite and liver stage	null	null	inner membrane complex			
PBANKA_135850	conserved Plasmodium protein, unknown function			PF3D7_1345600	conserved Plasmodium protein, unknown function	gliding motility	1	0	1	na	null	null	inner membrane complex			
PBANKA_110780	longevity-assurance (LAG1) protein, putative			PF3D7_0508200	longevity-assurance (LAG1) domain protein, putative	ceramide biosynthesis	0	1	1	na	null	null	integral to membrane			
PBANKA_070270	rhomboid protease, putative			PF3D7_0828000	rhomboid protease ROM3 (ROM3)	rhomboid	0	1	1	different in oocyst and sporozoite; (tagged) also different in fertilization and ookinete	null	null	integral to membrane			

PBANKA_111780	conserved Plasmodium protein, unknown function,rhomboid protease ROM10, putative			PF3D7_0618600	rhomboid protease ROM10 (ROM10)	rhomboid	1	0	1	not different from wild type	null	null	integral to membrane
PBANKA_141300	cop-coated vesicle membrane protein p24 precursor, putative			PF3D7_1314500	cop-coated vesicle membrane protein p24 precursor, putative	trafficking	1	0	1	na	null	transport	integral to membrane
PBANKA_031330	MtN3-like protein			PF3D7_0216600	MtN3-like protein	trafficking	1	0	1	na	null	null	integral to membrane
PBANKA_110650	rhomboid protease, putative (ROM4)			PF3D7_0506900	rhomboid protease ROM4 (ROM4)	rhomboid	1	0	1	KO not successful	serine-type endopeptidase activity	null	integral to membrane
PBANKA_131230	SNARE protein, putative			PF3D7_1448600	SNARE protein, putative (VTI1)	trafficking	1	0	1	na	null	vesicle-mediated transport	integral to membrane
PBANKA_133990	SNARE protein, putative			PF3D7_1324700	SNARE protein, putative (YKT6.2)	trafficking	1	0	1	na	null	vesicle-mediated transport	integral to membrane
PBANKA_020700	calcium-transporting ATPase, putative (SERCA)			PF3D7_0106300	calcium-transporting ATPase (ATP6)	transporter	0	1	1	na	ATP binding, calcium-transporting ATPase activity	ATP biosynthetic process, calcium ion transport, metabolic process	integral to membrane, membrane
PBANKA_110310	actin-depolymerizing factor 1 (ADF1)			PF3D7_0503400	actin-depolymerizing factor 1 (ADF1)	gliding motility	0	1	1	KO not successful	actin binding	null	intracellular
PBANKA_131340	exosome complex exonuclease RRP6, putative (RRP6)			PF3D7_1449700	exosome complex exonuclease RRP6, putative (RRP6)	mRNA	0	1	1	na	3'-5' exonuclease activity, nucleic acid binding	nucleobase-containing compound metabolic process	intracellular
PBANKA_100810	GTPase, putative			PF3D7_1436500	conserved protein, unknown function	translation, transporter	0	1	1	na	GTP binding	null	intracellular
PBANKA_145990	signal recognition particle, beta subunit, putative			PF3D7_1246800	signal recognition particle receptor, beta subunit (SRPR-beta)	post-translational modification	0	1	1	na	GTP binding	intracellular protein transport, small GTPase mediated signal transduction	intracellular
PBANKA_132410	ubiquitin carboxyl-terminal hydrolase isozyme L3, putative (UCHL3)			PF3D7_1460400	ubiquitin carboxyl-terminal hydrolase isozyme L3 (UCHL3)	proteasome	0	1	1	na	ubiquitin thiolesterase activity	ubiquitin-dependent protein catabolic process	intracellular
PBANKA_130430	conserved Plasmodium protein, unknown function			PF3D7_1440400	conserved Plasmodium protein, unknown function	unknown function	1	0	1	na	zinc ion binding	null	intracellular
PBANKA_135410	Rab GTPase 11b			PF3D7_1340700	Rab GTPase 11b (RAB11b)	trafficking	1	0	1	na	GTP binding, GTPase activity, protein binding	intracellular protein transport, nucleocytoplasmic transport, small GTPase mediated signal transduction	intracellular
PBANKA_060770	zinc finger transcription factor, putative			PF3D7_1209300	zinc finger transcription factor (KROX1)	transcription	1	0	1	na	zinc ion binding	null	intracellular
PBANKA_100590	zinc-finger, RAN binding protein, putative			PF3D7_0408300	zinc finger, RAN binding protein, putative	transcription	1	0	1	na	zinc ion binding	null	intracellular
PBANKA_050710	CDGSH iron-sulfur domain-containing protein, putative			PF3D7_1022900	CDGSH iron-sulfur domain-containing protein, putative	intracellular Mb-bounded organelle	1	0	1	na	2 iron, 2 sulfur cluster binding	null	intracellular membrane-bounded organelle
PBANKA_140130	40S ribosomal protein S7, putative			PF3D7_1302800	40S ribosomal protein S7, putative	ribosome	0	1	1	na	structural constituent of ribosome	translation	intracellular, ribosome
PBANKA_123390	mitochondrial ribosomal protein L14 precursor, putative	-1.88	0.73	PF3D7_0519100	mitochondrial ribosomal protein L14 precursor, putative	ribosome mito	0	1	1	na	structural constituent of ribosome	translation	intracellular, ribosome
PBANKA_123720	mitochondrial ribosomal protein L17 precursor, putative	-2.23	0.75	PF3D7_0522500	mitochondrial ribosomal protein L17 precursor, putative	ribosome mito	0	1	1	na	structural constituent of ribosome	translation	intracellular, ribosome
PBANKA_145610	40S ribosomal protein S17, putative			PF3D7_1242700	40S ribosomal protein S17, putative	ribosome	1	0	1	na	structural constituent of ribosome	translation	intracellular, ribosome
PBANKA_090640	60S ribosomal protein L35ae, putative			PF3D7_1142600	60S ribosomal protein L35ae, putative	ribosome	1	0	1	na	structural constituent of ribosome	translation	intracellular, ribosome
PBANKA_091810	60S ribosomal protein L38e, putative			PF3D7_1130100	60S ribosomal protein L38e, putative	ribosome	1	0	1	na	structural constituent of ribosome	translation	intracellular, ribosome
PBANKA_135190	60S ribosomal protein L6-2, putative			PF3D7_1338200	60S ribosomal protein L6-2, putative	ribosome	1	0	1	na	structural constituent of ribosome	translation	intracellular, ribosome
PBANKA_081920	organelle ribosomal protein L3 precursor, putative			PF3D7_0918200	organelle ribosomal protein L3 precursor, putative	ribosome	1	0	1	na	structural constituent of ribosome	translation	intracellular, ribosome
PBANKA_120190	40S ribosomal protein S20e, putative			PF3D7_1003500	40S ribosomal protein S20e, putative	ribosome	1	0	1	na	structural constituent of ribosome	translation	intracellular, ribosome, small ribosomal subunit
PBANKA_101500	mitochondrial ribosomal protein L15 precursor, putative	-1.72	0.71	PF3D7_1429700	mitochondrial ribosomal protein L15 precursor, putative	ribosome mito	0	1	1	na	structural constituent of ribosome	translation	large ribosomal subunit
PBANKA_071880	small GTP-binding protein sar1, putative (SAR1)			PF3D7_0416800	small GTP-binding protein sar1 (SAR1)	trafficking	0	1	1	na	GTP binding	intracellular protein transport, small GTPase mediated signal transduction	Maurer's cleft, intracellular
PBANKA_120910	dolichyl-phosphate-mannose protein mannosyltransferase, putative			PF3D7_1010700	dolichyl-phosphate-mannose protein mannosyltransferase, putative	O-glycan biosynthesis	0	1	1	na	null	null	membrane
PBANKA_145500	mitochondrial carrier protein, putative	-2.26	0.75	PF3D7_1241600	mitochondrial carrier protein, putative	transporter	0	1	1	na	binding	transport	membrane
PBANKA_060110	mitochondrial phosphate carrier protein, putative			PF3D7_1202200	mitochondrial phosphate carrier protein (MPC)	transporter	0	1	1	na	binding	transport	membrane

PBANKA_010770	permease, putative	-2.25	0.75	PF3D7_0609100	Zn2 or Fe2 permease	acidocalcisome	0	1	1	na	metal ion transmembrane transporter activity	metal ion transport	membrane
PBANKA_050650	zinc transporter, putative			PF3D7_1022300	zinc transporter, putative	acidocalcisome	0	1	1	na	metal ion transmembrane transporter activity	metal ion transport	membrane
PBANKA_061040	ATPase, putative			PF3D7_1211900	non-SERCA-type Ca2 -transporting P-ATPase (ATP4)	acidocalcisome	1	0	1	na	ATP binding, ATPase activity, coupled to transmembrane movement of ions, phosphorylative mechanism	ATP biosynthetic process, cation transport, metabolic process	membrane
PBANKA_142710	conserved Plasmodium protein, unknown function	-3.76	0.75	PF3D7_0810400	conserved Plasmodium membrane protein, unknown function	unknown function	1	0	1	na	transporter activity	transport	membrane
PBANKA_101380	cytochrome c oxidase subunit II precursor, putative			PF3D7_1430900	cytochrome c oxidase subunit II precursor, putative	redoxmeth	1	0	1	na	copper ion binding, cytochrome-c oxidase activity	null	membrane
PBANKA_093900	ion channel protein, putative			PF3D7_1107900	mechanosensitive ion channel protein	transporter	1	0	1	na	null	null	membrane
PBANKA_141410	phosphatidylinositol synthase, putative (PIS)			PF3D7_1315600	phosphatidylinositol synthase (PIS)	inositol phosphate metabolism	1	0	1	na	phosphotransferase activity, for other substituted phosphate groups	phospholipid biosynthetic process	membrane
PBANKA_131620	SNARE protein, putative			PF3D7_1452500	SNARE protein, putative (PfBet1)	trafficking	1	0	1	na	protein binding	Golgi vesicle transport	membrane
PBANKA_146340	adaptor-related protein complex 3, sigma 2 subunit, putative			PF3D7_1250500	adaptor-related protein complex 3, sigma 2 subunit, putative	trafficking	0	1	1	na	protein binding, protein transporter activity	intracellular protein transport, vesicle-mediated transport	membrane coat
PBANKA_136550	intra-erythrocytic P. berghei-induced structures protein 1 (IBIS1)						1	0	1	different in liver stage	null	null	membrane, symbiont-containing vacuole membrane
PBANKA_082420	perforin like protein 3 (PPLP3)	-2.93	0.75	PF3D7_0923300	perforin like protein 3 (PPLP3)	perforin	0	1	1	different in fertilization, ookinete and oocyst	null	null	microneme
PBANKA_021390	dynein light chain, putative			PF3D7_0729800	dynein light chain, putative	dynein/kinesin	0	1	1	na	microtubule motor activity	microtubule-based process	microtubule associated complex
PBANKA_030220	dynein light chain, putative			PF3D7_0204400	dynein light chain, putative	dynein/kinesin	1	0	1	na	microtubule motor activity	microtubule-based process	microtubule associated complex
PBANKA_083430	conserved Plasmodium protein, unknown function			PF3D7_0933500	conserved Plasmodium protein, unknown function	chromatin	0	1	1	na	null	microtubule cytoskeleton organization	microtubule organizing center, spindle pole
PBANKA_101010	mitochondrial import inner membrane translocase subunit tim17, putative			PF3D7_1434700	mitochondrial import inner membrane translocase subunit tim17, putative	transporter	0	1	1	na	protein transporter activity	protein transport	mitochondrial inner membrane
PBANKA_145630	mitochondrial import inner membrane translocase subunit, putative			PF3D7_1242900	mitochondrial import inner membrane translocase subunit, putative	transporter	0	1	1	na	null	protein import into mitochondrial inner membrane	mitochondrial intermembrane space protein transporter complex
PBANKA_111650	mitochondrial import receptor subunit, putative			PF3D7_0617000	mitochondrial import receptor subunit tom40, putative	transporter	1	0	1	na	voltage-gated anion channel activity	anion transport	mitochondrial outer membrane
PBANKA_142180	mitochondrial ATP synthase F1, epsilon subunit, putative			PF3D7_0715500	mitochondrial ATP synthase F1, epsilon subunit, putative	transporter	1	0	1	na	hydrogen ion transporting ATP synthase activity, rotational mechanism, proton-transporting ATPase activity, rotational mechanism	ATP synthesis coupled proton transport	mitochondrial proton-transporting ATP synthase complex, catalytic core F(1), mitochondrion
PBANKA_041330	mitochondrial ribosomal protein L29/L47 precursor, putative			PF3D7_0315500	mitochondrial ribosomal protein L29/L47 precursor, putative	ribosome mito	1	0	1	na	structural constituent of ribosome	translation	mitochondrial ribosome
PBANKA_092360	GrpE protein homolog, mitochondrial, putative (MGE1)	-2.7	0.75	PF3D7_1124700	co-chaperone GrpE, putative	chaperone, transporter	1	0	1	na	adenyl-nucleotide exchange factor activity, chaperone binding, protein homodimerization activity	protein folding, protein import into mitochondrial matrix	mitochondrion
PBANKA_102500	conserved Plasmodium protein, unknown function			PF3D7_1417700	conserved Plasmodium protein, unknown function	unknown function	0	1	1	na	null	null	nuclear periphery, nucleus
PBANKA_111700	histone H2A, putative (H2A)			PF3D7_0617800	histone H2A (H2A)	chaperone, chromatin	0	1	1	na	DNA binding	nucleosome assembly	nucleosome, nucleus
PBANKA_142060	histone H2B, putative			PF3D7_0714000	histone H2B variant, putative (H2Bv)	chaperone	1	0	1	(tagged) different in liver stage	DNA binding	nucleosome assembly	nucleosome, nucleus
PBANKA_143940	chromatin assembly protein, putative			PF3D7_1224500	chromatin assembly protein (ASF1), putative	chromatin	0	1	1	na	null	chromatin assembly or disassembly	nucleus
PBANKA_142770	RuvB DNA helicase, putative			PF3D7_0809700	RuvB DNA helicase, putative	DNA replication	0	1	1	na	ATP binding, ATP-dependent 5'-3' DNA helicase activity, ATPase activity, DNA helicase activity	null	nucleus
PBANKA_113440	conserved Plasmodium protein, unknown function			PF3D7_1358100	small subunit rRNA processing factor, putative	RNA	1	0	1	na	null	gene silencing	nucleus
PBANKA_113090	methyltransferase, putative	-2.13	0.74	PF3D7_1354300	large subunit rRNA methyltransferase, putative	RNA	1	0	1	na	methyltransferase activity, nucleic acid binding	rRNA methylation	nucleus
PBANKA_051140	peroxiredoxin, putative (nPrx, UIS16)			PF3D7_1027300	peroxiredoxin (nPrx)	redoxmeth	1	0	1	na	antioxidant activity, oxidoreductase activity	null	nucleus
PBANKA_090720	phd finger protein, putative			PF3D7_1141800	phd finger protein, putative	chromatin	1	0	1	na	protein binding, zinc ion binding	null	nucleus
PBANKA_111910	splicing factor 3A subunit 2, putative (SF3A2)			PF3D7_0619900	splicing factor 3A subunit 2, putative (SF3A2)	mRNA	1	0	1	na	nucleic acid binding, zinc ion binding	null	nucleus
PBANKA_133200	U2 snRNP auxiliary factor, putative			PF3D7_1468800	U2 snRNP auxiliary factor, putative	mRNA	1	0	1	na	RNA binding	mRNA processing	nucleus

PBANKA_132740	yl1 nuclear protein, putative			PF3D7_1464000	YL1 nuclear protein, putative	transcription	1	0	1	na	sequence-specific DNA binding transcription factor activity	regulation of transcription, DNA-dependent	nucleus			
PBANKA_020290	transcription initiation factor TFIIB, putative			PF3D7_0110800	transcription initiation factor TFIIB, putative	transcription	1	0	1	na	protein binding, translation initiation factor activity, zinc ion binding	DNA-dependent transcription, initiation, regulation of transcription, DNA-dependent, translational initiation	nucleus, transcription factor complex			
PBANKA_110580	TATA-box binding protein, putative,transcription initiation factor tfiid, putative (TBP)			PF3D7_0506200	transcription initiation factor TFiid, TATA-binding protein (TBP)	transcription	0	1	1	na	DNA binding	regulation of transcription, DNA-dependent, transcription initiation from RNA polymerase II promoter	nucleus, transcription factor TFIID complex			
PBANKA_101590	transcription initiation TFIID-like, putative			PF3D7_1428800	transcription initiation TFIID-like, putative	transcription	0	1	1	na	DNA binding	regulation of transcription, DNA-dependent, transcription initiation from RNA polymerase II promoter	nucleus, transcription factor TFIID complex			
PBANKA_114400	26s proteasome regulatory subunit rpn11, putative (RPN11)			PF3D7_1368100	proteasome regulatory subunit, putative	proteasome	0	1	1	na	null	null	null	null	ubiquitin-dependent protein catabolic process	proteasome regulatory particle
PBANKA_112620	4-methyl-5(B-hydroxyethyl)-thiazol monophosphate biosynthesis enzyme, putative			PF3D7_0627500	4-methyl-5(B-hydroxyethyl)-thiazol monophosphate biosynthesis enzyme	thiamine (vitB1) metabolism	0	1	1	na	null	null	null	null	thiamine biosynthetic process	null
PBANKA_130550	acid cluster protein 33 homologue, putative	-2.34	0.75	PF3D7_1441600	acid cluster protein 33 homologue, putative		0	1	1	na	null	null	null	null	null	null
PBANKA_112340	anaphase-promoting complex subunit, putative			PF3D7_0624500	anaphase promoting complex subunit, putative	meiosis	0	1	1	na	protein binding, zinc ion binding	null	null			
PBANKA_031500	apicoplast RNA methyltransferase precursor, putative			PF3D7_0218300	apicoplast RNA methyltransferase precursor, putative	RNA	0	1	1	na	RNA binding, RNA methyltransferase activity	RNA processing	null			
PBANKA_123260	apicortin, putative	-1.89	0.7	PF3D7_0517800	apicortin, putative	apicoplast	0	1	1	na	null	intracellular signal transduction	null			
PBANKA_135770	aspartate carbamoyltransferase, putative	-4.56	0.75	PF3D7_1344800	aspartate carbamoyltransferase (atcasE)	pyrimidine metabolism	0	1	1	na	amino acid binding, aspartate carbamoyltransferase activity	'de novo' pyrimidine nucleobase biosynthetic process, cellular amino acid metabolic process	null			
PBANKA_140020	BIR protein					BIR protein	0	1	1	na	null	null	null	na	na	na
PBANKA_031640	BIR protein, pseudogene					BIR protein	0	1	1	na	null	null	null			
PBANKA_050080	BIR protein, pseudogene					BIR protein	0	1	1	na	null	null	null	na	na	na
PBANKA_040820	calcium dependent protein kinase 3 (CDPK3)	-1.68	0.71	PF3D7_0310100	calcium dependent protein kinase 3 (CDPK3)	kinase	0	1	1	different in fertilization, ookinete and oocyst	ATP binding, calcium ion binding, protein serine/threonine kinase activity, protein tyrosine kinase activity	protein phosphorylation	null			
PBANKA_101980	calcium-dependent protein kinase, putative	-6.75	0.75	PF3D7_1423600	serine/threonine protein kinase, putative, cdlk	kinase	0	1	1	different in oocyst, sporozoite and liver stage	ATP binding, protein serine/threonine kinase activity, protein tyrosine kinase activity	protein phosphorylation	null			
PBANKA_091210	casein kinase 1 (CK1)			PF3D7_1136500.1/2	casein kinase 1 (CK1)	kinase	0	1	1	KO not successful	ATP binding, protein serine/threonine kinase activity, protein tyrosine kinase activity	protein phosphorylation	null			
PBANKA_051910	CCAT-binding transcription factor-like protein, putative			PF3D7_1036600.1/2	conserved Plasmodium protein, unknown function	transcription	0	1	1	na	null	null	null	na	na	na
PBANKA_101600	conserved Plasmodium protein, unknown function	-2.1	0.73	PF3D7_1428700	conserved protein, unknown function	redoxmeth	0	1	1	na	heme binding	null	null			
PBANKA_060600	conserved Plasmodium protein, unknown function			PF3D7_1207500	conserved Plasmodium protein, unknown function	unknown function	0	1	1	na	nucleic acid binding	null	null			
PBANKA_132780	conserved Plasmodium protein, unknown function			PF3D7_1464400	zinc finger protein, putative	nucleic acid binding	0	1	1	na	nucleic acid binding, zinc ion binding	null	null			
PBANKA_010510	conserved Plasmodium protein, unknown function			PF3D7_0606400	conserved Plasmodium protein, unknown function	unknown function	0	1	1	na	null	null	null	null	null	cytoplasm, membrane
PBANKA_010700	conserved Plasmodium protein, unknown function			PF3D7_0608400	conserved Plasmodium protein, unknown function	unknown function	0	1	1	na	null	null	null	null	null	null
PBANKA_010960	conserved Plasmodium protein, unknown function			PF3D7_0611300	conserved Plasmodium protein, unknown function	unknown function	0	1	1	na	null	null	null	null	null	null
PBANKA_010990	conserved Plasmodium protein, unknown function	-3.42	0.74	PF3D7_0611600	conserved Plasmodium protein, unknown function	unknown function	0	1	1	na	null	null	null	null	null	null
PBANKA_020790	conserved Plasmodium protein, unknown function			PF3D7_0105400.1/2	conserved Plasmodium protein, unknown function	unknown function	0	1	1	na	null	null	null	null	null	integral to membrane, membrane
PBANKA_021160	conserved Plasmodium protein, unknown function			PF3D7_0727500	conserved Plasmodium protein, unknown function	mitochondrial transcription	0	1	1	na	null	null	null	null	null	apicoplast
PBANKA_021260	conserved Plasmodium protein, unknown function			PF3D7_0728500	conserved Plasmodium protein, unknown function	kinase	0	1	1	na	null	null	null	null	null	null
PBANKA_021450	conserved Plasmodium protein, unknown function			PF3D7_0730400	conserved Plasmodium protein, unknown function	unknown function	0	1	1	na	null	null	null	null	null	null
PBANKA_030280	conserved Plasmodium protein, unknown function			PF3D7_0205100	conserved Plasmodium protein, unknown function	DNA repair	0	1	1	na	null	null	null	protein binding	null	null
PBANKA_030350	conserved Plasmodium protein, unknown function	-2.1	0.73	PF3D7_0205800	conserved Plasmodium protein, unknown function	phospholipid binding	0	1	1	na	null	null	null	null	null	null

PBANKA_040880	conserved Plasmodium protein, unknown function			PF3D7_0310800	conserved Plasmodium protein, unknown function	unknown function	0	1	1	na	null	null	null	null	null	apicoplast, integral to membrane, membrane
PBANKA_041140	conserved Plasmodium protein, unknown function			PF3D7_0313700.1/2	conserved Plasmodium protein, unknown function	unknown function	0	1	1	na	null	null	null	null	null	null
PBANKA_041560	conserved Plasmodium protein, unknown function			PF3D7_0905800	conserved Plasmodium protein, unknown function	unknown function	0	1	1	na	null	null	null	DNA binding, protein binding, translation elongation factor activity, zinc ion binding	DNA-dependent transcription, elongation, regulation of transcription from RNA polymerase II promoter, transcription, DNA-dependent	nucleus
PBANKA_050780	conserved Plasmodium protein, unknown function			PF3D7_1023600	conserved Plasmodium protein, unknown function	unknown function	0	1	1	na	null	null	null	ATP binding, motor activity, protein binding	null	cytoplasm
PBANKA_051600	conserved Plasmodium protein, unknown function			PF3D7_1032200	conserved Plasmodium protein, unknown function	unknown function	0	1	1	na	null	null	null	null	null	null
PBANKA_052200	conserved Plasmodium protein, unknown function			PF3D7_0420600	conserved Plasmodium protein, unknown function	unknown function	0	1	1	na	null	null	null	null	null	null
PBANKA_062020	conserved Plasmodium protein, unknown function			PF3D7_0722700	conserved Plasmodium protein, unknown function	unknown function	0	1	1	na	null	null	null	null	null	integral to membrane
PBANKA_071300	conserved Plasmodium protein, unknown function			PF3D7_0817800	conserved Plasmodium protein, unknown function	unknown function	0	1	1	na	null	null	null	metal ion binding, nucleotide binding	null	null
PBANKA_080140	conserved Plasmodium protein, unknown function			PF3D7_0703700	conserved Plasmodium protein, unknown function	unknown function	0	1	1	na	null	null	null	null	null	null
PBANKA_081510	conserved Plasmodium protein, unknown function	-3.06	0.75	PF3D7_0914100	conserved Plasmodium protein, unknown function	unknown function	0	1	1	na	null	null	null	ATP binding, motor activity, protein binding	null	cytoplasm
PBANKA_082930	conserved Plasmodium protein, unknown function			PF3D7_0928500	conserved Plasmodium protein, unknown function	unknown function	0	1	1	na	null	null	null	protein binding	null	null
PBANKA_092250	conserved Plasmodium protein, unknown function			PF3D7_1125800	kelch protein, putative	microtubule	0	1	1	na	null	null	null	null	null	null
PBANKA_092270	conserved Plasmodium protein, unknown function			PF3D7_1125600	conserved Plasmodium protein, unknown function	unknown function	0	1	1	na	null	null	null	null	null	null
PBANKA_092330	conserved Plasmodium protein, unknown function	-3.36	0.75	PF3D7_1125000	conserved Plasmodium protein, unknown function	unknown function	0	1	1	na	null	null	null	ATP binding, ATPase activity	transport	apicoplast, plasma membrane
PBANKA_092760	conserved Plasmodium protein, unknown function			PF3D7_1120700	conserved Plasmodium protein, unknown function	unknown function	0	1	1	na	null	null	null	null	null	integral to membrane, membrane, nucleus
PBANKA_092820	conserved Plasmodium protein, unknown function			PF3D7_1120000	conserved Plasmodium protein, unknown function	unknown function	0	1	1	na	null	null	null	ATP binding	null	cytoplasm
PBANKA_093060	conserved Plasmodium protein, unknown function			PF3D7_1117400	conserved Plasmodium protein, unknown function	unknown function	0	1	1	na	null	null	null	null	null	membrane
PBANKA_100320	conserved Plasmodium protein, unknown function			PF3D7_0405500	conserved Plasmodium protein, unknown function	unknown function	0	1	1	na	null	null	null	null	null	null
PBANKA_102600	conserved Plasmodium protein, unknown function			PF3D7_1416700	conserved Plasmodium protein, unknown function	unknown function	0	1	1	na	null	null	null	null	null	cytoplasm
PBANKA_103140	conserved Plasmodium protein, unknown function			PF3D7_1411100.1/2	conserved Plasmodium membrane protein, unknown function	unknown function	0	1	1	na	null	null	null	null	null	integral to membrane, plasma membrane
PBANKA_110750	conserved Plasmodium protein, unknown function			PF3D7_0507900	conserved Plasmodium protein, unknown function	unknown function	0	1	1	na	null	null	null	null	transport	integral to membrane, plasma membrane
PBANKA_111280	conserved Plasmodium protein, unknown function	-2.43	0.75	PF3D7_0513100	conserved Plasmodium protein, unknown function	unknown function	0	1	1	na	null	null	null	null	null	cytoplasm
PBANKA_111410	conserved Plasmodium protein, unknown function	-4.23	0.75	PF3D7_0514500	conserved Plasmodium membrane protein, unknown function	unknown function	0	1	1	na	null	null	null	null	null	membrane
PBANKA_111930	conserved Plasmodium protein, unknown function			PF3D7_0620100	conserved Plasmodium protein, unknown function	unknown function	0	1	1	na	null	null	null	null	null	cytoplasm
PBANKA_120530	conserved Plasmodium protein, unknown function			PF3D7_1007100	conserved Plasmodium protein, unknown function	unknown function	0	1	1	na	null	null	null	null	null	null
PBANKA_122170	conserved Plasmodium protein, unknown function			PF3D7_0711100	conserved Plasmodium membrane protein, unknown function	unknown function	0	1	1	na	null	null	null	null	null	null
PBANKA_123370	conserved Plasmodium protein, unknown function			PF3D7_0518900	conserved Plasmodium protein, unknown function	unknown function	0	1	1	na	null	null	null	null	null	cytoplasm
PBANKA_123470	conserved Plasmodium protein, unknown function			PF3D7_0519900	conserved Plasmodium protein, unknown function	unknown function	0	1	1	na	null	null	null	null	null	apicoplast
PBANKA_123810	conserved Plasmodium protein, unknown function			PF3D7_0523300	conserved Plasmodium protein, unknown function	unknown function	0	1	1	na	null	null	null	null	null	null
PBANKA_123840	conserved Plasmodium protein, unknown function			PF3D7_0523600	conserved Plasmodium protein, unknown function	unknown function	0	1	1	na	null	null	null	null	null	cytoplasm
PBANKA_123870	conserved Plasmodium protein, unknown function			PF3D7_0523900	conserved Plasmodium membrane protein, unknown function	unknown function	0	1	1	na	null	null	null	G-protein coupled receptor activity	G-protein coupled receptor signaling pathway	integral to membrane, membrane

PBANKA_131290	conserved Plasmodium protein, unknown function					unknown function	0	1	1	na	null	null	null	na	na	na
PBANKA_131610	conserved Plasmodium protein, unknown function			PF3D7_1452400	conserved Plasmodium protein, unknown function	unknown function	0	1	1	na	null	null	null	null	null	cytoplasm, nucleus
PBANKA_131940	conserved Plasmodium protein, unknown function			PF3D7_1455700	conserved Plasmodium protein, unknown function	unknown function	0	1	1	na	null	null	null	null	null	cytoplasm
PBANKA_132190	conserved Plasmodium protein, unknown function			PF3D7_1458200	conserved Plasmodium protein, unknown function	unknown function	0	1	1	na	null	null	null	null	null	null
PBANKA_132240	conserved Plasmodium protein, unknown function			PF3D7_1458700	conserved Plasmodium protein, unknown function	unknown function	0	1	1	na	null	null	null	null	regulation of cell shape	mitochondrion
PBANKA_132880	conserved Plasmodium protein, unknown function			PF3D7_1465400	conserved Plasmodium protein, unknown function	unknown function	0	1	1	na	null	null	null	null	null	null
PBANKA_133910	conserved Plasmodium protein, unknown function			PF3D7_1323900	conserved Plasmodium protein, unknown function	Ca2+ binding, prot-prot interaction	0	1	1	na	null	null	null	null	protein transport	cytoplasm, nucleus
PBANKA_134110	conserved Plasmodium protein, unknown function			PF3D7_1326000	conserved Plasmodium protein, unknown function	unknown function	0	1	1	na	null	null	null	null	null	null
PBANKA_134420	conserved Plasmodium protein, unknown function			PF3D7_1329200	conserved Plasmodium protein, unknown function	unknown function	0	1	1	na	null	null	null	nucleotide binding	null	cytoplasm
PBANKA_135170	conserved Plasmodium protein, unknown function			PF3D7_1338000	conserved Plasmodium membrane protein, unknown function	unknown function	0	1	1	na	null	null	null	null	null	integral to membrane, membrane
PBANKA_136020	conserved Plasmodium protein, unknown function	-2.25	0.74	PF3D7_1347400	conserved Plasmodium protein, unknown function	unknown function	0	1	1	na	null	null	null	null	null	integral to membrane, plasma membrane
PBANKA_136360	conserved Plasmodium protein, unknown function			PF3D7_1350800	conserved Plasmodium protein, unknown function	unknown function	0	1	1	na	null	null	null	null	null	null
PBANKA_140290	conserved Plasmodium protein, unknown function			PF3D7_1304400	conserved Plasmodium protein, unknown function	unknown function	0	1	1	na	null	null	null	null	null	null
PBANKA_141010	conserved Plasmodium protein, unknown function	-6.66	0.75	PF3D7_1311600	conserved Plasmodium protein, unknown function	unknown function	0	1	1	na	null	null	null	null	null	membrane
PBANKA_141350	conserved Plasmodium protein, unknown function			PF3D7_1315000	conserved Plasmodium protein, unknown function	unknown function	0	1	1	na	null	null	null	null	null	integral to membrane, membrane
PBANKA_142920	conserved Plasmodium protein, unknown function	-3.32	0.75	PF3D7_1213400	conserved Plasmodium protein, unknown function	galactose metabolism	0	1	1	na	null	null	null	protein binding	null	cytoplasm, nucleus
PBANKA_143060	conserved Plasmodium protein, unknown function			PF3D7_1214800	conserved Plasmodium protein, unknown function	unknown function	0	1	1	na	null	null	null	null	null	apicoplast
PBANKA_143580	conserved Plasmodium protein, unknown function	-1.96	0.73	PF3D7_1220600	conserved Plasmodium protein, unknown function	unknown function	0	1	1	na	null	null	null	null	null	apicoplast, membrane
PBANKA_144780	conserved Plasmodium protein, unknown function			PF3D7_1233100	conserved protein, unknown function	unknown function	0	1	1	KO not successful	null	null	null	null	null	null
PBANKA_145220	conserved Plasmodium protein, unknown function			PF3D7_1237600	rRNA processing WD-repeat protein, putative	RNA	0	1	1	na	null	null	null	null	null	cytoplasm, nucleus
PBANKA_145550	conserved Plasmodium protein, unknown function			PF3D7_1242100	conserved Plasmodium protein, unknown function	unknown function	0	1	1	na	null	null	null	null	null	null
PBANKA_061530	conserved Plasmodium protein, unknown function	-3.55	0.75	PF3D7_0717600	conserved Plasmodium protein, unknown function	unknown function	1	0	1	na	binding	null	null			
PBANKA_101870	conserved Plasmodium protein, unknown function			PF3D7_1425900	conserved Plasmodium protein, unknown function	transcription	1	0	1	na	binding	null	null			
PBANKA_103890	conserved Plasmodium protein, unknown function			PF3D7_1403100	conserved Plasmodium protein, unknown function	chromatin	1	0	1	na	binding	null	null			
PBANKA_134700	conserved Plasmodium protein, unknown function	-1.99	0.72	PF3D7_1332200	conserved Plasmodium protein, unknown function	unknown function	1	0	1	na	DNA binding, alkylbase DNA N-glycosylase activity	base-excision repair	null			
PBANKA_131310	conserved Plasmodium protein, unknown function	-3.93	0.75	PF3D7_1449400	DNA replication related protein, putative	DNA replication	1	0	1	na	DNA binding, nuclease activity, protein binding	DNA metabolic process	null			
PBANKA_146240	cutA, putative			PF3D7_1249500	cutA, putative	protein binding, response to ion	0	1	1	na	null	response to metal ion	null			
PBANKA_021130	cysteine desulfurase, putative (NFS)			PF3D7_0727200	cysteine desulfurase, putative (NFS)	thiamine (vitB1) metabolism	0	1	1	na	null	metabolic process	null			
PBANKA_146070	dipeptidyl peptidase 2, putative (DPAP2)			PF3D7_1247800	dipeptidyl peptidase 2, putative (DPAP2)	post-translational modification	0	1	1	na	cysteine-type peptidase activity	proteolysis	null			
PBANKA_092300	DNA-dependent RNA polymerase, putative			PF3D7_1125300	DNA-dependent RNA polymerase	transcription	0	1	1	na	DNA binding, DNA-directed RNA polymerase activity	transcription, DNA-dependent	null			
PBANKA_040720	dual-specificity protein phosphatase, putative (YVH1)			PF3D7_0309000	dual specificity phosphatase (YVH1)	phosphatase	0	1	1	na	protein tyrosine/serine/threonine phosphatase activity	protein dephosphorylation	null			

PBANKA_052420	early transcribed membrane protein (SEP2)					early transcribed membrane protein (ETRAMP)	0	1	1	KO not successful; (tagged) different in fertilization, ookinete, oocyst, sporozoite and liver stage	null	null	null	null	null	null
PBANKA_103190	eukaryotic translation initiation factor 2 gamma subunit, putative			PF3D7_1410600	eukaryotic translation initiation factor 2 gamma subunit, putative	translation	0	1	1	na	GTP binding, GTPase activity	null	null			
PBANKA_133430	exported protein 2, putative (EXP2)			PF3D7_1471100	exported protein 2 (EXP2)	trafficking	0	1	1	(tagged) different in sporozoite and liver stage	null	null	null	null	null	symbiont-containing vacuole membrane
PBANKA_124710	fam-b protein					unknown function	0	1	1	na	null	null	null	na	na	na
PBANKA_131070	glutaminyl-peptide cyclotransferase, putative	-2.08	0.74	PF3D7_1446900	glutaminyl-peptide cyclotransferase, putative		0	1	1	na	null	null	null	null	null	apicoplast, membrane
PBANKA_130970	helicase, putative			PF3D7_1445900	DEAD/DEAH box ATP-dependent RNA helicase, putative	mRNA	0	1	1	na	ATP binding, ATP-dependent helicase activity, helicase activity, nucleic acid binding	null	null			
PBANKA_071840	histone acetyltransferase, putative (HAT1)			PF3D7_0416400	histone acetyltransferase, putative (HAT1)	chromatin modifying proteins	0	1	1	na	histone acetyltransferase activity	chromatin modification	null			
PBANKA_136440	inner membrane complex protein 1f, putative (IMC1F, ALV6)	-2.42	0.73	PF3D7_1351700	alveolin, putative (ALV6)	gliding motility	0	1	1	na	null	null	null	transferase activity	null	inner membrane complex
PBANKA_093890	IWS1-like protein, putative			PF3D7_1108000	IWS1-like protein, putative	transcription	0	1	1	na	null	null	null	null	null	null
PBANKA_050420	large subunit rRNA processing RRM protein, putative			PF3D7_1020000	large subunit rRNA processing RRM protein, putative	RNA	0	1	1	na	nucleic acid binding	null	null			
PBANKA_030440	merozoite surface protein 4/5 (MSP4/5)			PF3D7_0207000	merozoite surface protein 4 (MSP4)	contaminant	0	1	1	na	null	null	null	null	null	integral to membrane
PBANKA_010300	mitochondrial ribosomal protein L41 precursor, putative			PF3D7_0604200	mitochondrial ribosomal protein L41 precursor, putative	ribosome mito	0	1	1	na	null	null	null	null	null	null
PBANKA_131830	mitochondrial ribosomal protein S11 precursor, putative			PF3D7_1454600	mitochondrial ribosomal protein S11 precursor, putative	ribosome mito	0	1	1	na	null	null	null	structural constituent of ribosome	translation	mitochondrion, organellar small ribosomal subunit
PBANKA_122690	mitogen-activated protein kinase organizer 1, putative			PF3D7_0803300	mitogen-activated protein kinase organizer 1, putative (PfMORG1)	kinase	0	1	1	na	null	null	null	null	null	null
PBANKA_030720	monocarboxylate transporter, putative			PF3D7_0210300	monocarboxylate transporter, putative	glycolysis	0	1	1	na	null	null	null	monocarboxylic acid transmembrane transporter activity	monocarboxylic acid transport	membrane
PBANKA_020410	N-terminal acetyltransferase, putative			PF3D7_0109500	N-acetyltransferase, putative	chromatin modifying proteins	0	1	1	na	N-acetyltransferase activity	metabolic process	null			
PBANKA_135060	nuclear movement protein, putative			PF3D7_1336800	nuclear movement protein, putative	microtubule	0	1	1	na	null	null	null	null	null	null
PBANKA_041630	nucleoporin NUP100/NSP100, putative	-1.63	0.7	PF3D7_0905100	nucleoporin NUP100/NSP100, putative (NUP100)	RNA	0	1	1	na	null	null	null	null	null	nuclear pore, nucleus
PBANKA_010740	ornithine aminotransferase, putative (OAT)			PF3D7_0608800	ornithine aminotransferase (OAT)	AA metabolism	0	1	1	na	pyridoxal phosphate binding, transaminase activity	null	null			
PBANKA_093170	peptidyl-prolyl cis-trans isomerase, putative (CYP19C)			PF3D7_1116300	peptidyl-prolyl cis-trans isomerase (CYP19C)	chaperone	0	1	1	na	peptidyl-prolyl cis-trans isomerase activity	null	null			
PBANKA_051620	phosphatidyl inositol glycan, class A, putative			PF3D7_1032400	N-acetylglucosaminyl-phosphatidylinositol biosynthetic protein, putative	GPI	0	1	1	na	null	GPI anchor biosynthetic process, biosynthetic process	null			
PBANKA_112810	phospholipase (PL)			PF3D7_0629300	phosphatidylcholine-sterol acyltransferase precursor, putative (PL)	utilization phospholipids	0	1	1	different in sporozoite and liver stage	phosphatidylcholine-sterol O-acyltransferase activity	lipid metabolic process	null			
PBANKA_020460	photosensitized INA-labeled protein 1, putative			PF3D7_0109000	photosensitized INA-labeled protein 1, PhIL1, putative	gliding motility	0	1	1	na	null	null	null	null	null	null
PBANKA_122250	plasmepsin X			PF3D7_0808200	plasmepsin X	plasmepsin, protease	0	1	1	KO not successful	aspartic-type endopeptidase activity	proteolysis	null			
PBANKA_000110	Plasmodium exported protein, unknown function						0	1	1	na	null	null	null			
PBANKA_011090	PP-loop family protein, putative			PF3D7_0612600	PP-loop family protein, putative	translation	0	1	1	na	ATP binding	tRNA processing	null			
PBANKA_111420	ribose 5-phosphate epimerase, putative	-4.29	0.75	PF3D7_0514600	ribose 5-phosphate epimerase, putative	glycolysis	0	1	1	na	ribose-5-phosphate isomerase activity	pentose-phosphate shunt, non-oxidative branch	null			
PBANKA_113630	RNA-binding protein, putative	-1.66	0.71	PF3D7_1360100	RNA binding protein, putative	mRNA	0	1	1	na	nucleic acid binding	null	null			
PBANKA_051240	rrna-processing protein ebp2, putative (EBP2)			PF3D7_1028300	nucleolar preribosomal assembly protein, putative	ribosome assembly	0	1	1	na	null	null	null	null	null	null
PBANKA_040780	SECIS-binding protein 2, putative (SBP2)			PF3D7_0309700	SECIS-binding protein 2, putative (SBP2); ribosomal protein l7ae, putative	ribosome	0	1	1	na	null	null	null	null	null	null
PBANKA_103780	secreted ookinete adhesive protein (SOAP)	-3.26	0.75	PF3D7_1404300	secreted ookinete adhesive protein (SOAP)	adhesin	0	1	1	different in fertilization, ookinete, oocyst, sporozoite and liver stage	null	null	null	null	null	apicoplast

PBANKA_122200	sin3 associated polypeptide p18 protein, putative			PF3D7_0711400	sin3 associated polypeptide p18-like protein	chromatin	0	1	1	na	null	null	null	transcription corepressor activity	regulation of transcription from RNA polymerase II promoter	histone deacetylase complex
PBANKA_141880	SNARE protein, putative			PF3D7_1320500	SNARE protein, putative (SNAP23)	trafficking	0	1	1	na	null	null	null	SNAP receptor activity, protein binding	intracellular protein transport, regulation of vesicle fusion, vesicle-mediated transport	SNARE complex
PBANKA_134980	sporozoite surface protein 2 (SSP2),thrombospondin-related anonymous protein (TRAP), S8			PF3D7_1335900	sporozoite surface protein 2 (TRAP)	gliding motility	0	1	1	different in sporozoite and liver stage	null	null	null	null	cellular component movement, entry into host	cytoplasm, membrane, microneme
PBANKA_144500	subpellicular microtubule protein 2, putative (SPM2)	-6.63	0.75	PF3D7_1230300	subpellicular microtubule protein 2, putative (SPM2)	gliding motility	0	1	1	na	null	null	null	transferase activity	null	null
PBANKA_112980	Tetratricopeptide repeat protein, putative	-2.65	0.75	PF3D7_0631000	Tetratricopeptide repeat protein, putative	chaperone	0	1	1	not different from wild type	null	null	null	null	null	null
PBANKA_093230	transcription factor with AP2 domain(s), putative (ApiAP2)			PF3D7_1115500	transcription factor with AP2 domain(s), putative (ApiAP2)	transcription	0	1	1	na	sequence-specific DNA binding transcription factor activity	regulation of transcription, DNA-dependent	null			
PBANKA_142390	translation initiation factor SUI1, putative			PF3D7_0813600	translation initiation factor SUI1, putative	translation	0	1	1	na	translation initiation factor activity	translational initiation	null			
PBANKA_130380	triose-phosphate isomerase, putative (TIM)			PF3D7_1439900	triosephosphate isomerase (TIM)	glycolysis	0	1	1	na	triose-phosphate isomerase activity	metabolic process	null			
PBANKA_062170	type 2A phosphatase-associated protein 42, putative (TAP42)			PF3D7_0724200	immunoglobulin-binding protein 1-related, putative		0	1	1	na	null	regulation of signal transduction, response to biotic stimulus	null			
PBANKA_114570	U2 small nuclear ribonucleoprotein A, putative			PF3D7_1369700	U1 small nuclear ribonucleoprotein a, putative	mRNA	0	1	1	na	protein binding	null	null			
PBANKA_062010	U3 snoRNA-associated small subunit rRNA processing protein, putative			PF3D7_0722600	nucleolar rRNA processing protein, putative	RNA	0	1	1	na	null	null	null	null	null	null
PBANKA_082190	ubiquitin conjugating enzyme, putative			PF3D7_0921000.1	ubiquitin conjugating enzyme, putative	proteasome	0	1	1	na	small conjugating protein ligase activity	post-translational protein modification, regulation of protein metabolic process	null			
PBANKA_080600	ubiquitin-conjugating enzyme, putative			PF3D7_0319300	ubiquitin conjugating enzyme, putative	proteasome	0	1	1	na	small conjugating protein ligase activity	post-translational protein modification, regulation of protein metabolic process	null			
PBANKA_051770	ubiquitin-conjugating enzyme, putative			PF3D7_1033900	ubiquitin conjugating enzyme, putative	proteasome	0	1	1	na	ubiquitin-protein ligase activity	post-translational protein modification, protein ubiquitination, regulation of protein metabolic process	null			
PBANKA_141390	zinc finger (CCCH type) protein, putative			PF3D7_1315400	zinc finger (CCCH type) protein, putative	transcription	0	1	1	na	null	null	null	null	null	null
PBANKA_090950	AAA family ATPase, putative			PF3D7_1139500.1/2	AAA family ATPase, putative	transporter	1	0	1	na	ATP binding, nucleoside-triphosphatase activity	null	null			
PBANKA_051980	acetyl-CoA transporter, putative			PF3D7_1036800	acetyl-CoA transporter, putative	TCA	1	0	1	na	null	null	null	null	null	membrane
PBANKA_123990	acyl-CoA synthetase, putative			PF3D7_0525100	acyl-CoA synthetase (ACS10)	lipid metabolism	1	0	1	na	catalytic activity	metabolic process	null			
PBANKA_123560	adenosylhomocysteinase, putative (SAHH)			PF3D7_0520900	S-adenosyl-L-homocysteine hydrolase (SAHH)	AA metabolism	1	0	1	na	adenosylhomocysteinase activity, binding	one-carbon metabolic process	null			
PBANKA_103240	alpha/beta hydrolase, putative			PF3D7_1410100	alpha/beta hydrolase, putative	protease	1	0	1	na	null	null	null	null	null	membrane
PBANKA_123330	ATP-dependent RNA helicase DDX23, putative (DDX23)			PF3D7_0518500	ATP-dependent RNA helicase DDX23, putative (DDX23)	mRNA	1	0	1	na	ATP binding, ATP-dependent helicase activity, helicase activity, nucleic acid binding	null	null			
PBANKA_010050	BIR protein					BIR protein	1	0	1	na	null	null	null			
PBANKA_060030	BIR protein					BIR protein	1	0	1	na	null	null	null	null	null	integral to membrane
PBANKA_146530	BIR protein, pseudogene					BIR protein	1	0	1	na	null	null	null			
PBANKA_145260	calcyclin binding protein, putative			PF3D7_1238100	calcyclin binding protein, putative	proteasome	1	0	1	na	null	null	null	null	null	membrane
PBANKA_094140	caltractin (centrin), putative (CEN4)			PF3D7_1105500	centrin-4 (CEN4)	chromatin	1	0	1	na	calcium ion binding	null	null			
PBANKA_123430	carbon catabolite repressor protein 4, putative (CCR4)			PF3D7_0519500	carbon catabolite repressor protein 4, putative (CCR4)	mRNA	1	0	1	na	null	null	null	null	null	null
PBANKA_134430	chromatin assembly factor 1 subunit, putative			PF3D7_1329300	chromatin assembly factor 1 subunit, putative	chromatin	1	0	1	na	null	null	null	chromatin binding, histone binding, unfolded protein binding	chromatin assembly or disassembly	chromatin assembly complex
PBANKA_111460	conserved Plasmodium protein, unknown function			PF3D7_0515000	RNA recognition motif, putative	mRNA	1	0	1	na	nucleic acid binding	null	null			
PBANKA_010150	conserved Plasmodium protein, unknown function	-4.87	0.75	PF3D7_0602700	conserved Plasmodium protein, unknown function	unknown function	1	0	1	na	null	null	null	null	null	null
PBANKA_010180	conserved Plasmodium protein, unknown function	-2.17	0.73	PF3D7_0603000	conserved Plasmodium protein, unknown function	unknown function	1	0	1	na	null	null	null	null	null	cytoplasm, membrane

PBANKA_010190	conserved Plasmodium protein, unknown function			PF3D7_0603100	conserved Plasmodium protein, unknown function	unknown function	1	0	1	na	null	null	null	nucleic acid binding	null	nucleus
PBANKA_010400	conserved Plasmodium protein, unknown function			PF3D7_0605200	conserved Plasmodium protein, unknown function	unknown function	1	0	1	na	null	null	null	acylphosphatase activity	null	null
PBANKA_011060	conserved Plasmodium protein, unknown function			PF3D7_0612300	conserved Plasmodium protein, unknown function	unknown function	1	0	1	na	null	null	null	null	null	integral to membrane, membrane
PBANKA_011080	conserved Plasmodium protein, unknown function			PF3D7_0612500	conserved Plasmodium protein, unknown function	unknown function	1	0	1	na	null	null	null	null	null	null
PBANKA_011140	conserved Plasmodium protein, unknown function			PF3D7_0613100	conserved Plasmodium protein, unknown function	unknown function	1	0	1	na	null	null	null	null	transport	integral to membrane, membrane
PBANKA_020500	conserved Plasmodium protein, unknown function			PF3D7_0108500	conserved Plasmodium protein, unknown function	unknown function	1	0	1	na	null	null	null	null	null	integral to membrane, membrane
PBANKA_020820	conserved Plasmodium protein, unknown function			PF3D7_0105100	conserved Plasmodium protein, unknown function	unknown function	1	0	1	na	null	null	null	null	null	null
PBANKA_021370	conserved Plasmodium protein, unknown function			PF3D7_0729600	conserved Plasmodium protein, unknown function	unknown function	1	0	1	na	null	null	null	null	null	null
PBANKA_030070	conserved Plasmodium protein, unknown function	-1.56	0.7	PF3D7_0202600.1/2	conserved Plasmodium protein, unknown function	unknown function	1	0	1	na	null	null	null	calcium ion binding	cytolysis, hemolysis by symbiont of host erythrocytes, pathogenesis	extracellular region, membrane
PBANKA_030100	conserved Plasmodium protein, unknown function			PF3D7_0216700.1/2	conserved Plasmodium protein, unknown function	unknown function	1	0	1	na	null	null	null	ATP binding, actin binding, calmodulin binding, motor activity	null	cytoplasm, myosin complex
PBANKA_030290	conserved Plasmodium protein, unknown function			PF3D7_0205200	conserved Plasmodium protein, unknown function	kinase	1	0	1	na	null	null	null	null	null	null
PBANKA_041780	conserved Plasmodium protein, unknown function			PF3D7_0903600.1	conserved Plasmodium protein, unknown function	unknown function	1	0	1	na	null	null	null	ATP binding, actin binding, calmodulin binding, motor activity	null	cytoplasm, myosin complex
PBANKA_050330	conserved Plasmodium protein, unknown function	-2.15	0.7	PF3D7_1019100	conserved Plasmodium protein, unknown function	unknown function	1	0	1	na	null	null	null	protein binding	null	cytoplasm
PBANKA_050770	conserved Plasmodium protein, unknown function			PF3D7_1023500	conserved Plasmodium protein, unknown function	unknown function	1	0	1	na	null	null	null	null	null	null
PBANKA_050830	conserved Plasmodium protein, unknown function			PF3D7_1024100	conserved Plasmodium protein, unknown function	unknown function	1	0	1	na	null	null	null	null	null	cytoplasm, nucleus
PBANKA_051000	conserved Plasmodium protein, unknown function			PF3D7_1025800	conserved Plasmodium protein, unknown function	unknown function	1	0	1	na	null	null	null	null	null	null
PBANKA_051040	conserved Plasmodium protein, unknown function			PF3D7_1026200	conserved Plasmodium protein, unknown function	unknown function	1	0	1	na	null	null	null	methyltransferase activity, nucleic acid binding	methylation	null
PBANKA_051290	conserved Plasmodium protein, unknown function			PF3D7_1028800	conserved Plasmodium protein, unknown function	unknown function	1	0	1	na	null	null	null	null	null	integral to membrane, membrane
PBANKA_051420	conserved Plasmodium protein, unknown function			PF3D7_1030200	conserved Plasmodium protein, unknown function	unknown function	1	0	1	na	null	null	null	nucleotide binding	null	cytoplasm, membrane, nucleus
PBANKA_051940	conserved Plasmodium protein, unknown function					unknown function	1	0	1	na	null	null	null	na	na	na
PBANKA_052370	conserved Plasmodium protein, unknown function			PF3D7_0423300	conserved Plasmodium protein, unknown function	unknown function	1	0	1	na	null	null	null	GTP binding, GTPase activity, translation initiation factor activity	translation, translational initiation	apicoplast, cytoplasm, intracellular
PBANKA_060580	conserved Plasmodium protein, unknown function	-4.98	0.75	PF3D7_1207300	conserved Plasmodium protein, unknown function	unknown function	1	0	1	na	null	null	null	DNA binding, hydrolase activity, transferase activity	null	apicoplast, cytoplasm
PBANKA_060750	conserved Plasmodium protein, unknown function			PF3D7_1209000	conserved Plasmodium protein, unknown function	mRNA	1	0	1	na	null	null	null	null	null	cytoplasm, nuclear periphery
PBANKA_061490	conserved Plasmodium protein, unknown function			PF3D7_0717200	conserved Plasmodium protein, unknown function	unknown function	1	0	1	na	null	null	null	null	null	membrane
PBANKA_061560	conserved Plasmodium protein, unknown function			PF3D7_0717900	thioredoxin-like protein	redoxmeth	1	0	1	na	null	null	null	null	null	null
PBANKA_061640	conserved Plasmodium protein, unknown function	-2.18	0.75	PF3D7_0718800	conserved Plasmodium protein, unknown function	unknown function	1	0	1	na	null	null	null	null	null	integral to membrane, membrane
PBANKA_070340	conserved Plasmodium protein, unknown function					unknown function	1	0	1	na	null	null	null	na	na	na
PBANKA_071280	conserved Plasmodium protein, unknown function			PF3D7_0818000	conserved Plasmodium protein, unknown function	RNA	1	0	1	na	null	null	null	null	null	null
PBANKA_071770	conserved Plasmodium protein, unknown function	-1.8	0.73	PF3D7_0415700	conserved Plasmodium protein, unknown function	unknown function	1	0	1	na	null	null	null	null	null	apicoplast, cytoplasm
PBANKA_080040	conserved Plasmodium protein, unknown function					unknown function	1	0	1	na	null	null	null			
PBANKA_080720	conserved Plasmodium protein, unknown function	-4.01	0.75	PF3D7_0318000	conserved Plasmodium protein, unknown function	unknown function	1	0	1	na	null	null	null	null	null	apicoplast

PBANKA_081550	conserved Plasmodium protein, unknown function			PF3D7_0914500	conserved Plasmodium protein, unknown function	unknown function	1	0	1	na	null	null	null	ATP binding	null	cytoplasm
PBANKA_082260	conserved Plasmodium protein, unknown function			PF3D7_0921700	conserved Plasmodium protein, unknown function	unknown function	1	0	1	na	null	null	null	nucleotide binding	null	apicoplast, cytoplasm
PBANKA_082460	conserved Plasmodium protein, unknown function			PF3D7_0923700	conserved Plasmodium protein, unknown function	unknown function	1	0	1	na	null	null	null	null	null	cytoplasm
PBANKA_082760	conserved Plasmodium protein, unknown function			PF3D7_0926800	conserved Plasmodium protein, unknown function	unknown function	1	0	1	na	null	null	null	receptor binding	cell differentiation, multicellular organismal development, signal transduction	extracellular region
PBANKA_083040	conserved Plasmodium protein, unknown function	-7.37	0.75	PF3D7_0929600	conserved Plasmodium protein, unknown function	unknown function	1	0	1	different in fertilization, ookinete, sporozoite and liver stage; (tagged) also different in oocyst	null	null	null	null	null	null
PBANKA_083500	conserved Plasmodium protein, unknown function			PF3D7_0934200	conserved Plasmodium protein, unknown function	unknown function	1	0	1	na	null	null	null	structural constituent of ribosome	translation	intracellular, ribosome, small ribosomal subunit
PBANKA_083570	conserved Plasmodium protein, unknown function			PF3D7_0934900	conserved Plasmodium protein, unknown function	unknown function	1	0	1	na	null	null	null	null	null	membrane
PBANKA_090800	conserved Plasmodium protein, unknown function			PF3D7_1141000	conserved Plasmodium protein, unknown function	unknown function	1	0	1	na	null	null	null	null	null	cytoplasm
PBANKA_091290	conserved Plasmodium protein, unknown function					unknown function	1	0	1	na	null	null	null	null	null	null
PBANKA_093750	conserved Plasmodium protein, unknown function	-3.2	0.75	PF3D7_1110100	conserved Plasmodium protein, unknown function	lipid binding	1	0	1	na	null	null	null	calcium ion binding, diacylglycerol binding, protein binding, zinc ion binding	intracellular signal transduction	integral to membrane, plasma membrane
PBANKA_100350	conserved Plasmodium protein, unknown function			PF3D7_0405800	conserved Plasmodium protein, unknown function	unknown function	1	0	1	na	null	null	null	null	null	null
PBANKA_100500	conserved Plasmodium protein, unknown function			PF3D7_0407400	conserved Plasmodium protein, unknown function	unknown function	1	0	1	na	null	null	null	ATP binding	null	null
PBANKA_100560	conserved Plasmodium protein, unknown function	-5.22	0.75	PF3D7_0408000	conserved Plasmodium protein, unknown function	unknown function	1	0	1	na	null	null	null	null	null	null
PBANKA_100790	conserved Plasmodium protein, unknown function	-2.55	0.74	PF3D7_0410700	GTPase, putative	translation	1	0	1	na	null	null	null	ATP binding	null	cytoplasm
PBANKA_101270	conserved Plasmodium protein, unknown function			PF3D7_1432200	conserved Plasmodium protein, unknown function	unknown function	1	0	1	na	null	null	null	null	null	cytoplasm
PBANKA_101280	conserved Plasmodium protein, unknown function			PF3D7_1432100	conserved protein, unknown function	unknown function	1	0	1	na	null	null	null	voltage-gated anion channel activity	anion transport	external side of mitochondrial outer membrane, mitochondrial outer membrane, mitochondrion
PBANKA_101730	conserved Plasmodium protein, unknown function	-2.02	0.71	PF3D7_1427300	conserved Plasmodium protein, unknown function	unknown function	1	0	1	na	null	null	null	ATP binding, actin binding, calmodulin binding	null	cytoplasm, myosin complex
PBANKA_101920	conserved Plasmodium protein, unknown function			PF3D7_1425300	conserved Plasmodium protein, unknown function	unknown function	1	0	1	na	null	null	null	null	null	cytoplasm
PBANKA_102480	conserved Plasmodium protein, unknown function			PF3D7_1417900	conserved Plasmodium protein, unknown function	unknown function	1	0	1	na	null	null	null	null	null	null
PBANKA_103180	conserved Plasmodium protein, unknown function			PF3D7_1410700	conserved Plasmodium protein, unknown function	unknown function	1	0	1	na	null	null	null	null	null	apicoplast
PBANKA_103330	conserved Plasmodium protein, unknown function			PF3D7_1409200.2	conserved Plasmodium protein, unknown function	unknown function	1	0	1	na	null	null	null	null	null	apicoplast
PBANKA_103970	conserved Plasmodium protein, unknown function			PF3D7_1402200	conserved Plasmodium protein, unknown function	unknown function	1	0	1	na	null	null	null	ATP binding, glutamate-tRNA ligase activity	glutamyl-tRNA aminoacylation, translation	cytoplasm, membrane
PBANKA_110210	conserved Plasmodium protein, unknown function	-4.07	0.75	PF3D7_0502300	conserved Plasmodium protein, unknown function	unknown function	1	0	1	na	null	null	null	null	null	apicoplast
PBANKA_110530	conserved Plasmodium protein, unknown function			PF3D7_0505700	conserved Plasmodium membrane protein, unknown function	unknown function	1	0	1	na	null	null	null	ATP binding, actin binding, calmodulin binding, motor activity	null	cytoplasm, myosin complex
PBANKA_111690	conserved Plasmodium protein, unknown function			PF3D7_0617300	conserved Plasmodium protein, unknown function	unknown function	1	0	1	na	null	null	null	4 iron, 4 sulfur cluster binding, electron carrier activity, iron ion binding, molybdenum ion binding, nitrate reductase activity	Mo-molybdopterin cofactor biosynthetic process, nitrate assimilation, oxidation-reduction process, transport	periplasmic space
PBANKA_112460	conserved Plasmodium protein, unknown function			PF3D7_0625700	conserved Plasmodium protein, unknown function	unknown function	1	0	1	na	null	null	null	null	null	null
PBANKA_113070	conserved Plasmodium protein, unknown function			PF3D7_1354100	conserved Plasmodium protein, unknown function	unknown function	1	0	1	na	null	null	null	null	null	null
PBANKA_113130	conserved Plasmodium protein, unknown function			PF3D7_1354700	conserved Plasmodium protein, unknown function	unknown function	1	0	1	na	null	null	null	GTP binding, GTPase activity, translation initiation factor activity	translation, translational initiation	intracellular
PBANKA_113800	conserved Plasmodium protein, unknown function			PF3D7_1362000	conserved Plasmodium protein, unknown function	unknown function	1	0	1	na	null	null	null	null	null	null
PBANKA_113950	conserved Plasmodium protein, unknown function			PF3D7_1363500	DNase I-like protein, putative	DNA repair	1	0	1	na	null	null	null	RNA binding, exonuclease activity, magnesium ion binding, nuclease activity, poly(A)-specific ribonuclease activity, protein binding	regulation of transcription, DNA-dependent, transcription, DNA-dependent	cytoplasm, nucleus
PBANKA_120180	conserved Plasmodium protein, unknown function	-3.57	0.75	PF3D7_1003400	conserved Plasmodium protein, unknown function	unknown function	1	0	1	na	null	null	null	ATP binding, actin binding, calmodulin binding, motor activity	null	membrane, myosin complex

PBANKA_120350	conserved Plasmodium protein, unknown function			PF3D7_1005300	conserved Plasmodium protein, unknown function	unknown function	1	0	1	na	null	null	null	null	null	apicoplast, membrane
PBANKA_121060	conserved Plasmodium protein, unknown function			PF3D7_1012200	conserved Plasmodium protein, unknown function	unknown function	1	0	1	na	null	null	null	null	null	apicoplast, membrane, nucleus
PBANKA_121330	conserved Plasmodium protein, unknown function	-2.24	0.7	PF3D7_1014900	conserved Plasmodium protein, unknown function	unknown function	1	0	1	na	null	null	null	ATP binding, actin binding, calmodulin binding, motor activity	null	cytoplasm, membrane, myosin complex
PBANKA_121490	conserved Plasmodium protein, unknown function			PF3D7_0323600	conserved Plasmodium protein, unknown function	unknown function	1	0	1	na	null	null	null	null	null	null
PBANKA_122590	conserved Plasmodium protein, unknown function	-2.99	0.75	PF3D7_0804700	conserved Plasmodium protein, unknown function	unknown function	1	0	1	na	null	null	null	null	null	null
PBANKA_123500	conserved Plasmodium protein, unknown function			PF3D7_0520200	conserved Plasmodium protein, unknown function	unknown function	1	0	1	na	null	null	null	(+)-abscisic acid 8'-hydroxylase activity	null	null
PBANKA_123830	conserved Plasmodium protein, unknown function			PF3D7_0523500	outer arm dynein lc3, putative	dynein/kinesin	1	0	1	na	null	null	null	null	null	null
PBANKA_124440	conserved Plasmodium protein, unknown function			PF3D7_0530000	conserved Plasmodium protein, unknown function	unknown function	1	0	1	na	null	null	null	null	null	nucleus
PBANKA_130700	conserved Plasmodium protein, unknown function			PF3D7_1443100	conserved Plasmodium protein, unknown function	unknown function	1	0	1	na	null	null	null	null	null	nucleus
PBANKA_131530	conserved Plasmodium protein, unknown function	-4.48	0.75	PF3D7_1451600	LCCL-like protein (FNPA)	adhesin	1	0	1	different in oocyst and sporozoite; (tagged) different in fertilization and ookinete	null	null	null	null	null	null
PBANKA_131590	conserved Plasmodium protein, unknown function			PF3D7_1452200	aminomethyltransferase, putative	post-translational processing	1	0	1	na	null	null	null	null	null	null
PBANKA_132250	conserved Plasmodium protein, unknown function			PF3D7_1458800	conserved Plasmodium protein, unknown function	unknown function	1	0	1	na	null	null	null	null	null	cytoplasm, membrane
PBANKA_133110	conserved Plasmodium protein, unknown function			PF3D7_1467800	conserved Plasmodium protein, unknown function	unknown function	1	0	1	na	null	null	null	null	null	null
PBANKA_134250	conserved Plasmodium protein, unknown function	-5.93	0.75	PF3D7_1327300	conserved Plasmodium protein, unknown function	unknown function	1	0	1	na	null	null	null	ATP binding, actin binding, calmodulin binding, motor activity	null	cytoplasm, myosin complex
PBANKA_135310	conserved Plasmodium protein, unknown function			PF3D7_1339700	conserved Plasmodium protein, unknown function	unknown function	1	0	1	na	null	null	null	ATP binding, actin binding, calmodulin binding, motor activity	null	cytoplasm, myosin complex
PBANKA_135360	conserved Plasmodium protein, unknown function			PF3D7_1340200	conserved Plasmodium protein, unknown function	unknown function	1	0	1	na	null	null	null	null	null	null
PBANKA_135950	conserved Plasmodium protein, unknown function	-1.79	0.7	PF3D7_1346600	conserved Plasmodium protein, unknown function	unknown function	1	0	1	na	null	null	null	structural molecule activity	null	null
PBANKA_140520	conserved Plasmodium protein, unknown function			PF3D7_1306700	conserved Plasmodium protein, unknown function	unknown function	1	0	1	na	null	null	null	metal ion binding, nucleotide binding, protein binding, transferase activity	regulation of transcription, DNA-dependent	cytoplasm, membrane, nucleus
PBANKA_141720	conserved Plasmodium protein, unknown function	-6.69	0.75	PF3D7_1318700	conserved Plasmodium protein, unknown function	unknown function	1	0	1	na	null	null	null	ATP binding	null	cytoplasm
PBANKA_142080	conserved Plasmodium protein, unknown function	-3.66	0.75	PF3D7_0714200	conserved Plasmodium protein, unknown function	unknown function	1	0	1	na	null	null	null	ATP binding, protein binding	null	null
PBANKA_142140	conserved Plasmodium protein, unknown function			PF3D7_0715100	conserved Plasmodium protein, unknown function	unknown function	1	0	1	na	null	null	null	protein binding	regulation of transcription, DNA-dependent, transcription, DNA-dependent	cytoplasm, nucleus
PBANKA_142150	conserved Plasmodium protein, unknown function					unknown function	1	0	1	na	null	null	null	na	na	na
PBANKA_142300	conserved Plasmodium protein, unknown function			PF3D7_0814500	conserved Plasmodium protein, unknown function	unknown function	1	0	1	na	null	null	null	null	null	null
PBANKA_144710	conserved Plasmodium protein, unknown function			PF3D7_1232400	conserved protein, unknown function	protein-protein interaction	1	0	1	na	null	null	null	null	null	null
PBANKA_145080	conserved Plasmodium protein, unknown function			PF3D7_1236200	conserved Plasmodium protein, unknown function	unknown function	1	0	1	na	null	null	null	null	null	cytoplasm
PBANKA_145110	conserved Plasmodium protein, unknown function	-6.17	0.75	PF3D7_1236500	conserved Plasmodium protein, unknown function	unknown function	1	0	1	na	null	null	null	3-dehydroquinate dehydratase activity	aromatic amino acid family biosynthetic process	null
PBANKA_101760	conserved Plasmodium protein, unknown function			PF3D7_1427000	conserved Plasmodium protein, unknown function	unknown function	1	0	1	na	null	protein folding, protein transport	null			
PBANKA_130570	conserved Plasmodium protein, unknown function			PF3D7_1441800	SNF7 family protein, putative	large tethering	1	0	1	na	null	protein transport	null			
PBANKA_092140	conserved Plasmodium protein, unknown function			PF3D7_1127000	protein phosphatase, putative	phosphatase	1	0	1	na	phosphatase activity	dephosphorylation	null			
PBANKA_140330	conserved Plasmodium protein, unknown function					unknown function	1	0	1	na	phosphatase activity	null	null			
PBANKA_010580	conserved Plasmodium protein, unknown function			PF3D7_0607200	conserved Plasmodium membrane protein, unknown function	unknown function	1	0	1	na	protein binding, zinc ion binding	null	null			

PBANKA_092500	conserved Plasmodium protein, unknown function			PF3D7_1123300	conserved Plasmodium protein, unknown function	transcription	1	0	1	na	protein binding, zinc ion binding	null	null			
PBANKA_093270	conserved Plasmodium protein, unknown function (PCAS_091160:exon:5)			PF3D7_1115000	conserved Plasmodium protein, unknown function	unknown function	1	0	1	na	null	null	null	null	null	null
PBANKA_041540	conserved Plasmodium protein, unknown function, pseudogene					unknown function	0	1	1	na	null	null	null	GPI anchor binding, heparan sulfate proteoglycan binding	B cell activation involved in immune response, cell-cell adhesion, entry into host cell	null
PBANKA_121800	conserved Plasmodium protein, unknown function,pyrazinamidase/nicoti	-3.63	0.75	PF3D7_0320500	conserved Plasmodium protein, unknown function	unknown function	1	0	1	na	catalytic activity	metabolic process	null			
PBANKA_072080	conserved Plasmodium protein, unknwon function			PF3D7_0418700	conserved Plasmodium protein, unknown function	RNA	0	1	1	na	null	null	null	null	null	nucleus
PBANKA_060460	conserved protein, unknown function	-1.84	0.7	PF3D7_1205900	conserved protein, unknown function	unknown function	1	0	1	na	null	null	null	null	null	integral to membrane, membrane
PBANKA_000370	conserved rodent malaria protein, unknown function						0	1	1	na	null	null	null			
PBANKA_000520	conserved rodent malaria protein, unknown function					unknown function	1	0	1	na	null	null	null			
PBANKA_122220	conserved rodent malaria protein, unknown function					unknown function	1	0	1	na	null	null	null	na	na	na
PBANKA_142440	CS domain protein, putative			PF3D7_0813200	CS domain protein, putative	adhesin	1	0	1	na	null	null	null	null	null	null
PBANKA_103230	cytidine triphosphate synthetase, putative			PF3D7_1410200	cytidine triphosphate synthetase	pyrimidine metabolism	1	0	1	na	CTP synthase activity	pyrimidine nucleotide biosynthetic process	null			
PBANKA_060080	cytochrome c oxidase assembly protein COX19, putative (COX19)	-1.74	0.71	PF3D7_1201800	cytochrome c oxidase assembly protein, putative	redoxmeth	1	0	1	na	null	null	null	na	na	na
PBANKA_132630	cytochrome c1 precursor, putative			PF3D7_1462700	cytochrome c1 precursor, putative	redoxmeth	1	0	1	na	electron carrier activity, heme binding, iron ion binding	null	null			
PBANKA_031270	DEAD/DEAH helicase, putative	-3.02	0.75	PF3D7_0216000	DEAD/DEAH box helicase, putative	RNA metabolism	1	0	1	na	ATP binding, DNA binding, helicase activity	null	null			
PBANKA_041520	developmental protein, putative			PF3D7_0906100	developmental protein, putative	large tethering	1	0	1	na	null	protein transport	null			
PBANKA_102890	diphthamide synthesis protein, putative			PF3D7_1413800	diphthamide synthesis protein, putative	ribosome assembly	1	0	1	na	null	null	null	null	null	null
PBANKA_130190	DNA repair metallo-beta-lactamase protein, putative			PF3D7_1474300	DNA repair metallo-beta-lactamase protein, putative	DNA repair	1	0	1	na	null	null	null	null	null	null
PBANKA_070810	dynein light chain 1 (DLC1)			PF3D7_0822500	dynein light chain 1 (DLC1)	dynein/kinesin	1	0	1	na	protein binding	null	null			
PBANKA_093360	dynein light chain 2, putative			PF3D7_1114000	dynein light chain type 2, putative	dynein/kinesin	1	0	1	na	null	null	null	null	null	null
PBANKA_120260	e3 ubiquitin-protein ligase, putative	-4.42	0.75	PF3D7_1004300	zinc finger, C3HC4 type, putative	proteasome	1	0	1	na	protein binding, zinc ion binding	null	null			
PBANKA_123250	eukaryotic translation initiation factor 3 subunit, putative			PF3D7_0517700	eukaryotic translation initiation factor, putative	translation	1	0	1	na	RNA binding, translation initiation factor activity	translational initiation	null			
PBANKA_050430	flagellar outer arm dynein-associated protein, putative			PF3D7_1020100	flagellar outer arm dynein-associated protein, putative	dynein/kinesin	1	0	1	na	null	null	null	null	null	membrane
PBANKA_040470	glutaredoxin 1, putative (GRX1)			PF3D7_0306300	glutaredoxin 1 (GRX1)	redoxmeth	1	0	1	na	electron carrier activity, protein disulfide oxidoreductase activity	cell redox homeostasis	null			
PBANKA_041040	glycogen synthase kinase 3 (GSK3)			PF3D7_0312400	glycogen synthase kinase 3 (GSK3)	kinase	1	0	1	na	ATP binding, protein serine/threonine kinase activity, protein tyrosine kinase activity	protein phosphorylation	null			
PBANKA_121030	heme oxygenase, putative (HO)			PF3D7_1011900	heme oxygenase (HO)	redoxmeth	1	0	1	na	null	null	null	ATP binding	null	apicoplast
PBANKA_140790	HORMA domain protein, putative	-5.85	0.75	PF3D7_1309400	HORMA domain protein, putative	microtubule	1	0	1	na	null	mitosis	null			
PBANKA_061090	HSP40, subfamily A, putative			PF3D7_1437900	HSP40, subfamily A, putative	chaperone	1	0	1	na	heat shock protein binding, unfolded protein binding	protein folding	null			
PBANKA_093600	inositol-5 phosphatase, putative			PF3D7_1111800	peptidyl-prolyl cis-trans isomerase, putative	chaperone	1	0	1	na	null	null	null	null	null	null
PBANKA_142880	iron-sulfur subunit of succinate dehydrogenase, putative			PF3D7_1212800	iron-sulfur subunit of succinate dehydrogenase	TCA	1	0	1	na	electron carrier activity, iron-sulfur cluster binding, oxidoreductase activity	tricarboxylic acid cycle	null			
PBANKA_135860	isocitrate dehydrogenase (NADP), mitochondrial precursor, putative (IDH)			PF3D7_1345700	isocitrate dehydrogenase (NADP), mitochondrial precursor (IDH)	TCA	1	0	1	na	NAD binding, isocitrate dehydrogenase (NADP+) activity, magnesium ion binding	isocitrate metabolic process, oxidation-reduction process	null			
PBANKA_120820	kinesin-4, putative					dynein/kinesin	1	0	1	na	ATP binding, microtubule motor activity	microtubule-based movement	null			

PBANKA_011020	lsm12, putative	-1.89	0.73	PF3D7_0611900	lsm12, putative	mRNA	1	0	1	na	null	null	null	null	null	null
PBANKA_122020	lysophospholipase, putative					lipase	1	0	1	na	null	null	null	na	na	na
PBANKA_090130	merozoite adhesive erythrocytic binding protein (MAEBL)					unknown function	1	0	1	different in sporozoite and liver stage	null	null	null	binding	null	apical complex, membrane
PBANKA_120780	metallopeptidase, putative			PF3D7_1009500	metallopeptidase, putative	sumoylation	1	0	1	na	null	null	null	null	null	null
PBANKA_101020	methionine aminopeptidase, type II, putative			PF3D7_1434600	methionine aminopeptidase 2 (MetAP2)	post-translational processing	1	0	1	na	aminopeptidase activity, metalloexopeptidase activity	cellular process, proteolysis	null			
PBANKA_051580	mitochondrial preribosomal assembly protein rimM precursor, putative			PF3D7_1032000	mitochondrial preribosomal assembly protein rimM precursor, putative	ribosome mito	1	0	1	na	null	rRNA processing	null			
PBANKA_020440	mRNA cleavage factor-like protein, putative			PF3D7_0109200	mRNA cleavage factor-like protein, putative	mRNA	1	0	1	na	null	null	null	null	null	null
PBANKA_124070	NIMA related kinase 2 (NEK2)			PF3D7_0525900	NIMA related kinase 2 (NEK2)	kinase	1	0	1	different in fertilization, ookinete and oocyst	ATP binding, protein serine/threonine kinase activity, protein tyrosine kinase activity	protein phosphorylation	null			
PBANKA_080560	O-sialoglycoprotein endopeptidase, putative			PF3D7_0708300	O-sialoglycoprotein endopeptidase, putative	protease	1	0	1	KO not successful	ATP binding, protein kinase activity	protein phosphorylation	null			
PBANKA_130030	Pb-fam-1 protein, fragment					Pb-fam-1 protein	1	0	1	na	null	null	null	na	na	na
PBANKA_100730	peptide chain release factor 2, putative			PF3D7_0409700	peptide chain release factor 2, putative	translation	1	0	1	na	translation release factor activity	translational termination	null			
PBANKA_080930	peptide deformylase, putative			PF3D7_0907900	peptide deformylase (PDF)	protease	1	0	1	na	iron ion binding, peptide deformylase activity	translation	null			
PBANKA_093580	peptidyl-prolyl cis-trans isomerase, putative			PF3D7_1113700	glyoxalase I (GloI)	redoxmeth	1	0	1	na	null	protein folding	null			
PBANKA_060140	peptidyl-prolyl cis-trans isomerase, putative (CYP26)			PF3D7_1202400	peptidyl-prolyl cis-trans isomerase (CYP26)	chaperone	1	0	1	na	peptidyl-prolyl cis-trans isomerase activity	null	null			
PBANKA_100480	peptidyl-tRNA hydrolase, putative			PF3D7_0407200	peptidyl-tRNA hydrolase PTH2, putative	translation	1	0	1	na	aminoacyl-tRNA hydrolase activity	translation	null			
PBANKA_112870	phosphatidylinositol/phosphatidylcholine transfer protein, putative			PF3D7_0629900	sec14-like cytosolic factor or phosphatidylinositol/phosphatidylcholine transfer protein, putative	trafficking	1	0	1	na	null	null	null	binding	vesicle-mediated transport	Golgi apparatus
PBANKA_101450	plasmepsin IX, putative			PF3D7_1430200	plasmepsin IX	plasmepsin, protease	1	0	1	na	aspartic-type endopeptidase activity	proteolysis	null			
PBANKA_031610	Plasmodium exported protein, unknown function					unknown function	1	0	1	na	null	null	null			
PBANKA_000590	Plasmodium exported protein, unknown function, pseudogene					unknown function	1	0	1	na	null	null	null			
PBANKA_133720	protein transport protein sft2, putative			PF3D7_1321800	SFT2-like protein, putative	transporter, trafficking	1	0	1	na	null	null	null	null	null	membrane
PBANKA_131720	pyridine nucleotide transhydrogenase, putative	-3.14	0.71	PF3D7_1453500	pyridine nucleotide transhydrogenase, putative	pyrimidine metabolism	1	0	1	na	NAD(P)+ transhydrogenase activity, binding, electron carrier activity	metabolic process, oxidation-reduction process	null			
PBANKA_121020	QF122 antigen, putative	-3.15	0.75	PF3D7_1011800	QF122 antigen	mRNA	1	0	1	na	RNA binding	null	null			
PBANKA_092470	RAP protein, putative			PF3D7_1123600	RAP protein, putative	mRNA	1	0	1	na	null	null	null	null	null	null
PBANKA_040100	regulatory protein, putative			PF3D7_0302000	golgi organization and biogenesis factor, putative		1	0	1	na	null	null	null	null	null	null
PBANKA_000160	reticulocyte binding protein, putative, fragment						1	0	1	na	null	null	null			
PBANKA_091460	RNA (uracil-5-)methyltransferase, putative	-4.16	0.75	PF3D7_1133800	RNA (uracil-5-)methyltransferase, putative	RNA	1	0	1	na	RNA methyltransferase activity	RNA processing	null			
PBANKA_061370	RNA binding protein, putative			PF3D7_0716000	RNA binding protein, putative	mRNA	1	0	1	na	nucleic acid binding	null	null			
PBANKA_081770	RNA-binding protein musashi, putative (HoMu)	-1.75	0.71	PF3D7_0916700	RNA-binding protein musashi, putative (HoMu)	mRNA	1	0	1	na	nucleic acid binding	null	null			
PBANKA_141920	RNA-binding protein, putative	-1.95	0.73	PF3D7_1320900	RNA-binding protein, putative	mRNA	1	0	1	na	nucleic acid binding	null	null			
PBANKA_113570	RNA-binding protein, putative			PF3D7_1359400	rRNA associated RNA binding protein, putative	RNA	1	0	1	na	RNA binding, protein binding	null	null			
PBANKA_081900	secreted acid phosphatase, putative,glideosome-associated protein 50,	-4.4	0.75	PF3D7_0918000	glideosome-associated protein 50,secreted acid phosphatase (GAP50)	gliding motility	1	0	1	na	null	null	null	protein anchor	null	endoplasmic reticulum, inner membrane complex

PBANKA_110300	Ser/Arg-rich splicing factor, putative			PF3D7_0503300	Ser/Arg-rich splicing factor, putative	mRNA	1	0	1	na	nucleic acid binding	null	null			
PBANKA_030510	serine repeat antigen 1 (SERA1)			PF3D7_0207400	serine repeat antigen 7 (SERA7)	protease	1	0	1	not different from wild type	cysteine-type peptidase activity	proteolysis	null			
PBANKA_131370	serine/threonine protein kinase, putative			PF3D7_1450000	serine/threonine protein kinase, putative	kinase	1	0	1	KO not successful	ATP binding, protein serine/threonine kinase activity, protein tyrosine kinase activity	protein phosphorylation	null			
PBANKA_141360	serine/threonine protein kinase, putative			PF3D7_1315100	serine/threonine protein kinase (PK9)	kinase	1	0	1	KO not successful	ATP binding, protein serine/threonine kinase activity, protein tyrosine kinase activity	protein phosphorylation	null			
PBANKA_093250	SET domain protein, putative	-6.41	0.75	PF3D7_1115200	SET domain protein, putative (SET7)	chromatin modifying prtoeins	1	0	1	na	null	null	null	methyltransferase activity, zinc ion binding	null	cytoplasm, nucleus
PBANKA_136300	small nuclear ribonucleoprotein E, putative (SNRPE)			PF3D7_1350200	small nuclear ribonucleoprotein E, putative (SNRPE)	mRNA	1	0	1	na	null	null	null	null	RNA processing, RNA splicing, via transesterification reactions	small nucleolar ribonucleoprotein complex
PBANKA_081100	small nuclear ribonucleoprotein Sm D3, putative (SNRPD3)			PF3D7_0909800	small nuclear ribonucleoprotein Sm D3, putative (SNRPD3)	mRNA	1	0	1	na	null	null	null	null	null	null
PBANKA_102790	small nuclear ribonucleoprotein-associated protein B, putative (SNRPB)			PF3D7_1414800	small nuclear ribonucleoprotein-associated protein B, putative (SNRPB)	mRNA	1	0	1	na	null	null	null	null	RNA splicing	spliceosomal complex
PBANKA_102320	splicing factor 3B subunit 4, putative (SF3B4)			PF3D7_1420000	splicing factor 3B subunit 4, putative (SF3B4)	mRNA	1	0	1	na	nucleic acid binding	null	null			
PBANKA_113680	SUMO ligase, putative			PF3D7_1360700	SUMO ligase, putative	sumoylation	1	0	1	na	zinc ion binding	null	null			
PBANKA_112240	superoxide dismutase, putative			PF3D7_0623500	superoxide dismutase (SOD2)	redoxmeth	1	0	1	na	metal ion binding, superoxide dismutase activity	oxidation-reduction process, superoxide metabolic process	null			
PBANKA_101850	transcription factor 3b, putative			PF3D7_1426100	basic transcription factor 3b, putative	transcription	1	0	1	na	null	null	null	null	null	nascent polypeptide-associated complex, transcription factor complex
PBANKA_130580	transcription factor TFIIH complex subunit Tfb5, putative			PF3D7_1441900	transcription factor TFIIH complex subunit Tfb5, putative	transcription	1	0	1	na	DNA binding	nucleotide-excision repair	null			
PBANKA_131970	transcription factor with AP2 domain(s), putative (ApiAP2)			PF3D7_1456000	transcription factor with AP2 domain(s) (ApiAP2)	transcription	1	0	1	na	sequence-specific DNA binding transcription factor activity	regulation of transcription, DNA-dependent	null			
PBANKA_123690	transcription initiation factor tfiid subunit 10, putative (TAF10)			PF3D7_0522200	conserved Plasmodium protein, unknown function	unknown function	1	0	1	na	null	null	null	null	null	cytoplasm
PBANKA_134160	translation initiation factor EIF-2B gamma subunit, putative			PF3D7_1326400	translation initiation factor EIF-2B gamma subunit, putative	translation	1	0	1	na	transferase activity	null	null			
PBANKA_093990	U6 snRNA-associated Sm-like protein LSm4, putative (LSM4)			PF3D7_1107000	U6 snRNA-associated Sm-like protein LSm4, putative (LSM4)	mRNA	1	0	1	na	null	null	null	null	null	null
PBANKA_060760	U6 snRNA-associated Sm-like protein LSm7, putative (LSM7)			PF3D7_1209200	U6 snRNA-associated Sm-like protein LSm7, putative (LSM7)	mRNA	1	0	1	na	null	null	null	null	RNA splicing, via transesterification reactions, nuclear mRNA splicing, via spliceosome	nucleus, small nucleolar ribonucleoprotein complex, spliceosomal complex
PBANKA_101770	ubiquinol-cytochrome c reductase hinge protein, putative			PF3D7_1426900	ubiquinol-cytochrome c reductase hinge protein, putative	redoxmeth	1	0	1	na	ubiquinol-cytochrome-c reductase activity	mitochondrial electron transport, ubiquinol to cytochrome c	null			
PBANKA_124200	ubiquitin carboxyl-terminal hydrolase, putative			PF3D7_0527200	ubiquitin carboxyl-terminal hydrolase, putative	proteasome	1	0	1	na	ubiquitin thiolesterase activity	ubiquitin-dependent protein catabolic process	null			
PBANKA_091060	ubiquitin-related modifier 1, putative (URM1)			PF3D7_1138100	ubiquitin related modifier homologue	proteasome	1	0	1	na	null	null	null	null	null	null
PBANKA_102290	zinc finger protein, putative			PF3D7_1420300	zinc finger protein, putative	transporter	1	0	1	na	null	null	null	null	null	null
PBANKA_123760	zinc finger protein, putative, fragment	-1.78	0.7	PF3D7_0522900	zinc finger protein, putative	RNA binding	1	0	1	na	nucleic acid binding, zinc ion binding	null	null			
PBANKA_071430	small heat shock protein HSP20 (HSP20)	-4.76	0.75	PF3D7_0816500	small heat shock protein HSP20, putative (HSP20)	chaperone	0	1	1	different in fertilization, ookinete, sporozoite and liver stage; (tagged) different in oocyst too	null	null	plasma membrane			
PBANKA_123400	vacuolar ATP synthetase, putative			PF3D7_0519200	vacuolar ATP synthetase	vacuolar ATP synthase	0	1	1	na	hydrogen ion transmembrane transporter activity	ATP synthesis coupled proton transport	proton-transporting V-type ATPase, V0 domain			
PBANKA_090890	vacuolar ATP synthase subunit f, putative			PF3D7_1140100	vacuolar ATP synthase subunit f, putative	vacuolar ATP synthase	0	1	1	na	hydrogen ion transporting ATP synthase activity, rotational mechanism, proton-transporting ATPase activity, rotational mechanism	ATP synthesis coupled proton transport	proton-transporting V-type ATPase, V1 domain			
PBANKA_093200	rhoptry neck protein 4, putative (RON4)			PF3D7_1116000	rhoptry neck protein 4 (RON4)		1	0	1	(mutated) different in sporozoite and liver stage	null	null	rhoptry			
PBANKA_133830	60S ribosomal protein L6, putative			PF3D7_1323100	60S ribosomal protein L6, putative	ribosome	1	0	1	na	rRNA binding, structural constituent of ribosome	translation	ribosome			
PBANKA_144620	DIM1 protein homolog, putative			PF3D7_1231500	DIM1 protein homolog, putative	ribosome assembly	0	1	1	na	null	mitosis	spliceosomal complex			
PBANKA_131780	tRNA intron endonuclease, putative			PF3D7_1454100	tRNA intron endonuclease, putative	translation	0	1	1	na	tRNA-intron endonuclease activity	tRNA splicing, via endonucleolytic cleavage and ligation	tRNA-intron endonuclease complex			
PBANKA_000330	BIR protein					BIR protein	0	0	0	na	null	null	null			

PBANKA_000340	BIR protein					BIR protein	0	0	0	na	null	null	null
PBANKA_000380	BIR protein					BIR protein	0	0	0	na	null	null	null
PBANKA_000490	conserved rodent malaria protein, unknown function					unknown function	0	0	0	na	null	null	null
PBANKA_000570	BIR protein					BIR protein	0	0	0	na	null	null	null
PBANKA_010060	schizont membrane associated cytoadherence protein (SMAC)						0	0	0	na	null	cytoadherence to microvasculature, mediated by symbiont protein	host cell cytoplasm
PBANKA_010070	Plasmodium exported protein, unknown function					unknown function	0	0	0	na	null	null	null
PBANKA_010080	conserved Plasmodium protein, unknown function			PF3D7_0602000	conserved Plasmodium protein, unknown function	microtubule	0	0	0	na	null	null	null
PBANKA_010270	conserved Plasmodium protein, unknown function			PF3D7_0603900	conserved Plasmodium protein, unknown function	unknown function	0	0	0	na	null	null	null
PBANKA_010410	aurora-related kinase 1, putative (ARK1)			PF3D7_0605300	serine/threonine protein kinase (ARK1)	kinase	0	0	0	KO not successful	ATP binding, protein serine/threonine kinase activity, protein tyrosine kinase activity	protein phosphorylation	null
PBANKA_010470	long chain polyunsaturated fatty acid elongation enzyme, putative			PF3D7_0605900	long chain polyunsaturated fatty acid elongation enzyme, putative	fatty acids synth	0	0	0	na	null	null	integral to membrane
PBANKA_010560	translation initiation factor IF-2, putative			PF3D7_0607000	translation initiation factor IF-2, putative	translation	0	0	0	na	GTP binding, GTPase activity	null	null
PBANKA_010860	Pre-mRNA-splicing factor SLU7, putative (SLU7)			PF3D7_0610100	Pre-mRNA-splicing factor SLU7, putative (SLU7)	mRNA	0	0	0	na	null	null	null
PBANKA_010870	conserved Plasmodium protein, unknown function	-1.9	0.73	PF3D7_0610200	conserved Plasmodium protein, unknown function	mRNA	0	0	0	na	nucleic acid binding	mRNA processing	null
PBANKA_011000	60S ribosomal protein L39, putative			PF3D7_0611700	60S ribosomal protein L39, putative	ribosome	0	0	0	na	structural constituent of ribosome	translation	intracellular, ribosome
PBANKA_011030	conserved Plasmodium protein, unknown function			PF3D7_0612000	conserved Plasmodium protein, unknown function	unknown function	0	0	0	na	null	null	null
PBANKA_011050	leucine-rich repeat protein (LRR6)	-2.6	0.72	PF3D7_0612200	leucine-rich repeat protein (LRR6)	kinase	0	0	0	na	protein binding	null	null
PBANKA_011130	conserved Plasmodium protein, unknown function			PF3D7_0613000.1/2	conserved Plasmodium protein, unknown function	unknown function	0	0	0	na	null	null	null
PBANKA_020020	Pb-fam-1 protein					Pb-fam-1 protein	0	0	0	na	null	null	null
PBANKA_020090	conserved rodent malaria protein, unknown function					unknown function	0	0	0	na	null	null	null
PBANKA_020160	early transcribed membrane protein (ETRAMP)					early transcribed membrane protein (ETRAMP)	0	0	0	na	null	null	null
PBANKA_020190	conserved Plasmodium protein, unknown function			PF3D7_0111900	conserved Plasmodium protein, unknown function	unknown function	0	0	0	na	null	null	null
PBANKA_020420	tubulin-specific chaperone a, putative			PF3D7_0109400	tubulin-specific chaperone a, putative	chaperone	0	0	0	na	unfolded protein binding	tubulin complex assembly	microtubule
PBANKA_020470	conserved Plasmodium protein, unknown function			PF3D7_0108800	conserved Plasmodium protein, unknown function	transporter	0	0	0	na	binding	transport	membrane
PBANKA_020510	mitochondrial carrier protein, putative			PF3D7_0108400.1/2	mitochondrial carrier protein, putative	transporter	0	0	0	na	binding	transport	mitochondrial inner membrane
PBANKA_020580	serine/threonine protein kinase, putative (IK2)			PF3D7_0107600	serine/threonine protein kinase, putative, EIK2	kinase	0	0	0	different in fertilization, ookinete, oocyst, sporozoite and liver stage (contradictory results)	ATP binding, protein serine/threonine kinase activity	protein phosphorylation	null
PBANKA_020630	centrin, putative			PF3D7_0107000	centrin-1 (CEN1)	chromatin	0	0	0	na	calcium ion binding	null	null
PBANKA_020650	Rab5c, GTPase, putative			PF3D7_0106800	Rab GTPase 5c (RAB5c)	trafficking	0	0	0	na	GTP binding, GTPase activity, protein binding	intracellular protein transport, nucleocytoplasmic transport, small GTPase mediated signal transduction	intracellular
PBANKA_020720	vacuolar ATP synthase subunit c, putative			PF3D7_0106100	vacuolar ATP synthase subunit c, putative	vacuolar ATP synthase	0	0	0	na	hydrolase activity, acting on acid anhydrides, catalyzing transmembrane movement of substances	ATP synthesis coupled proton transport	proton-transporting V-type ATPase, V1 domain
PBANKA_020760	asparagine-rich antigen, putative						0	0	0	na	null	null	null
PBANKA_020930	actin-related protein (ARP1)			PF3D7_0103800	actin-related protein (ARP1)	gliding motility	0	0	0	KO not successful	protein binding	null	null
PBANKA_021120	conserved Plasmodium protein, unknown function			PF3D7_0727100	conserved Plasmodium protein, unknown function	unknown function	0	0	0	na	null	null	null

PBANKA_021210	eukaryotic translation initiation factor 2 alpha subunit, putative			PF3D7_0728000	eukaryotic translation initiation factor 2 alpha subunit, putative	translation	0	0	0	na	RNA binding, translation initiation factor activity	translation	eukaryotic translation initiation factor 2 complex
PBANKA_021300	RNA-binding protein, putative			PF3D7_0728900	RNA binding protein, putative	mRNA	0	0	0	na	nucleic acid binding	null	null
PBANKA_021310	signal recognition particle subunit srp9, putative (SRP9)			PF3D7_0729000	signal recognition particle SRP9 (SRP9)	post-translational modification	0	0	0	na	7S RNA binding, protein binding	SRP-dependent cotranslational protein targeting to membrane, negative regulation of translational elongation	signal recognition particle
PBANKA_030060	Plasmodium exported protein, unknown function					unknown function	0	0	0	na	null	null	null
PBANKA_030110	conserved Plasmodium protein, unknown function	-2.99	0.75	PF3D7_0203200	conserved Plasmodium protein, unknown function	unknown function	0	0	0	na	null	null	null
PBANKA_030120	ERCC1 nucleotide excision repair protein, putative			PF3D7_0203300	ERCC1 nucleotide excision repair protein, putative	DNA repair	0	0	0	na	damaged DNA binding, endonuclease activity	DNA repair	nucleus
PBANKA_030150	nucleolar preribosomal assembly protein, putative			PF3D7_0203700	nucleolar preribosomal assembly protein, putative	ribosome assembly	0	0	0	na	null	null	null
PBANKA_030250	hexose transporter (HT)			PF3D7_0204700	hexose transporter (HT)	glycolysis	0	0	0	KO not successful; (tagged) different in fertilization, ookinete, oocyst, sporozoite and liver stage; (mutated) not different from wild type	substrate-specific transmembrane transporter activity	transmembrane transport, transport	integral to membrane, membrane
PBANKA_030620	phospholipase A2, putative			PF3D7_0209100	phospholipase A2, putative	utilization phospholipids	0	0	0	na	null	lipid metabolic process	null
PBANKA_030680	ATP-dependent RNA helicase UAP56, putative (UAP56)			PF3D7_0209800	ATP-dependent RNA helicase UAP56 (UAP56)	mRNA	0	0	0	na	ATP binding, ATP-dependent helicase activity, helicase activity, nucleic acid binding	null	null
PBANKA_031020	conserved Plasmodium protein, unknown function			PF3D7_0213300	conserved Plasmodium protein, unknown function	unknown function	0	0	0	na	null	null	null
PBANKA_031190	RING zinc finger protein, putative			PF3D7_0215100	RING zinc finger protein, putative	proteasome	0	0	0	na	protein binding, zinc ion binding	null	null
PBANKA_031240	DNA-directed RNA polymerase II second largest subunit, putative			PF3D7_0215700	DNA-directed RNA polymerase II second largest subunit, putative	transcription	0	0	0	na	DNA binding, DNA-directed RNA polymerase activity, ribonucleoside binding	transcription, DNA-dependent	null
PBANKA_031250	origin recognition complex subunit 5, putative (ORC5)			PF3D7_0215800	origin recognition complex subunit 5 (ORC5)	DNA replication	0	0	0	na	null	null	null
PBANKA_031410	conserved Plasmodium protein, unknown function			PF3D7_0217400	conserved Plasmodium protein, unknown function	unknown function	0	0	0	na	null	null	null
PBANKA_031420	calcium dependent protein kinase 1 (CDPK1)			PF3D7_0217500	calcium dependent protein kinase 1 (CDPK1)	kinase	0	0	0	different in oocyst; (mutated) fertilization and ookinete; (tagged) also in sporozoite	ATP binding, calcium ion binding, protein serine/threonine kinase activity, protein tyrosine kinase activity	protein phosphorylation	null
PBANKA_031450	40S ribosomal protein S26e, putative			PF3D7_0217800	40S ribosomal protein S26e, putative	ribosome	0	0	0	na	structural constituent of ribosome	translation	intracellular, ribosome
PBANKA_031560	40S ribosomal protein S30, putative			PF3D7_0219200	40S ribosomal protein S30, putative	ribosome	0	0	0	na	structural constituent of ribosome	translation	intracellular, ribosome
PBANKA_031600	replication factor C subunit 1, putative			PF3D7_0219600	replication factor C subunit 1, putative	DNA replication	0	0	0	na	ATP binding, DNA clamp loader activity	DNA replication	DNA replication factor C complex, intracellular
PBANKA_031620	Plasmodium exported protein, unknown function, fragment					unknown function	0	0	0	na	null	null	null
PBANKA_040040	BIR protein, pseudogene					BIR protein	0	0	0	na	null	null	null
PBANKA_040230	dihydrolipoamide acyltransferase, putative			PF3D7_0303700	dihydrolipoamide acyltransferase, putative	glycolysis, TCA	0	0	0	na	transferase activity, transferring acyl groups	metabolic process	null
PBANKA_040300	60S ribosomal protein L44, putative			PF3D7_0304400	60S ribosomal protein L44, putative	ribosome	0	0	0	na	structural constituent of ribosome	translation	intracellular, ribosome
PBANKA_040340	conserved Plasmodium protein, unknown function			PF3D7_0304900	conserved Plasmodium protein, unknown function	unknown function	0	0	0	na	null	null	null
PBANKA_040410	AP endonuclease (DNA-(apurinic or apyrimidinic site) lyase), putative			PF3D7_0305600	AP endonuclease (DNA-(apurinic or apyrimidinic site) lyase), putative	DNA repair	0	0	0	na	nuclease activity	DNA repair	null
PBANKA_040440	conserved Plasmodium protein, unknown function			PF3D7_0306000	conserved Plasmodium protein, unknown function	unknown function	0	0	0	na	null	null	null
PBANKA_040530	40S ribosomal protein S23, putative			PF3D7_0306900	40S ribosomal protein S23, putative	ribosome	0	0	0	na	structural constituent of ribosome	translation	intracellular, ribosome, small ribosomal subunit
PBANKA_040550	60S ribosomal protein L7, putative			PF3D7_0307200	60S ribosomal protein L7, putative	ribosome	0	0	0	na	structural constituent of ribosome	translation	intracellular, large ribosomal subunit, ribosome
PBANKA_040570	ATP-dependent Clp protease proteolytic subunit, putative (ClpP)			PF3D7_0307400	ATP-dependent Clp protease proteolytic subunit (ClpP)	chaperone	0	0	0	na	serine-type endopeptidase activity	proteolysis	apicoplast
PBANKA_040600	conserved Plasmodium protein, unknown function			PF3D7_0307700	conserved Plasmodium protein, unknown function	unknown function	0	0	0	na	DNA binding	null	null
PBANKA_040650	TCP-1/cpn60 chaperonin, putative			PF3D7_0308200	TCP-1/cpn60 chaperonin family, putative	chaperone	0	0	0	na	ATP binding, unfolded protein binding	protein folding	null

PBANKA_040770	60S acidic ribosomal protein P2, putative			PF3D7_0309600	60S acidic ribosomal protein P2, putative	ribosome	0	0	0	KO not successful	structural constituent of ribosome	translational elongation	intracellular, ribosome
PBANKA_040930	phosphatidylinositol 3- and 4-kinase, putative			PF3D7_0311300	phosphatidylinositol 3- and 4-kinase, putative	inositol phosphate metabolism	0	0	0	na	phosphotransferase activity, alcohol group as acceptor	null	null
PBANKA_040940	protein kinase, putative (PKRP)			PF3D7_0311400	protein kinase, putative	kinase	0	0	0	different in fertilization, ookinete, oocyst, sporozoite and liver stage	ATP binding, protein serine/threonine kinase activity, protein tyrosine kinase activity	protein phosphorylation	null
PBANKA_041000	conserved Plasmodium protein, unknown function			PF3D7_0312000	conserved Plasmodium protein, unknown function	unknown function	0	0	0	na	null	null	null
PBANKA_041130	conserved Plasmodium protein, unknown function			PF3D7_0313600	conserved Plasmodium protein, unknown function	unknown function	0	0	0	na	null	null	null
PBANKA_041310	conserved Plasmodium protein, unknown function					RNA	0	0	0	na	null	microtubule cytoskeleton organization	microtubule organizing center, spindle pole
PBANKA_041460	40S ribosomal protein S15A, putative			PF3D7_0316800	40S ribosomal protein S15A, putative	ribosome	0	0	0	na	structural constituent of ribosome	translation	intracellular, ribosome
PBANKA_041480	arginase			PF3D7_0906500	arginase	AA metabolism	0	0	0	na	arginase activity, metal ion binding	arginine metabolic process	null
PBANKA_041660	replication protein A1, small fragment			PF3D7_0904800	replication protein A1, small fragment	DNA repair	0	0	0	na	nucleic acid binding	null	null
PBANKA_041740	GTPase activating protein, putative	-1.86	0.73	PF3D7_0904000	GTPase activator, putative	trafficking	0	0	0	na	Rab GTPase activator activity	regulation of Rab GTPase activity	intracellular
PBANKA_041750	60S ribosomal protein L32, putative			PF3D7_0903900	60S ribosomal protein L32, putative	ribosome	0	0	0	na	structural constituent of ribosome	translation	intracellular, ribosome
PBANKA_041770	alpha tubulin 1			PF3D7_0903700	alpha tubulin 1	gliding motility	0	0	0	KO not successful	GTP binding, GTPase activity, structural molecule activity	microtubule-based movement, protein polymerization	microtubule, protein complex
PBANKA_050070	Pb-fam-1 protein					Pb-fam-1 protein	0	0	0	na	null	null	null
PBANKA_050110	early transcripted membrane protein (SEP3)					early transcribed membrane protein (ETRAMP)	0	0	0	KO not successful; (tagged) fertilization, ookinete and oocyst	null	null	null
PBANKA_050170	phosphomannomutase, putative			PF3D7_1017400	phosphomannomutase, putative	mannose metabolism	0	0	0	na	phosphomannomutase activity	mannose biosynthetic process	cytoplasm
PBANKA_050250	serine/threonine protein phosphatase, putative	-3.02	0.74	PF3D7_1018200	serine/threonine protein phosphatase, putative	phosphatase	0	0	0	na	hydrolase activity	null	null
PBANKA_050280	conserved Plasmodium protein, unknown function			PF3D7_1018600	conserved Plasmodium protein, unknown function	translation	0	0	0	na	null	null	null
PBANKA_050350	conserved Plasmodium protein, unknown function	-2.08	0.71	PF3D7_1019300	zinc finger protein, putative	nucleic acid binding	0	0	0	na	nucleic acid binding, zinc ion binding	null	null
PBANKA_050360	60S ribosomal protein L30e, putative			PF3D7_1019400	60S ribosomal protein L30e, putative	ribosome	0	0	0	na	structural constituent of ribosome	translation	intracellular, ribosome
PBANKA_050440	conserved Plasmodium protein, unknown function			PF3D7_1020200	kinesin, putative	dynein/kinesin	0	0	0	na	null	null	null
PBANKA_050510	ADP-ribosylation factor, putative (ARF1)			PF3D7_1020900	ADP-ribosylation factor (ARF1)	trafficking	0	0	0	na	GTP binding	intracellular protein transport, small GTPase mediated signal transduction	intracellular
PBANKA_050540	conserved Plasmodium protein, unknown function			PF3D7_1021200	conserved Plasmodium protein, unknown function	unknown function	0	0	0	na	null	null	null
PBANKA_050580	deoxyribose-phosphate aldolase, putative			PF3D7_1021600	deoxyribose-phosphate aldolase, putative	gliding motility	0	0	0	na	deoxyribose-phosphate aldolase activity	deoxyribonucleotide catabolic process	cytoplasm
PBANKA_050630	conserved Plasmodium protein, unknown function			PF3D7_1022100	conserved Plasmodium protein, unknown function	unknown function	0	0	0	na	null	null	null
PBANKA_050660	pre-mRNA splicing factor, putative	-2.25	0.75	PF3D7_1022400	pre-mRNA splicing factor, putative	mRNA	0	0	0	na	nucleic acid binding	null	null
PBANKA_050670	citrate synthase, mitochondrial precursor, putative			PF3D7_1022500	citrate synthase, mitochondrial precursor, putative	TCA	0	0	0	(tagged) different in oocyst and liver stage	transferase activity, transferring acyl groups, acyl groups converted into alkyl on transfer	cellular carbohydrate metabolic process	null
PBANKA_050810	chromodomain-helicase-DNA-binding protein 1, putative (CHD1)			PF3D7_1023900	chromodomain-helicase-DNA-binding protein 1 homolog, putative (CHD1)	chromatin	0	0	0	KO not successful	ATP binding, DNA binding, chromatin binding, helicase activity	chromatin assembly or disassembly	chromatin, nucleus
PBANKA_050860	conserved Plasmodium protein, unknown function			PF3D7_1024400	conserved Plasmodium protein, unknown function	unknown function	0	0	0	na	null	null	null
PBANKA_050920	formin 2, putative			PF3D7_1025000	formin 2, putative	gliding motility	0	0	0	na	null	null	null
PBANKA_050930	glucosamine--fructose-6-phosphate aminotransferase, putative			PF3D7_1025100	glucosamine-fructose-6-phosphate aminotransferase, putative	chitin precursor	0	0	0	na	carbohydrate binding	carbohydrate metabolic process	null
PBANKA_051010	conserved Plasmodium protein, unknown function			PF3D7_1025900	conserved Plasmodium protein, unknown function	unknown function	0	0	0	na	null	null	null

PBANKA_051020	conserved Plasmodium protein, unknown function			PF3D7_1026000	conserved Plasmodium protein, unknown function	unknown function	0	0	0	na	null	null	null
PBANKA_051060	conserved Plasmodium protein, unknown function			PF3D7_1026400	CDH1, WD-repeat protein, putative	kinetochore	0	0	0	different in fertilization and ookinete	null	cytokinesis	nucleus
PBANKA_051080	conserved Plasmodium protein, unknown function			none		unknown function	0	0	0	na	null	null	null
PBANKA_051090	40S ribosomal protein S2B, putative			PF3D7_1026800	40S ribosomal protein S2B, putative	ribosome	0	0	0	na	structural constituent of ribosome	translation	intracellular, ribosome, small ribosomal subunit
PBANKA_051120	u3 small nucleolar ribonucleoprotein protein mpp10, putative (MPP10)			PF3D7_1027100	small subunit rRNA processing stabilizing factor, putative	RNA	0	0	0	na	null	null	null
PBANKA_051180	centrin-3, putative			PF3D7_1027700.2	centrin-3	chromatin	0	0	0	na	calcium ion binding	null	null
PBANKA_051190	60S ribosomal protein L3, putative			PF3D7_1027800	60S ribosomal protein L3, putative	ribosome	0	0	0	na	structural constituent of ribosome	translation	intracellular, ribosome
PBANKA_051270	conserved Plasmodium protein, unknown function			PF3D7_1028600	conserved Plasmodium protein, unknown function	unknown function	0	0	0	na	null	null	null
PBANKA_051280	merozoite TRAP-like protein, putative (MTRAP)			PF3D7_1028700	merozoite TRAP-like protein (MTRAP)	contaminant	0	0	0	na	null	null	null
PBANKA_051430	conserved Plasmodium protein, unknown function	-1.9	0.73	PF3D7_1030300	conserved Plasmodium protein, unknown function	unknown function	0	0	0	na	null	null	null
PBANKA_051540	conserved Plasmodium protein, unknown function			PF3D7_1031600	conserved Plasmodium protein, unknown function (GEXP15)	unknown function	0	0	0	na	null	null	null
PBANKA_051630	DER1-like protein, putative			PF3D7_1032500	DER1-like protein, putative (Der1-2)	proteasome	0	0	0	na	null	null	null
PBANKA_051660	leucine-rich repeat protein (LRR1)			PF3D7_1032800	leucine-rich repeat protein (LRR1)	signal transduction	0	0	0	na	protein binding	null	null
PBANKA_051690	S-adenosylmethionine decarboxylase, putative			PF3D7_1033100	S-adenosylmethionine decarboxylase/ornithine decarboxylase (AdoMetDC/ODC)	polyamine biosynthesis	0	0	0	na	adenosylmethionine decarboxylase activity	spermidine biosynthetic process, spermine biosynthetic process	null
PBANKA_051700	early transcribed membrane protein (ETRAMP)					early transcribed membrane protein (ETRAMP)	0	0	0	na	null	null	null
PBANKA_051740	myb2 transcription factor, putative (Myb2)			PF3D7_1033600	myb2 transcription factor, putative (Myb2)	transcription	0	0	0	na	DNA binding	null	null
PBANKA_051750	bromodomain protein, putative			PF3D7_1033700	bromodomain protein, putative	chromatin	0	0	0	na	null	null	nucleus
PBANKA_051820	flavoprotein subunit of succinate dehydrogenase, putative			PF3D7_1034400	flavoprotein subunit of succinate dehydrogenase (SDHA)	TCA, redoxmeth	0	0	0	different in fertilization, ookinete and oocyst	electron carrier activity, flavin adenine dinucleotide binding, oxidoreductase activity, acting on the CH-CH group of donors	electron transport chain, oxidation-reduction process, tricarboxylic acid cycle	null
PBANKA_051900	S-antigen, putative						0	0	0	na	null	null	null
PBANKA_051960	conserved Plasmodium protein, unknown function					unknown function	0	0	0	na	null	null	null
PBANKA_052020	ADP/ATP transporter on adenylate translocase, putative			PF3D7_1037300	ADP/ATP transporter on adenylate translocase	transporter	0	0	0	na	binding, transporter activity	transport	mitochondrial inner membrane
PBANKA_052120	conserved Plasmodium protein, unknown function			PF3D7_1038300	conserved Plasmodium protein, unknown function	RNA	0	0	0	na	null	null	null
PBANKA_052210	conserved Plasmodium protein, unknown function			PF3D7_0421700	conserved Plasmodium protein, unknown function	unknown function	0	0	0	na	null	null	null
PBANKA_052270	alpha tubulin 2			PF3D7_0422300	alpha tubulin 2	gliding motility	0	0	0	na	GTP binding, GTPase activity, structural molecule activity	microtubule-based movement, protein polymerization	microtubule, protein complex
PBANKA_052360	BSD-domain protein, putative			PF3D7_0423200	BSD domain, putative	transcription	0	0	0	na	null	null	null
PBANKA_052430	tryptophan/threonine-rich antigen, putative						0	0	0	na	null	null	null
PBANKA_060060	NIMA related kinase 3, putative (NEK3)			PF3D7_1201600	NIMA related kinase 3 (NEK3)	kinase	0	0	0	KO not successful	ATP binding, protein serine/threonine kinase activity, protein tyrosine kinase activity	protein phosphorylation	null
PBANKA_060090	conserved Plasmodium protein, unknown function			PF3D7_1201900	conserved protein, unknown function	unknown function	0	0	0	na	null	null	null
PBANKA_060160	conserved protein, unknown function			PF3D7_1202600	conserved protein, unknown function	transcription	0	0	0	na	null	null	null
PBANKA_060190	high mobility group protein, putative (HMGB1)			PF3D7_1202900	high mobility group protein (HMGB1)	chromatin	0	0	0	KO not successful; (mutated) sprorozoite and liver stage	DNA binding	null	nucleus
PBANKA_060430	conserved Plasmodium protein, unknown function			PF3D7_1205600	conserved Plasmodium protein, unknown function	unknown function	0	0	0	na	null	null	null

PBANKA_060510	Tat binding protein 1(TBP-1)-interacting protein, putative			PF3D7_1206500	Tat binding protein 1(TBP-1)-interacting protein, putative	proteasome	0	0	0	na	null	null	null
PBANKA_060530	eukaryotic translation initiation factor 5, putative			PF3D7_1206700	eukaryotic translation initiation factor 5, putative	translation	0	0	0	na	translation initiation factor activity	translational initiation	null
PBANKA_060560	conserved Plasmodium protein, unknown function	1.24	0.75	PF3D7_1207000	conserved Plasmodium protein, unknown function	unknown function	0	0	0	na	null	null	null
PBANKA_060630	conserved Plasmodium protein, unknown function			PF3D7_1207800	conserved Plasmodium protein, unknown function	unknown function	0	0	0	na	null	null	null
PBANKA_060810	conserved Plasmodium protein, unknown function			PF3D7_1209700	conserved Plasmodium protein, unknown function	unknown function	0	0	0	na	null	null	null
PBANKA_060950	kinesin-7, putative			PF3D7_1211000	kinesin-like protein, putative	dynein/kinesin	0	0	0	na	ATP binding, microtubule motor activity	microtubule-based movement	null
PBANKA_060990	heat shock protein DNAJ homologue Pfj4, putative			PF3D7_1211400	heat shock protein DNAJ homologue Pfj4 (PfJ4)	chaperone	0	0	0	na	heat shock protein binding	null	null
PBANKA_061020	minchromosome maintenance (MCM) complex subunit, putative (MCM5)			PF3D7_1211700	minchromosome maintenance (MCM) complex subunit, putative (MCM5)	DNA replication	0	0	0	na	ATP binding, DNA binding	DNA replication	null
PBANKA_061070	conserved Plasmodium protein, unknown function					unknown function	0	0	0	na	null	null	null
PBANKA_061180	N-acetyltransferase, putative			PF3D7_1437000	N-acetyltransferase, putative	chromatin modifying proteins	0	0	0	na	N-acetyltransferase activity	metabolic process	null
PBANKA_061520	calcium dependent protein kinase 4 (CDPK4)			PF3D7_0717500	calcium dependent protein kinase 4 (CDPK4)	kinase	0	0	0	different in fertilization, ookinete and oocyst	ATP binding, calcium ion binding, protein serine/threonine kinase activity, protein tyrosine kinase activity	protein phosphorylation	null
PBANKA_061550	conserved Plasmodium protein, unknown function			PF3D7_0717800	conserved Plasmodium protein, unknown function	unknown function	0	0	0	na	null	null	null
PBANKA_061670	NIMA related kinase 4 (NEK4)			PF3D7_0719200	NIMA related kinase 4 (NEK4)	kinase	0	0	0	different in fertilization and ookinete	ATP binding, protein serine/threonine kinase activity, protein tyrosine kinase activity	protein phosphorylation	null
PBANKA_061710	60S ribosomal protein L11a, putative			PF3D7_0719600	60S ribosomal protein L11a, putative	ribosome	0	0	0	na	structural constituent of ribosome	translation	intracellular, ribosome
PBANKA_061720	40S ribosomal protein S10, putative			PF3D7_0719700	40S ribosomal protein S10, putative	ribosome	0	0	0	na	null	null	null
PBANKA_061840	conserved Plasmodium protein, unknown function			PF3D7_0720900	conserved Plasmodium protein, unknown function	unknown function	0	0	0	na	null	null	null
PBANKA_061910	40S ribosomal protein S5, putative			PF3D7_0721600	40S ribosomal protein S5, putative	ribosome	0	0	0	na	structural constituent of ribosome	translation	intracellular, ribosome, small ribosomal subunit
PBANKA_061920	secreted ookinete protein, putative (PSOP1)			PF3D7_0721700	secreted ookinete protein, putative (PSOP1)	adhesin	0	0	0	not different from wild type	null	null	null
PBANKA_061930	conserved Plasmodium protein, unknown function			PF3D7_0721800	conserved Plasmodium protein, unknown function	unknown function	0	0	0	na	null	null	null
PBANKA_062000	cell cycle control protein cwf15, putative			PF3D7_0722500	cell cycle control protein cwf15, putative	mRNA	0	0	0	na	null	nuclear mRNA splicing, via spliceosome	spliceosomal complex
PBANKA_062050	tRNAHis guanylyltransferase, putative			PF3D7_0723000	tRNAHis guanylyltransferase, putative	translation	0	0	0	na	null	null	null
PBANKA_062090	conserved Plasmodium protein, unknown function	-1.81	0.73	PF3D7_0723400	conserved Plasmodium protein, unknown function	unknown function	0	0	0	na	null	null	null
PBANKA_062190	mitochondrial import inner membrane translocase subunit tim14, putative			PF3D7_0724400	mitochondrial import inner membrane translocase subunit tim14, putative	transporter	0	0	0	na	heat shock protein binding	null	null
PBANKA_062240	kinesin-19, putative	-4.93	0.75	PF3D7_0724900	kinesin-like protein, putative	dynein/kinesin	0	0	0	na	ATP binding, microtubule motor activity	microtubule-based movement	null
PBANKA_062280	conserved Plasmodium protein, unknown function			PF3D7_0725300	conserved Plasmodium protein, unknown function	unknown function	0	0	0	na	null	null	null
PBANKA_062300	Plasmodium exported protein, unknown function					unknown function	0	0	0	na	null	null	null
PBANKA_062310	conserved rodent malaria protein, unknown function					unknown function	0	0	0	KO not successful	null	null	null
PBANKA_062330	tryptophan-rich antigen, putative						0	0	0	na	null	null	null
PBANKA_070020	BIR protein					BIR protein	0	0	0	na	null	null	null
PBANKA_070110	conserved Plasmodium protein, unknown function					unknown function	0	0	0	na	null	null	null
PBANKA_070160	conserved protein, unknown function			PF3D7_0829100	conserved protein, unknown function	unknown function	0	0	0	na	null	null	null

PBANKA_070200	conserved Plasmodium protein, unknown function			PF3D7_0828700	conserved Plasmodium protein, unknown function	unknown function	0	0	0	na	null	null	null
PBANKA_070280	protein disulfide isomerase			PF3D7_0827900	protein disulfide isomerase (PDI8)	proteasome	0	0	0	na	isomerase activity	cell redox homeostasis	endoplasmic reticulum
PBANKA_070350	conserved Plasmodium protein, unknown function	-2.88	0.75	PF3D7_0827200	conserved Plasmodium protein, unknown function	unknown function	0	0	0	na	null	null	null
PBANKA_070430	SPRY domain, putative			PF3D7_0826300	SPRY domain, putative	protein-protein interaction	0	0	0	na	null	null	null
PBANKA_070470	conserved Plasmodium protein, unknown function			PF3D7_0825900	conserved Plasmodium protein, unknown function	unknown function	0	0	0	na	null	null	null
PBANKA_070540	translation initiation factor IF-3, putative			PF3D7_0825200	translation initiation factor IF-3, putative	translation	0	0	0	na	translation initiation factor activity	translational initiation	null
PBANKA_070550	conserved Plasmodium protein, unknown function			PF3D7_0825100	conserved Plasmodium protein, unknown function	unknown function	0	0	0	na	null	null	null
PBANKA_070560	conserved Plasmodium protein, unknown function	-4.04	0.75	PF3D7_0825000	conserved Plasmodium protein, unknown function	unknown function	0	0	0	na	null	null	null
PBANKA_070690	conserved Plasmodium protein, unknown function			PF3D7_0823700	conserved Plasmodium protein, unknown function	unknown function	0	0	0	na	null	null	null
PBANKA_071010	2-oxoglutarate dehydrogenase e1, putative			PF3D7_0820700	2-oxoglutarate dehydrogenase E1 component	TCA	0	0	0	na	oxoglutarate dehydrogenase (succinyl-transferring) activity, thiamine pyrophosphate binding	glycolysis	null
PBANKA_071120	conserved Plasmodium protein, unknown function			PF3D7_0819600	conserved Plasmodium protein, unknown function	unknown function	0	0	0	na	null	null	null
PBANKA_071190	heat shock protein, putative (HSP70)			PF3D7_0818900	heat shock protein 70 (Hsp70)	transporter	0	0	0	na	ATP binding	null	null
PBANKA_071230	zinc finger protein, putative			PF3D7_0818500	zinc finger protein, putative	proteasome	0	0	0	na	DNA binding, zinc ion binding	null	null
PBANKA_071260	14-3-3 protein, putative			PF3D7_0818200	14-3-3 protein, putative		0	0	0	na	protein domain specific binding	null	cytoplasm, nucleus
PBANKA_071440	conserved Plasmodium protein, unknown function			PF3D7_0816400	conserved Plasmodium protein, unknown function	kinase	0	0	0	na	calcium ion binding	null	null
PBANKA_071550	phosphoglucomutase-2 (PGM2)			PF3D7_0413500	phosphoglucomutase-2 (PGM2)	glycolysis	0	0	0	KO not successful; (tagged) fertilization and ookinete	null	null	cytoplasm
PBANKA_071560	26S proteasome AAA-ATPase subunit RPT3, putative			PF3D7_0413600	26S proteasome AAA-ATPase subunit RPT3, putative	proteasome	0	0	0	na	ATP binding, nucleoside-triphosphatase activity	protein catabolic process	cytoplasm, nucleus
PBANKA_071570	lysine decarboxylase-like protein, putative			PF3D7_0413700	lysine decarboxylase-like protein, putative	AA metabolism	0	0	0	na	null	null	null
PBANKA_071600	chromosome associated protein, putative			PF3D7_0414000	chromosome associated protein, putative	chromatin	0	0	0	na	ATP binding, protein binding	chromosome organization	chromosome
PBANKA_071660	conserved Plasmodium protein, unknown function			PF3D7_0414600	conserved protein, unknown function	unknown function	0	0	0	na	null	null	null
PBANKA_071780	60S ribosomal protein L15, putative			PF3D7_0415900	60S ribosomal protein L15, putative	ribosome	0	0	0	na	structural constituent of ribosome	translation	intracellular, ribosome
PBANKA_071970	conserved Plasmodium protein, unknown function			PF3D7_0417600	conserved Plasmodium protein, unknown function	unknown function	0	0	0	na	null	null	null
PBANKA_072040	conserved Plasmodium protein, unknown function			PF3D7_0418300	conserved Plasmodium protein, unknown function	unknown function	0	0	0	na	null	null	null
PBANKA_072130	CGI-141 protein homolog, putative			PF3D7_0419200	CGI-141 protein homolog, putative	trafficking	0	0	0	na	null	vesicle-mediated transport	null
PBANKA_072170	ran binding protein 1, putative			PF3D7_0419600	ran binding protein 1, putative	mRNA	0	0	0	na	null	intracellular transport	null
PBANKA_080180	conserved Plasmodium protein, unknown function			PF3D7_0704100	conserved Plasmodium membrane protein, unknown function	unknown function	0	0	0	na	null	null	null
PBANKA_080230	e3 ubiquitin-protein ligase, putative			PF3D7_0704600	ubiquitin transferase, putative	proteasome	0	0	0	na	acid-amino acid ligase activity	cellular protein modification process	intracellular
PBANKA_080310	minchromosome maintenance (MCM) complex subunit, putative (MCM7)			PF3D7_0705400	minchromosome maintenance (MCM) complex subunit, putative (MCM7)	DNA replication	0	0	0	na	ATP binding, DNA binding, nucleoside-triphosphatase activity	DNA replication, DNA-dependent DNA replication initiation	nucleus
PBANKA_080340	40S ribosomal protein S29, putative			PF3D7_0705700	40S ribosomal protein S29, putative	ribosome	0	0	0	na	structural constituent of ribosome	translation	intracellular, ribosome
PBANKA_080400	ribosomal protein, L37e, putative			PF3D7_0706400	ribosomal protein, L37e, putative	ribosome	0	0	0	na	structural constituent of ribosome	translation	intracellular, ribosome
PBANKA_080410	conserved Plasmodium protein, unknown function			PF3D7_0706500	conserved Plasmodium protein, unknown function	unknown function	0	0	0	na	null	null	null

PBANKA_080570	heat shock protein 90, putative (HSP90)	PF3D7_0708400	heat shock protein 90 (HSP90)	chaperone	0	0	0	na	ATP binding, unfolded protein binding	protein folding, response to stress	null
PBANKA_080590	kinesin, putative	PF3D7_0319400	kinesin, putative	dynein/kinesin	0	0	0	na	ATP binding, microtubule motor activity	microtubule-based movement	null
PBANKA_080680	conserved Plasmodium protein, unknown function	PF3D7_0318400	conserved Plasmodium protein, unknown function	unknown function	0	0	0	na	null	null	null
PBANKA_080700	dna-directed rna polymerase ii subunit rpb1, putative (RPB1)	PF3D7_0318200	DNA-directed RNA polymerase II, putative	transcription	0	0	0	na	DNA binding, DNA-directed RNA polymerase activity	transcription from RNA polymerase II promoter	DNA-directed RNA polymerase II, core complex
PBANKA_080760	40S ribosomal protein S11, putative	PF3D7_0317600	40S ribosomal protein S11, putative	ribosome	0	0	0	na	structural constituent of ribosome	translation	intracellular, ribosome
PBANKA_080990	tRNA pseudouridine synthase, putative	PF3D7_0908700	tRNA pseudouridine synthase, putative	translation	0	0	0	na	RNA binding, pseudouridine synthase activity	pseudouridine synthesis	null
PBANKA_081050	apoptosis-related protein, putative	PF3D7_0909300	apoptosis-related protein, putative (ARP)	apoptosis	0	0	0	na	DNA binding	null	null
PBANKA_081300	inhibitor of cysteine proteases (ICP)	PF3D7_0911900	falstatin (ICP)	protease	0	0	0	KO not successful	null	null	null
PBANKA_081340	conserved Plasmodium protein, unknown function	PF3D7_0912400	conserved Plasmodium protein, unknown function	Ca2+ binding	0	0	0	na	null	null	null
PBANKA_081350	conserved Plasmodium protein, unknown function	PF3D7_0912500	conserved Plasmodium protein, unknown function	unknown function	0	0	0	na	nucleic acid binding	null	nucleus
PBANKA_081480	conserved Plasmodium protein, unknown function	PF3D7_0913800	conserved Plasmodium protein, unknown function	unknown function	0	0	0	na	null	null	null
PBANKA_081690	conserved Plasmodium protein, unknown function	PF3D7_0915900	conserved Plasmodium protein, unknown function	unknown function	0	0	0	na	null	null	null
PBANKA_081700	sugar transporter, putative	PF3D7_0916000	sugar transporter, putative	transporter	0	0	0	na	null	null	null
PBANKA_081890	heat shock protein 70, putative	PF3D7_0917900	heat shock protein 70 (Hsp70-2)	chaperone	0	0	0	na	ATP binding	null	null
PBANKA_081990	nucleosome assembly protein (NAPS)	PF3D7_0919000	nucleosome assembly protein (NAPS)	chromatin	0	0	0	KO not successful; (tagged) different in fertilization, ookinete and oocyst	null	nucleosome assembly	nuclear periphery, nucleus
PBANKA_082110	CS domain protein, putative	PF3D7_0920200	CS-domain containing protein, conserved in Apicomplexa	adhesin	0	0	0	na	null	null	null
PBANKA_082170	inosine-5'-monophosphate dehydrogenase, putative	PF3D7_0920800	inosine-5'-monophosphate dehydrogenase	purine metabolism	0	0	0	na	IMP dehydrogenase activity	oxidation-reduction process	null
PBANKA_082200	conserved Plasmodium protein, unknown function	PF3D7_0921100	conserved Plasmodium protein, unknown function	unknown function	0	0	0	na	null	null	null
PBANKA_082360	pre-mRNA splicing factor, putative	PF3D7_0922700	pre-mRNA splicing factor, putative	mRNA	0	0	0	na	null	RNA splicing	spliceosomal complex
PBANKA_082480	RNA-binding protein, putative	PF3D7_0923900	RNA binding protein, putative	mRNA	0	0	0	na	nucleic acid binding	null	null
PBANKA_082560	splicing factor 3A subunit 3, putative (SF3A3)	PF3D7_0924700	splicing factor 3A subunit 3, putative (SF3A3)	mRNA	0	0	0	na	nucleic acid binding, zinc ion binding	null	nucleus
PBANKA_082580	conserved Plasmodium protein, unknown function	PF3D7_0925000	conserved Plasmodium protein, unknown function	unknown function	0	0	0	na	null	null	null
PBANKA_082640	zinc-binding protein (Yippee), putative	PF3D7_0925600	zinc binding protein (Yippee), putative	senescence, apoptosis	0	0	0	na	null	null	null
PBANKA_082670	conserved Plasmodium protein, unknown function	PF3D7_0925900	conserved Plasmodium protein, unknown function	unknown function	0	0	0	na	null	null	null
PBANKA_082830	dynein light chain, putative	PF3D7_0927500	dynein light chain, putative	dynein/kinesin	0	0	0	na	null	null	null
PBANKA_082890	conserved Plasmodium protein, unknown function	PF3D7_0928100	conserved Plasmodium protein, unknown function	unknown function	0	0	0	na	null	null	null
PBANKA_083030	conserved Plasmodium protein, unknown function	PF3D7_0929500	conserved Plasmodium protein, unknown function	unknown function	0	0	0	na	null	null	null
PBANKA_083100	merozoite surface protein 1 (MSP1)	PF3D7_0930300	merozoite surface protein 1 (MSP1)	contaminant	0	0	0	KO not successful; (mutated) different in sporozoite and liver stage	null	pathogenesis	membrane
PBANKA_083170	NifU-like protein, putative	PF3D7_0930900	NifU-like protein, putative	apicoplast	0	0	0	na	iron ion binding, iron-sulfur cluster binding	iron-sulfur cluster assembly	null
PBANKA_083260	proteasome subunit beta type-6, putative	PF3D7_0931800	proteasome precursor, putative	proteasome	0	0	0	na	threonine-type endopeptidase activity	proteolysis involved in cellular protein catabolic process	proteasome core complex
PBANKA_083310	aspartyl aminopeptidase, putative	PF3D7_0932300	M18 aspartyl aminopeptidase (M18AAP)	protease	0	0	0	na	aminopeptidase activity, zinc ion binding	proteolysis	vacuole

PBANKA_083520	transcription factor with AP2 domain(s), putative			PF3D7_0934400	transcription factor with AP2 domain(s), putative (ApiAP2)	transcription	0	0	0	na	sequence-specific DNA binding transcription factor activity	regulation of transcription, DNA-dependent	null
PBANKA_083700	BIR protein					BIR protein	0	0	0	na	null	null	null
PBANKA_090140	mitochondrial ATP synthase delta subunit, putative			PF3D7_1147700	mitochondrial ATP synthase delta subunit, putative	ATP synthase complex	0	0	0	na	hydrogen ion transporting ATP synthase activity, rotational mechanism, proton-transporting ATPase activity, rotational mechanism	ATP synthesis coupled proton transport	mitochondrion, proton-transporting ATP synthase complex, catalytic core F(1)
PBANKA_090190	tubulin-tyrosine ligase, putative	-3.79	0.75	PF3D7_1147200	tubulin-tyrosine ligase, putative	microtubule	0	0	0	na	tubulin-tyrosine ligase activity	cellular protein modification process	null
PBANKA_090260	CCAAT-box DNA binding protein subunit B, putative			PF3D7_1146600	CCAAT-box DNA binding protein subunit B	chromatin	0	0	0	na	sequence-specific DNA binding	null	intracellular, nucleus
PBANKA_090370	conserved Plasmodium protein, unknown function			PF3D7_1145300	conserved Plasmodium protein, unknown function	unknown function	0	0	0	na	null	null	null
PBANKA_090420	conserved Plasmodium protein, unknown function	-3.03	0.75	PF3D7_1144800	conserved Plasmodium protein, unknown function	unknown function	0	0	0	na	null	null	null
PBANKA_090460	conserved Plasmodium protein, unknown function			PF3D7_1144400	conserved Plasmodium protein, unknown function	unknown function	0	0	0	na	null	null	null
PBANKA_090470	60S ribosomal protein L41, putative			PF3D7_1144300	60S ribosomal protein L41, putative	ribosome	0	0	0	na	structural constituent of ribosome	translation	ribosome
PBANKA_090500	40S ribosomal protein S21e, putative			PF3D7_1144000	40S ribosomal protein S21e, putative	ribosome	0	0	0	na	structural constituent of ribosome	translation	intracellular, ribosome
PBANKA_090560	translation initiation factor eIF-1A, putative			PF3D7_1143400	translation initiation factor eIF-1A, putative	translation	0	0	0	na	RNA binding, translation initiation factor activity	translational initiation	null
PBANKA_090730	conserved Plasmodium protein, unknown function			PF3D7_1141700	conserved Plasmodium protein, unknown function	protease	0	0	0	na	null	null	null
PBANKA_090790	conserved Plasmodium protein, unknown function	-6.19	0.75			unknown function	0	0	0	na	null	null	null
PBANKA_090870	conserved Plasmodium protein, unknown function			PF3D7_1140300	P-loop containing nucleoside triphosphate hydrolase, putative	purine metabolism	0	0	0	na	null	null	null
PBANKA_090880	conserved Plasmodium protein, unknown function			PF3D7_1140200	conserved Plasmodium protein, unknown function	unknown function	0	0	0	na	null	null	null
PBANKA_090980	conserved Plasmodium protein, unknown function			PF3D7_1139100	conserved Plasmodium protein, unknown function	RNA binding	0	0	0	na	nucleic acid binding	null	null
PBANKA_091020	protein phosphatase 2C, putative			PF3D7_1138500	protein phosphatase 2c	phosphatase	0	0	0	na	catalytic activity	null	null
PBANKA_091090	conserved Plasmodium protein, unknown function			PF3D7_1137800	conserved Plasmodium protein, unknown function	unknown function	0	0	0	na	null	null	null
PBANKA_091170	subtilisin-like protease 2 (SUB2)			PF3D7_1136900	subtilisin-like protease 2 (SUB2)	protease	0	0	0	KO not successful	serine-type endopeptidase activity	proteolysis	null
PBANKA_091200	conserved Plasmodium protein, unknown function			PF3D7_1136600	conserved Plasmodium protein, unknown function	unknown function	0	0	0	na	null	null	null
PBANKA_091250	conserved Plasmodium protein, unknown function	-3.34	0.75	PF3D7_1136100	conserved Plasmodium protein, unknown function	unknown function	0	0	0	na	null	null	null
PBANKA_091260	conserved Plasmodium protein, unknown function			PF3D7_1107300	polyadenylate-binding protein-interacting protein 1, putative (PAIP1)	mRNA	0	0	0	na	null	null	null
PBANKA_091440	heat shock protein hsp70 homologue, putative (UIS24)			PF3D7_1134000	heat shock protein 70 (Hsp70-3)	transporter	0	0	0	na	ATP binding, unfolded protein binding	protein folding	null
PBANKA_091500	apical membrane antigen 1 (AMA1)			PF3D7_1133400	apical membrane antigen 1 (AMA1)	contaminant	0	0	0	KO not successful; (mutated) sporozoite and liver stage	null	pathogenesis	membrane
PBANKA_091540	conserved Plasmodium protein, unknown function			PF3D7_1133000	conserved Plasmodium protein, unknown function	unknown function	0	0	0	na	null	null	null
PBANKA_091560	aquaglyceroporin (AQP)			PF3D7_1132800	aquaglyceroporin (AQP)	glycolysis	0	0	0	not different from wild type	transporter activity	transport	membrane, perinuclear region of cytoplasm
PBANKA_091720	RNA-binding protein s1, putative	-1.7	0.7			mRNA	0	0	0	na	nucleic acid binding	null	null
PBANKA_091800	60S ribosomal protein, putative			PF3D7_1130200	60S ribosomal protein P0	ribosome	0	0	0	na	structural constituent of ribosome	ribosome biogenesis, translational elongation	intracellular, ribosome
PBANKA_091830	transporter, putative			PF3D7_1129900	transporter, putative	transporter	0	0	0	na	null	null	null
PBANKA_091860	phosphatidylinositol-4-phosphate 5-kinase, putative			PF3D7_1129600	phosphatidylinositol-4-phosphate-5-kinase, putative	inositol phosphate metabolism	0	0	0	na	null	null	null
PBANKA_091910	parasitophorous vacuolar protein 1, putative (PV1)			PF3D7_1129100	parasitophorous vacuolar protein 1 (PV1)	PV membrane protein	0	0	0	na	null	null	null

PBANKA_092080	CRAL/TRIO domain-containing protein, putative	PF3D7_1127600	CRAL/TRIO domain-containing protein, putative	trafficking	0	0	0	na	null	null	null
PBANKA_092170	conserved Plasmodium protein, unknown function	PF3D7_1126700	conserved Plasmodium protein, unknown function	unknown function	0	0	0	na	null	null	null
PBANKA_092280	small nuclear ribonucleoprotein D1, putative (SNRPD1)	PF3D7_1125500	small nuclear ribonucleoprotein D1, putative (SNRPD1)	mRNA	0	0	0	na	null	null	null
PBANKA_092340	60S ribosomal protein L35, putative	PF3D7_1124900	60S ribosomal protein L35, putative	ribosome	0	0	0	na	structural constituent of ribosome	translation	intracellular, ribosome
PBANKA_092490	translation elongation factor EF-1, subunit alpha, putative	PF3D7_1123400	translation elongation factor EF-1, subunit alpha, putative	translation	0	0	0	na	GTP binding, GTPase activity	null	null
PBANKA_092550	calcium dependent protein kinase 6 (CDPK6)	PF3D7_1122800	calcium dependent protein kinase 6 (CDPK6)	kinase	0	0	0	different in sporozoite and liver stage	ATP binding, calcium ion binding, protein serine/threonine kinase activity, protein tyrosine kinase activity	protein phosphorylation	null
PBANKA_092670	circumsporozoite-related antigen	PF3D7_1121600	circumsporozoite-related antigen (CRA)	adhesin	0	0	0	different in liver stage	null	null	null
PBANKA_092750	conserved Plasmodium protein, unknown function	PF3D7_1120800	conserved Plasmodium protein, unknown function	unknown function	0	0	0	na	null	null	null
PBANKA_092800	conserved Plasmodium protein, unknown function	PF3D7_1120200	conserved Plasmodium protein, unknown function	unknown function	0	0	0	na	null	null	null
PBANKA_092810	phosphoglycerate mutase, putative (PGM1)	PF3D7_1120100	phosphoglycerate mutase, putative (PGM1)	glycolysis	0	0	0	na	intramolecular transferase activity, phosphotransferases	glycolysis	null
PBANKA_092920	conserved Plasmodium protein, unknown function	PF3D7_1118900	conserved Plasmodium protein, unknown function	unknown function	0	0	0	na	null	null	null
PBANKA_093090	deubiquinating/deneddylating enzyme, putative (UCH54)	PF3D7_1117100	deubiquinating/deneddylating enzyme (UCH54)	proteasome	0	0	0	na	ubiquitin thiolesterase activity	ubiquitin-dependent protein catabolic process	intracellular
PBANKA_093120	heat shock protein 101 (ClpB2)	PF3D7_1116800	heat shock protein 101 (HSP101)	chaperone	0	0	0	KO not successful; (tagged) different in sporozoite and liver stage	ATP binding, nucleoside-triphosphatase activity, protein binding	protein metabolic process	null
PBANKA_093130	dipeptidyl aminopeptidase, putative	PF3D7_1116700	cathepsin C, homolog,dipeptidyl peptidase 1 (DPAP1)	protease	0	0	0	na	cysteine-type peptidase activity	proteolysis	null
PBANKA_093210	conserved Plasmodium protein, unknown function	PF3D7_1115900	conserved Plasmodium membrane protein, unknown function	unknown function	0	0	0	na	zinc ion binding	null	null
PBANKA_093300	serine/threonine protein kinase, putative	PF3D7_1114700	serine/threonine protein kinase, putative (CLK3) (PRK4)	kinase	0	0	0	KO not successful	ATP binding, protein serine/threonine kinase activity, protein tyrosine kinase activity	protein phosphorylation	null
PBANKA_093390	glyoxalase I, putative	PF3D7_1147100	conserved Plasmodium protein, unknown function	unknown function	0	0	0	na	null	null	null
PBANKA_093720	asparagine-rich antigen, putative	PF3D7_1111600	endonuclease/exonuclease/phosphatase family protein, putative	inositol phosphate metabolism	0	0	0	na	nucleic acid binding	null	null
PBANKA_093730	conserved Plasmodium protein, unknown function	PF3D7_1110400	asparagine-rich antigen		0	0	0	na	nucleic acid binding	null	intracellular
PBANKA_093770	apicoplast ribosomal protein L36e precursor, putative	PF3D7_1109900	apicoplast ribosomal protein L36e precursor, putative	ribosome apico	0	0	0	na	structural constituent of ribosome	translation	intracellular, ribosome
PBANKA_093840	endoplasmic reticulum-resident calcium binding protein, putative	PF3D7_1108600	endoplasmic reticulum-resident calcium binding protein (ERC)	HG ERG	0	0	0	na	calcium ion binding	null	null
PBANKA_093880	conserved Plasmodium protein, unknown function	PF3D7_1108100	conserved Plasmodium protein, unknown function	unknown function	0	0	0	na	null	null	null
PBANKA_094080	apicoplast ribosomal protein S15 precursor, putative	PF3D7_1106100	apicoplast ribosomal protein S15 precursor, putative	ribosome apico	0	0	0	na	structural constituent of ribosome	translation	intracellular, ribosome
PBANKA_094110	conserved Plasmodium protein, unknown function	PF3D7_1105800	conserved Plasmodium protein, unknown function	unknown function	0	0	0	na	null	null	null
PBANKA_094190	histone H4, putative	PF3D7_1105000	histone H4 (H4)	chromatin	0	0	0	na	DNA binding	DNA-dependent transcription, initiation, nucleosome assembly	nucleosome, nucleus
PBANKA_094250	conserved Plasmodium protein, unknown function	PF3D7_1104400	conserved protein, unknown function	nucleic acid binding	0	0	0	na	null	null	null
PBANKA_094270	chromatin remodeling protein, putative (SNF2L)	PF3D7_1104200	chromatin remodeling protein (SNF2L)	chromatin	0	0	0	na	ATP binding, DNA binding, helicase activity	null	null
PBANKA_094360	60S acidic ribosomal protein, putative	PF3D7_1103100	60S ribosomal protein P1, putative	ribosome	0	0	0	na	structural constituent of ribosome	translational elongation	intracellular, ribosome
PBANKA_100060	erythrocyte membrane antigen 1	PF3D7_0403700	CGI-201 protein, short form	mRNA	0	0	0	na	null	null	null
PBANKA_100140	splicing factor, putative	PF3D7_0403800	conserved Plasmodium protein, unknown function	mRNA	0	0	0	na	null	RNA processing	intracellular
PBANKA_100150	conserved Plasmodium protein, unknown function	PF3D7_0404000	conserved Plasmodium protein, unknown function	unknown function	0	0	0	na	null	null	null

PBANKA_100200	conserved Plasmodium protein, unknown function	PF3D7_0404300	conserved Plasmodium protein, unknown function	unknown function	0	0	0	na	null	intracellular transport	null
PBANKA_100340	lysine decarboxylase, putative (UIS14)	PF3D7_0405700	lysine decarboxylase, putative	AA metabolism	0	0	0	na	catalytic activity	null	null
PBANKA_100380	vacuolar ATP synthase subunit b, putative	PF3D7_0406100	vacuolar ATP synthase subunit b	vacuolar ATP synthase	0	0	0	na	hydrogen ion transporting ATP synthase activity, rotational mechanism, proton-transporting ATPase activity, rotational mechanism	ATP synthesis coupled proton transport	proton-transporting V-type ATPase, V1 domain, proton-transporting two-sector ATPase complex
PBANKA_100610	flap endonuclease 1, putative	PF3D7_0408500	flap endonuclease 1 (FEN1)	DNA replication, DNA repair	0	0	0	na	5'-3' exonuclease activity, DNA binding	DNA repair	null
PBANKA_100670	U4/U6 small nuclear ribonucleoprotein PRP31, putative (PRPF31)	PF3D7_0409100	U4/U6 small nuclear ribonucleoprotein PRP31, putative (PRPF31)	mRNA	0	0	0	na	null	null	null
PBANKA_100720	replication protein A1, large subunit, putative (RPA1)	PF3D7_0409600	replication protein A1, large subunit (RPA1)	DNA repair	0	0	0	na	nucleic acid binding	null	null
PBANKA_100750	actin-like protein, putative	PF3D7_0409900	actin-like protein, putative	gliding motility	0	0	0	na	protein binding	null	null
PBANKA_100770	protein phosphatase 2C, putative	PF3D7_0410500	conserved Plasmodium protein, unknown function	unknown function	0	0	0	na	catalytic activity	null	null
PBANKA_100850	translocon component PTEX150 (PTEX150)	PF3D7_1436000	glucose-6-phosphate isomerase (GPI)	glycolysis	0	0	0	KO not successful; (tagged) sporozoite and liver stage	null	null	null
PBANKA_100880	glucose-6-phosphate isomerase, putative	PF3D7_1435700.1	conserved Plasmodium protein, unknown function	proteasome	0	0	0	na	glucose-6-phosphate isomerase activity	gluconeogenesis, glycolysis	null
PBANKA_100910	conserved Plasmodium protein, unknown function	PF3D7_1435700.2	conserved Plasmodium protein, unknown function	unknown function	0	0	0	na	null	null	null
PBANKA_100950	NAD(P)H-dependent glutamate synthase, putative	PF3D7_1435300	NAD(P)H-dependent glutamate synthase, putative	AA metabolism	0	0	0	na	electron carrier activity, glutamate synthase activity, iron-sulfur cluster binding, oxidoreductase activity, acting on the CH-NH2 group of donors, NAD or NADP as acceptor	glutamate biosynthetic process, nitrogen compound metabolic process, oxidation-reduction process	null
PBANKA_100990	conserved Plasmodium protein, unknown function	PF3D7_1434900	conserved Plasmodium protein, unknown function	unknown function	0	0	0	na	null	null	null
PBANKA_101000	conserved Plasmodium protein, unknown function	PF3D7_1434800	conserved protein, unknown function	unknown function	0	0	0	na	null	null	mitochondrial matrix
PBANKA_101050	Hsp70/Hsp90 organizing protein, putative (HOP)	PF3D7_1434300	Hsp70/Hsp90 organizing protein (HOP)	chaperone	0	0	0	na	null	null	cytosol
PBANKA_101190	h/aca ribonucleoprotein complex subunit 3, putative (NOP10)	PF3D7_1433000	ribosome biogenesis protein, NOP10-like	ribosome	0	0	0	na	null	null	null
PBANKA_101310	60S ribosomal protein L14, putative	PF3D7_1431700	60S ribosomal protein L14, putative	ribosome	0	0	0	na	structural constituent of ribosome	translation	intracellular, ribosome
PBANKA_101320	ATP-specific succinyl-CoA synthetase beta subunit, putative	PF3D7_1431600	ATP-specific succinyl-CoA synthetase beta subunit, putative	TCA	0	0	0	na	catalytic activity	metabolic process	null
PBANKA_101400	NADP-specific glutamate dehydrogenase, putative (GDH2)	PF3D7_1430700	glutamate dehydrogenase, putative (GDHb)	nucleic acid binding	0	0	0	na	binding, glutamate dehydrogenase (NADP+) activity	glutamate biosynthetic process, oxidation-reduction process	apicoplast
PBANKA_101560	apicoplast ribosomal protein L15 precursor, putative		apicoplast ribosomal protein L15 precursor, putative	ribosome apico	0	0	0	na	structural constituent of ribosome	translation	intracellular, large ribosomal subunit, ribosome
PBANKA_101630	proliferation-associated protein 2g4, putative	PF3D7_1428300	proliferation-associated protein 2g4, putative	protease	0	0	0	na	null	cellular process	null
PBANKA_101860	60S ribosomal protein L21e, putative	PF3D7_1426000	60S ribosomal protein L21e, putative	ribosome	0	0	0	na	structural constituent of ribosome	translation	intracellular, ribosome
PBANKA_101880	conserved Plasmodium protein, unknown function			unknown function	0	0	0	na	null	null	null
PBANKA_101940	60S ribosomal protein L7-3, putative	PF3D7_1424400	60S ribosomal protein L7-3, putative	ribosome	0	0	0	na	null	null	null
PBANKA_101950	60S ribosomal protein L5, putative	PF3D7_1424100	60S ribosomal protein L5, putative	ribosome	0	0	0	na	5S rRNA binding, structural constituent of ribosome	translation	intracellular, ribosome
PBANKA_102000	conserved Plasmodium protein, unknown function	PF3D7_1423400	conserved Plasmodium membrane protein, unknown function	unknown function	0	0	0	na	null	null	null
PBANKA_102070	conserved Plasmodium protein, unknown function	PF3D7_1422700	conserved Plasmodium protein, unknown function	unknown function	0	0	0	na	null	null	null
PBANKA_102110	DnaJ protein, putative	PF3D7_1422300	DnaJ protein, putative	chaperone	0	0	0	na	heat shock protein binding	null	null
PBANKA_102180	mitochondrial protein import protein TIM13, putative	PF3D7_1421500	mitochondrial protein import protein TIM13, putative	transporter	0	0	0	na	null	protein import into mitochondrial inner membrane	mitochondrial intermembrane space protein transporter complex
PBANKA_102200	40S ribosomal protein S25, putative	PF3D7_1421200	40S ribosomal protein S25, putative	ribosome	0	0	0	na	null	null	null
PBANKA_102350	conserved Plasmodium protein, unknown function	PF3D7_1419700	conserved Plasmodium protein, unknown function	translation	0	0	0	na	null	null	null

PBANKA_102470	ubiquitin fusion degradation protein UFD1, putative	PF3D7_1418000	ubiquitin fusion degradation protein UFD1, putative (UFD1)	proteasome	0	0	0	na	null	ubiquitin-dependent protein catabolic process	null
PBANKA_102490	minchromosome maintenance (MCM) complex subunit, putative (MCM2)	PF3D7_1417800	minchromosome maintenance (MCM) complex subunit (MCM2)	DNA replication	0	0	0	na	ATP binding, DNA binding, nucleoside-triphosphatase activity	DNA replication, DNA-dependent DNA replication initiation	nucleus
PBANKA_102550	NOT family protein, putative	PF3D7_1417200	NOT family protein, putative	mRNA	0	0	0	na	null	null	null
PBANKA_102830	serine/threonine protein phosphatase, putative	PF3D7_1414400	serine/threonine protein phosphatase (PP1)	phosphatase	0	0	0	na	hydrolase activity	null	null
PBANKA_102840	60S ribosomal protein L10, putative	PF3D7_1414300	60S ribosomal protein L10, putative	ribosome	0	0	0	na	structural constituent of ribosome	translation	intracellular, ribosome
PBANKA_102940	conserved Plasmodium protein, unknown function	PF3D7_1413200	conserved Plasmodium protein, unknown function	unknown function	0	0	0	na	null	null	null
PBANKA_103010	actin II	PF3D7_1412500	actin II (ACT2)	gliding motility	0	0	0	different in fertilization, ookinete, oocyst and sporozoite	protein binding	null	cytoplasm
PBANKA_103060	conserved Plasmodium protein, unknown function	PF3D7_1412000	p1/s1 nuclease, putative	DNA repair	0	0	0	na	hydrolase activity, acting on ester bonds	null	null
PBANKA_103160	conserved Plasmodium protein, unknown function	PF3D7_1410900	conserved Plasmodium protein, unknown function	transcription	0	0	0	na	null	null	null
PBANKA_103310	conserved Plasmodium protein, unknown function	PF3D7_1409200.1	conserved Plasmodium protein, unknown function	unknown function	0	0	0	na	null	null	null
PBANKA_103370	conserved Plasmodium protein, unknown function	PF3D7_1408800	conserved Plasmodium protein, unknown function	unknown function	0	0	0	na	null	null	null
PBANKA_103390	40S ribosomal protein S8e, putative	PF3D7_1408600	40S ribosomal protein S8e, putative	ribosome	0	0	0	na	null	null	intracellular
PBANKA_103430	transcription factor with AP2 domain(s) (API2-G2)	PF3D7_1408200	transcription factor with AP2 domain(s) (ApiAP2)	transcription	0	0	0	different in fertilization and ookinete	sequence-specific DNA binding transcription factor activity	regulation of transcription, DNA-dependent	null
PBANKA_103440	plasmepsin IV (PM4)	PF3D7_1407800	plasmepsin IV (PM4)	plasmepsin, protease	0	0	0	na	aspartic-type endopeptidase activity	proteolysis	null
PBANKA_103490	pre-mRNA splicing factor, putative	PF3D7_1407300	pre-mRNA splicing factor, putative	mRNA	0	0	0	na	null	null	null
PBANKA_103510	fibrillarin, putative (NOP1)	PF3D7_1407100	fibrillarin, putative (NOP1)	ribosome assembly	0	0	0	na	RNA binding	rRNA processing	nucleus
PBANKA_103520	LCCL domain-containing protein (CCp3)	PF3D7_1407000	LCCL domain-containing protein (CCp3)	adhesin	0	0	0	different in oocyst and sporozoite; (tagged) also in fertilization and ookinete	scavenger receptor activity	null	membrane
PBANKA_103630	RNA-binding protein, putative	PF3D7_1405900	RNA binding protein, putative	mRNA	0	0	0	na	nucleic acid binding	null	null
PBANKA_103760	rrna biogenesis protein rrp5, putative (RRP5)	PF3D7_1404500	U3 small nucleolar ribonucleoprotein, U3 snoRNP, putative	mRNA	0	0	0	na	null	RNA processing	intracellular
PBANKA_103800	cytochrome c, putative	PF3D7_1404100	cytochrome c, putative	redoxmeth	0	0	0	na	electron carrier activity, heme binding, iron ion binding	null	null
PBANKA_103860	conserved Plasmodium protein, unknown function	PF3D7_1403400	conserved Plasmodium protein, unknown function	unknown function	0	0	0	na	null	null	null
PBANKA_103940	ubiquitin-40s ribosomal protein s27a, putative	PF3D7_1402500	40S ribosomal protein S31/UBI, putative	ribosome	0	0	0	na	structural constituent of ribosome	translation	intracellular, ribosome
PBANKA_104020	BIR protein			BIR protein	0	0	0	na	null	null	null
PBANKA_110110	Plasmodium exported protein, unknown function			unknown function	0	0	0	na	null	null	null
PBANKA_110130	conserved rodent malaria protein, unknown function			unknown function	0	0	0	na	null	null	null
PBANKA_110230	conserved Plasmodium protein, unknown function	PF3D7_0502600	conserved Plasmodium protein, unknown function	unknown function	0	0	0	na	null	null	null
PBANKA_110340	60S ribosomal protein L31, putative	PF3D7_0503800	60S ribosomal protein L31, putative	ribosome	0	0	0	na	structural constituent of ribosome	translation	intracellular, ribosome
PBANKA_110390	conserved Plasmodium protein, unknown function	PF3D7_0504300	conserved Plasmodium protein, unknown function	unknown function	0	0	0	na	null	null	null
PBANKA_110550	conserved Plasmodium protein, unknown function	PF3D7_0505900	conserved Plasmodium protein, unknown function	unknown function	0	0	0	na	null	null	null
PBANKA_110670	60S ribosomal protein L4, putative	PF3D7_0507100	60S ribosomal protein L4, putative	ribosome	0	0	0	na	structural constituent of ribosome	translation	intracellular, ribosome
PBANKA_110680	subtilisin-like protease 3, putative (SUB3)	PF3D7_0507200	subtilisin-like protease 3, putative (SUB3)	protease	0	0	0	na	serine-type endopeptidase activity	proteolysis	null

PBANKA_110710	subtilisin-like protease 1, putative (SUB1)			PF3D7_0507500	subtilisin-like protease 1 (SUB1)	protease	0	0	0	KO not successful; (mutated) different in liver stage	serine-type endopeptidase activity	proteolysis	null
PBANKA_110800	transcription factor IIb, putative			PF3D7_0508400	transcription factor IIb, putative	transcription	0	0	0	na	null	null	null
PBANKA_110820	conserved Plasmodium protein, unknown function			PF3D7_0508600	conserved Plasmodium protein, unknown function	nucleic acid binding	0	0	0	na	null	null	null
PBANKA_110850	conserved Plasmodium protein, unknown function			PF3D7_0508900	conserved Plasmodium protein, unknown function	unknown function	0	0	0	na	null	null	null
PBANKA_110890	conserved Plasmodium protein, unknown function			PF3D7_0509300	conserved Plasmodium protein, unknown function	unknown function	0	0	0	na	null	null	null
PBANKA_111040	conserved Plasmodium protein, unknown function			PF3D7_0510800	conserved Plasmodium protein, unknown function	transcription	0	0	0	na	null	null	null
PBANKA_111130	kinase binding protein CGI-121, putative						0	0	0	na	null	null	null
PBANKA_111170	conserved Plasmodium protein, unknown function			PF3D7_0512100	conserved Plasmodium protein, unknown function	unknown function	0	0	0	na	null	null	null
PBANKA_111230	Rab1b, putative			PF3D7_0512600	Rab GTPase 1b (Rab1b)	trafficking	0	0	0	na	GTP binding, GTPase activity, protein binding	intracellular protein transport, nucleocytoplasmic transport, small GTPase mediated signal transduction	intracellular
PBANKA_111300	purine nucleotide phosphorylase, putative			PF3D7_0513300	purine nucleoside phosphorylase (PNP)	purine metabolism	0	0	0	na	catalytic activity	nucleoside metabolic process	null
PBANKA_111330	deoxyribodipyrimidine photolyase, putative			PF3D7_0513600	deoxyribodipyrimidine photolyase (photoreactivating enzyme, DNA photolyase), putative	DNA replication	0	0	0	na	deoxyribodipyrimidine photo-lyase activity	DNA repair	null
PBANKA_111370	tubulin-tyrosine ligase, putative			PF3D7_0514000	tubulin-tyrosine ligase, putative (TTL)	microtubule	0	0	0	na	tubulin-tyrosine ligase activity	cellular protein modification process	null
PBANKA_111520	conserved Plasmodium protein, unknown function			PF3D7_0515600	conserved Plasmodium protein, unknown function	unknown function	0	0	0	na	null	null	null
PBANKA_111540	conserved Plasmodium protein, unknown function			PF3D7_0515800	BolA-like protein, putative	cell division	0	0	0	na	null	null	null
PBANKA_111610	conserved Plasmodium protein, unknown function			PF3D7_0616600	conserved Plasmodium protein, unknown function	unknown function	0	0	0	na	null	null	null
PBANKA_111740	conserved Plasmodium protein, unknown function			PF3D7_0618200	conserved Plasmodium protein, unknown function	unknown function	0	0	0	na	null	null	null
PBANKA_111750	ribosomal protein L27a, putative			PF3D7_0618300	60S ribosomal protein L27a, putative	ribosome	0	0	0	na	structural constituent of ribosome	translation	intracellular, ribosome
PBANKA_111860	cell division cycle protein 48 homologue, putative			PF3D7_0619400	cell division cycle protein 48 homologue, putative	cell division	0	0	0	na	ATP binding, nucleoside-triphosphatase activity	null	null
PBANKA_111920	conserved Plasmodium protein, unknown function	-3.28	0.75	PF3D7_0620000	conserved Plasmodium protein, unknown function	unknown function	0	0	0	na	null	null	null
PBANKA_111960	merozoite surface protein 10, putative (MSP10)			PF3D7_0620400	merozoite surface protein 10 (MSP10)	contaminant	0	0	0	na	null	null	null
PBANKA_111990	DnaJ protein, putative			PF3D7_0620700	DnaJ protein, putative	chaperone	0	0	0	na	heat shock protein binding	null	null
PBANKA_112100	conserved Plasmodium protein, unknown function			PF3D7_0622100	conserved Plasmodium protein, unknown function	unknown function	0	0	0	na	null	null	null
PBANKA_112200	coronin binding protein, putative			PF3D7_0623100	coronin binding protein, putative	gliding motility	0	0	0	na	null	null	null
PBANKA_112350	SNF2 helicase, putative (ISWI)			PF3D7_0624600	SNF2 helicase, putative (ISWI)	chromatin	0	0	0	na	ATP binding, DNA binding, helicase activity, protein binding, zinc ion binding	null	nucleus
PBANKA_112380	conserved Plasmodium protein, unknown function			PF3D7_0624900	conserved Plasmodium protein, unknown function	unknown function	0	0	0	na	null	null	null
PBANKA_112470	conserved Plasmodium protein, unknown function			PF3D7_0625900	conserved Plasmodium protein, unknown function	unknown function	0	0	0	na	null	null	null
PBANKA_112560	pyruvate kinase, putative			PF3D7_0626800	pyruvate kinase (PyrK)	glycolysis	0	0	0	na	magnesium ion binding, potassium ion binding, pyruvate kinase activity	glycolysis	null
PBANKA_112580	conserved Plasmodium protein, unknown function			PF3D7_0627000	conserved Plasmodium protein, unknown function	unknown function	0	0	0	na	null	null	null
PBANKA_112700	choline/ethanolaminephosphotransferase (CEPT)			PF3D7_0628300	choline/ethanolaminephosphotransferase, putative (CEPT)	phosphatidylcholine metabolism	0	0	0	KO not successful	phosphotransferase activity, for other substituted phosphate groups	phospholipid biosynthetic process	endoplasmic reticulum, membrane
PBANKA_112780	DnaJ protein, putative			PF3D7_0629200	DnaJ protein, putative	chaperone	0	0	0	na	heat shock protein binding, unfolded protein binding	protein folding	null
PBANKA_112890	conserved Plasmodium protein, unknown function			PF3D7_0630100	conserved Plasmodium protein, unknown function	unknown function	0	0	0	na	null	null	null

PBANKA_112910	DNA polymerase epsilon, putative			PF3D7_0630300	DNA polymerase epsilon, catalytic subunit a, putative	DNA repair	0	0	0	na	DNA binding, DNA-directed DNA polymerase activity, nucleotide binding	DNA replication	null
PBANKA_112960	conserved Plasmodium protein, unknown function			PF3D7_0630800	conserved Plasmodium protein, unknown function	unknown function	0	0	0	na	null	null	null
PBANKA_113050	proteasome subunit alpha type-7, putative			PF3D7_1353900	proteasome subunit, putative	proteasome	0	0	0	na	threonine-type endopeptidase activity	ubiquitin-dependent protein catabolic process	proteasome core complex
PBANKA_113150	conserved Plasmodium protein, unknown function			PF3D7_1354900	conserved Plasmodium protein, unknown function	unknown function	0	0	0	na	null	null	null
PBANKA_113160	minchromosome maintenance (MCM) complex subunit, putative (MCM6)			PF3D7_1355100	minchromosome maintenance (MCM) complex subunit (MCM6)	DNA replication	0	0	0	na	ATP binding, DNA binding	DNA replication, DNA-dependent DNA replication initiation	nucleus
PBANKA_113260	ubiquitin conjugating enzyme, putative			PF3D7_1356300	ubiquitin conjugating enzyme, putative	proteasome	0	0	0	na	small conjugating protein ligase activity	post-translational protein modification, regulation of protein metabolic process	null
PBANKA_113390	conserved Plasmodium protein, unknown function			PF3D7_1357600	conserved Plasmodium protein, unknown function	unknown function	0	0	0	na	null	null	null
PBANKA_113460	rhomboid protease ROM7, putative (ROM7)			PF3D7_1358300	rhomboid protease ROM7 (ROM7)	rhomboid	0	0	0	KO not successful	null	null	integral to membrane
PBANKA_113500	conserved Plasmodium protein, unknown function			PF3D7_1358700	conserved Plasmodium protein, unknown function	unknown function	0	0	0	na	null	null	null
PBANKA_113700	falcilysin, putative (FLN)			PF3D7_1360800	falcilysin (FLN)	protease	0	0	0	na	catalytic activity, metalloendopeptidase activity, zinc ion binding	proteolysis	apicoplast, food vacuole
PBANKA_113770	cytochrome c oxidase subunit 2, putative			PF3D7_1361700	cytochrome c oxidase subunit 2, putative	redoxmeth	0	0	0	na	copper ion binding, cytochrome-c oxidase activity	null	membrane
PBANKA_113790	proliferating cell nuclear antigen, putative (PCNA)			PF3D7_1361900	proliferating cell nuclear antigen (PCNA)	DNA replication	0	0	0	na	DNA binding, DNA polymerase processivity factor activity	regulation of DNA replication	PCNA complex
PBANKA_113820	RuvB-like helicase, putative (RUVB3)	-2.45	0.73	PF3D7_1362200	ATP-dependent DNA helicase, putative	DNA replication	0	0	0	na	ATP binding, DNA helicase activity, nucleoside-triphosphatase activity	null	nucleus
PBANKA_114060	DNA-directed RNA polymerase II, putative			PF3D7_1364800	DNA-directed RNA polymerase 2, putative	transcription	0	0	0	na	DNA binding, DNA-directed RNA polymerase activity	transcription, DNA-dependent	nucleus
PBANKA_114170	60S ribosomal protein L40/UBI, putative			PF3D7_1365900	60S ribosomal protein L40/UBI, putative	proteasome	0	0	0	na	structural constituent of ribosome	translation	intracellular, ribosome
PBANKA_114280	conserved Plasmodium protein, unknown function			PF3D7_1366900	conserved Plasmodium protein, unknown function	unknown function	0	0	0	na	null	null	null
PBANKA_114300	U1 small nuclear ribonucleoprotein, putative			PF3D7_1367100	U1 small nuclear ribonucleoprotein, putative	mRNA	0	0	0	na	nucleic acid binding	null	null
PBANKA_114590	conserved rodent malaria protein, unknown function					unknown function	0	0	0	na	null	null	null
PBANKA_120010	BIR protein					BIR protein	0	0	0	na	null	null	null
PBANKA_120040	BIR protein					BIR protein	0	0	0	na	null	null	null
PBANKA_120060	Plasmodium exported protein, unknown function					unknown function	0	0	0	KO not successful	null	null	null
PBANKA_120150	conserved Plasmodium protein, unknown function			PF3D7_1003100	conserved Plasmodium protein, unknown function	unknown function	0	0	0	na	null	null	null
PBANKA_120220	U5 small nuclear ribonuclear protein, putative			PF3D7_1003800	U5 small nuclear ribonuclear protein, putative	mRNA	0	0	0	na	GTP binding, GTPase activity	null	null
PBANKA_120240	60S ribosomal protein L13, putative			PF3D7_1004000	60S ribosomal protein L13, putative	ribosome	0	0	0	na	structural constituent of ribosome	translation	intracellular, large ribosomal subunit, ribosome
PBANKA_120270	RNA-binding protein, putative	-2.26	0.75	PF3D7_1004400	RNA binding protein, putative	mRNA	0	0	0	na	nucleic acid binding	null	null
PBANKA_120370	regulator of nonsense transcripts, putative			PF3D7_1005500	regulator of nonsense transcripts, putative	mRNA	0	0	0	na	ATP binding, DNA binding, helicase activity, zinc ion binding	nuclear-transcribed mRNA catabolic process, nonsense-mediated decay	cytoplasm
PBANKA_120420	conserved Plasmodium protein, unknown function			PF3D7_1006000	conserved Plasmodium protein, unknown function	unknown function	0	0	0	na	DNA binding, alkylbase DNA N-glycosylase activity	base-excision repair	null
PBANKA_120440	DNA/RNA-binding protein Alba 3, putative (ALBA3)			PF3D7_1006200	DNA/RNA-binding protein Alba 3 (ALBA3)	mRNA	0	0	0	na	DNA binding, RNA binding	null	nucleus
PBANKA_120480	thioredoxin, putative			PF3D7_1006600	thioredoxin, putative	redoxmeth	0	0	0	na	null	null	null
PBANKA_120500	RNA-binding protein, putative			PF3D7_1006800	RNA binding protein, putative	mRNA	0	0	0	na	nucleic acid binding	null	null
PBANKA_120540	rhoGAP GTPase, putative			PF3D7_1007200	rhoGAP GTPase, putative	gliding motility	0	0	0	na	null	signal transduction	intracellular

PBANKA_120660	26S proteasome regulatory subunit 4, putative			PF3D7_1008400	26S proteasome regulatory subunit 4, putative	proteasome	0	0	0	na	ATP binding, nucleoside-triphosphatase activity	protein catabolic process	cytoplasm, nucleus
PBANKA_120690	tubulin beta chain, putative			PF3D7_1008700	tubulin beta chain	gliding motility	0	0	0	na	GTP binding, GTPase activity, structural molecule activity	microtubule-based movement, protein polymerization	microtubule, protein complex
PBANKA_120700	small subunit rRNA processing protein, putative			PF3D7_1008800	small subunit rRNA processing protein, putative	RNA	0	0	0	na	null	null	null
PBANKA_120880	conserved Plasmodium protein, unknown function			PF3D7_1010400	conserved Plasmodium protein, unknown function	kinase	0	0	0	na	null	null	null
PBANKA_120890	conserved Plasmodium protein, unknown function					proteasome	0	0	0	na	null	null	null
PBANKA_120900	eukaryotic translation initiation factor 2, beta, putative			PF3D7_1010600	eukaryotic translation initiation factor 2 beta subunit, putative	translation	0	0	0	na	translation initiation factor activity	translational initiation	null
PBANKA_120990	conserved Plasmodium protein, unknown function			PF3D7_1011500	conserved Plasmodium membrane protein, unknown function	unknown function	0	0	0	na	null	null	null
PBANKA_121070	ubiquinol-cytochrome c reductase complex subunit, putative			PF3D7_1012300	ubiquinol-cytochrome c reductase complex subunit, putative	redoxmeth	0	0	0	na	ubiquinol-cytochrome-c reductase activity	mitochondrial electron transport, ubiquinol to cytochrome c	null
PBANKA_121090	phosphoglucomutase, putative			PF3D7_1012500	phosphoglucomutase, putative	glycolysis	0	0	0	na	intramolecular transferase activity, phosphotransferases	carbohydrate metabolic process	null
PBANKA_121260	male gamete fusion factor HAP2 (HAP2)			PF3D7_1014200	male gamete fusion factor HAP2, putative (HAP2)	sexual development	0	0	0	different in fertilization, ookinete, oocyst, sporozoite and liver stage	null	null	null
PBANKA_121270	conserved Plasmodium protein, unknown function			PF3D7_1014300	conserved Plasmodium protein, unknown function	unknown function	0	0	0	not different from wild type	null	null	null
PBANKA_121300	transcriptional activator ADA2, putative			PF3D7_1014600	transcriptional coactivator ADA2 (ADA2)	transcription	0	0	0	na	DNA binding, zinc ion binding	null	null
PBANKA_121350	conserved Plasmodium protein, unknown function			PF3D7_1015100	conserved protein, unknown function	unknown function	0	0	0	na	null	null	null
PBANKA_121360	cysteinyl-tRNA synthetase, putative			PF3D7_1015200.1/2	cysteinyl-tRNA synthetase, putative	translation	0	0	0	na	ATP binding, cysteine-tRNA ligase activity	cysteinyl-tRNA aminoacylation, translation	cytoplasm
PBANKA_121400	heat shock protein 60, putative			PF3D7_1015600	heat shock protein 60 (HSP60)	chaperone	0	0	0	na	ATP binding, protein binding	cellular protein metabolic process	null
PBANKA_121430	enolase, putative (ENO)			PF3D7_1015900	enolase (ENO)	glycolysis	0	0	0	na	phosphopyruvate hydratase activity	glycolysis	phosphopyruvate hydratase complex
PBANKA_121470	conserved Plasmodium protein, unknown function			PF3D7_0323800	conserved Plasmodium protein, unknown function	unknown function	0	0	0	na	null	null	null
PBANKA_121530	conserved Plasmodium protein, unknown function			PF3D7_0323200	conserved Plasmodium protein, unknown function	unknown function	0	0	0	na	null	null	null
PBANKA_121650	peptidyl-prolyl cis-trans isomerase, putative (CYP19A)			PF3D7_0322000	peptidyl-prolyl cis-trans isomerase (CYP19A)	chaperone	0	0	0	na	peptidyl-prolyl cis-trans isomerase activity	null	null
PBANKA_121760	histone H2A variant, putative (H2A.Z)			PF3D7_0320900	histone H2A variant, putative (H2A.Z)	chromatin	0	0	0	na	DNA binding	nucleosome assembly	nucleosome, nucleus
PBANKA_121770	ATP-dependent RNA Helicase (DOZI)			PF3D7_0320800	ATP-dependent RNA helicase, putative (DOZI)	mRNA	0	0	0	different in fertilization and ookinete	ATP binding, ATP-dependent helicase activity, helicase activity, nucleic acid binding	null	null
PBANKA_121830	CPW-WPC family protein, putative	-2.75	0.75	PF3D7_0320200	CPW-WPC family protein	adhesin	0	0	0	na	null	null	null
PBANKA_121840	SNARE protein, putative			PF3D7_0320100	SNARE protein (SEC22)	trafficking	0	0	0	na	null	vesicle-mediated transport	integral to membrane
PBANKA_121850	conserved Plasmodium protein, unknown function			PF3D7_0320000	conserved Plasmodium protein, unknown function	unknown function	0	0	0	na	null	null	null
PBANKA_121860	conserved Plasmodium protein, unknown function			PF3D7_0319900	conserved Plasmodium protein, unknown function	unknown function	0	0	0	na	null	null	null
PBANKA_121890	elongation factor 1 (EF-1), putative					translation	0	0	0	na	translation elongation factor activity	translational elongation	eukaryotic translation elongation factor 1 complex
PBANKA_121900	heat shock protein 90, putative			PF3D7_0708500	heat shock protein 86 family protein	chaperone	0	0	0	na	null	null	null
PBANKA_121920	Cg8 protein, putative			PF3D7_0708700	Cg8 protein	redoxmeth	0	0	0	na	null	null	null
PBANKA_122120	60S ribosomal protein L34a, putative			PF3D7_0710600	60S ribosomal protein L34a, putative	ribosome	0	0	0	na	structural constituent of ribosome	translation	intracellular, ribosome
PBANKA_122130	conserved Plasmodium protein, unknown function			PF3D7_0710700	conserved Plasmodium protein, unknown function	unknown function	0	0	0	na	null	null	null
PBANKA_122240	ubiquitin regulatory protein, putative			PF3D7_0808300	ubiquitin regulatory protein, putative	proteasome	0	0	0	na	null	null	null

PBANKA_122280	tyrosyl-tRNA synthetase, putative			PF3D7_0807900	tyrosyl-tRNA synthetase, putative	translation	0	0	0	na	ATP binding, tyrosine-tRNA ligase activity	translation, tyrosyl-tRNA aminoacylation	cytoplasm
PBANKA_122420	DnaJ protein, putative			PF3D7_0806500	DnaJ protein, putative	chaperone	0	0	0	na	heat shock protein binding, unfolded protein binding	protein folding	null
PBANKA_122540	conserved Plasmodium protein, unknown function			PF3D7_0805200	conserved Plasmodium protein, unknown function	unknown function	0	0	0	na	null	null	null
PBANKA_122660	tubulin gamma chain, putative			PF3D7_0803700	tubulin gamma chain (g-tub)	gliding motility	0	0	0	na	GTP binding, GTPase activity	microtubule-based process, protein polymerization	microtubule, protein complex
PBANKA_122680	DNA repair protein rad54, putative	-1.65	0.7	PF3D7_0803400	DNA repair protein rad54, putative	DNA repair	0	0	0	na	ATP binding, DNA binding, helicase activity	null	null
PBANKA_122720	peptidyl-prolyl cis-trans isomerase, putative (CYP81)			PF3D7_0803000	peptidyl-prolyl cis-trans isomerase (CYP81)	chaperone	0	0	0	na	peptidyl-prolyl cis-trans isomerase activity	null	null
PBANKA_122740	serine/threonine protein phosphatase 2B catalytic subunit A, putative (CNA)			PF3D7_0802800	serine/threonine protein phosphatase, putative	phosphatase	0	0	0	na	hydrolase activity	null	null
PBANKA_122780	conserved Plasmodium protein, unknown function			PF3D7_0802400	conserved Plasmodium protein, unknown function	unknown function	0	0	0	na	null	null	null
PBANKA_122830	conserved Plasmodium protein, unknown function			PF3D7_0801900	conserved Plasmodium protein, unknown function	redoxmeth	0	0	0	na	electron carrier activity, oxidoreductase activity	null	null
PBANKA_122920	60S ribosomal protein L19, putative			PF3D7_0614500	60S ribosomal protein L19, putative	ribosome	0	0	0	na	structural constituent of ribosome	translation	intracellular, ribosome
PBANKA_123005	ribonuclease, putative, fragment			PF3D7_0615400	ribonuclease, putative	mRNA	0	0	0	na	RNA binding, ribonuclease activity	null	null
PBANKA_123100	40S ribosomal protein S14, putative			PF3D7_0516200	40S ribosomal protein S14, putative	ribosome	0	0	0	na	structural constituent of ribosome	translation	intracellular, ribosome
PBANKA_123160	transcription factor with AP2 domain(s), putative (ApiAP2)			PF3D7_0516800	transcription factor with AP2 domain(s) (ApiAP2)	transcription	0	0	0	na	sequence-specific DNA binding transcription factor activity	regulation of transcription, DNA-dependent	null
PBANKA_123170	60S ribosomal protein L8, putative			PF3D7_0516900	60S ribosomal protein L8, putative	ribosome	0	0	0	na	structural constituent of ribosome	translation	intracellular, ribosome
PBANKA_123180	60S ribosomal protein L12, putative			PF3D7_0517000	60S ribosomal protein L12, putative	ribosome	0	0	0	na	structural constituent of ribosome	translation	intracellular, ribosome
PBANKA_123350	mRNA-binding protein PUF1 (PUF1)			PF3D7_0518700	mRNA-binding protein PUF1 (PUF1)	mRNA	0	0	0	different in fertilization, ookinete, oocyst, sporozoite and liver stage	RNA binding	null	null
PBANKA_123420	40S ribosomal protein S24, putative			PF3D7_0519400	40S ribosomal protein S24, putative	ribosome	0	0	0	na	structural constituent of ribosome	translation	intracellular, ribosome
PBANKA_123450	conserved Plasmodium protein, unknown function			PF3D7_0519700	conserved Plasmodium protein, unknown function	unknown function	0	0	0	na	null	null	null
PBANKA_123480	40S ribosomal protein S9, putative			PF3D7_0520000	40S ribosomal protein S9, putative	ribosome	0	0	0	na	rRNA binding, structural constituent of ribosome	translation	intracellular, small ribosomal subunit
PBANKA_123510	U6 snRNA-associated sm-like protein lsm2, putative (LSM2)			PF3D7_0520300	U6 snRNA-associated sm-like protein lsm2, putative (LSM2)	mRNA	0	0	0	na	null	null	null
PBANKA_123770	zinc finger protein, putative, fragment						0	0	0	na	null	null	null
PBANKA_123880	karyopherin beta, putative			PF3D7_0524000	karyopherin beta (KASbeta)	nuclear pore	0	0	0	na	binding	null	null
PBANKA_123920	cytosolic preribosomal GTP-binding protein, putative			PF3D7_0524400	cytosolic preribosomal GTP-binding protein, putative	ribosome assembly	0	0	0	na	GTP binding	null	intracellular
PBANKA_123940	organelle ribosomal protein L7/L12 precursor, putative			PF3D7_0524600	organelle ribosomal protein L7/L12 precursor, putative	ribosome	0	0	0	na	structural constituent of ribosome	translation	intracellular, ribosome
PBANKA_123980	zinc finger protein, putative			PF3D7_0525000	zinc finger protein, putative	RNA binding, RNA processing	0	0	0	na	nucleic acid binding, zinc ion binding	null	null
PBANKA_124180	minchromosome maintenance (MCM) complex subunit, putative (MCM3)			PF3D7_0527000	minchromosome maintenance (MCM) complex subunit, putative (MCM3)	DNA replication	0	0	0	na	ATP binding, DNA binding, nucleoside-triphosphatase activity	DNA replication, DNA-dependent DNA replication initiation	nucleus
PBANKA_124190	ubiquitin-conjugating enzyme E2 N, putative (UBC13)			PF3D7_0527100	ubiquitin-conjugating enzyme E2 N, putative (UBC13)	proteasome	0	0	0	na	small conjugating protein ligase activity	post-translational protein modification, regulation of protein metabolic process	null
PBANKA_124220	conserved Plasmodium protein, unknown function			PF3D7_0527400	conserved Plasmodium protein, unknown function	unknown function	0	0	0	na	null	null	null
PBANKA_124230	58 kDa phosphoprotein,heat shock-related protein (HRP)			PF3D7_0527500	hsp70 interacting protein, putative	trafficking	0	0	0	na	null	chaperone cofactor-dependent protein refolding	null
PBANKA_124310	F-actin capping protein, alpha subunit, putative			PF3D7_0528500	f-actin capping protein alpha subunit, putative	gliding motility	0	0	0	na	actin binding	actin cytoskeleton organization	F-actin capping protein complex
PBANKA_124320	conserved Plasmodium protein, unknown function			PF3D7_0528600	conserved Plasmodium protein, unknown function	unknown function	0	0	0	na	null	null	null

PBANKA_124590	Plasmodium exported protein, unknown function					unknown function	0	0	0	na	null	null	null
PBANKA_124640	BIR protein					BIR protein	0	0	0	na	null	null	null
PBANKA_124690	BIR protein, pseudogene					BIR protein	0	0	0	na	null	null	null
PBANKA_130050	tubulin, putative			PF3D7_1475700	tubulin, putative	chromatin	0	0	0	na	GTP binding, GTPase activity, structural molecule activity	microtubule-based movement, protein polymerization	microtubule, protein complex
PBANKA_130070	LCCL domain-containing protein (CCp1)			PF3D7_1475500	LCCL domain-containing protein (CCp1)	adhesin	0	0	0	different in oocyst and sporozoite; (tagged) also in fertilization and ookinete	receptor binding	cell adhesion, signal transduction	null
PBANKA_130130	trailer hitch homolog, putative (CITH)			PF3D7_1474900	trailer hitch homolog, putative (CITH)	mRNA	0	0	0	different in fertilization and ookinete	null	null	null
PBANKA_130140	proteosome subunit alpha type 1, putative			PF3D7_1474800	proteosome subunit alpha type 1, putative	proteasome	0	0	0	na	threonine-type endopeptidase activity	ubiquitin-dependent protein catabolic process	proteasome core complex
PBANKA_130160	conserved Plasmodium protein, unknown function			PF3D7_1474600	conserved Plasmodium membrane protein, unknown function	unknown function	0	0	0	na	null	null	null
PBANKA_130260	small subunit DNA primase, putative			PF3D7_1438700	small subunit DNA primase	DNA replication	0	0	0	na	DNA primase activity	DNA replication, synthesis of RNA primer	null
PBANKA_130280	thioredoxin peroxidase 1 (TPx1)			PF3D7_1438900	thioredoxin peroxidase 1 (Trx-Px1)	redoxmeth	0	0	0	different in sporozoite and liver stage	antioxidant activity, oxidoreductase activity	cell redox homeostasis	null
PBANKA_130330	ubiquinol-cytochrome c reductase, iron-sulfur subunit, putative			PF3D7_1439400	ubiquinol-cytochrome c reductase iron-sulfur subunit, putative	redoxmeth	0	0	0	na	2 iron, 2 sulfur cluster binding, electron carrier activity, ubiquinol-cytochrome-c reductase activity	oxidation-reduction process	membrane
PBANKA_130350	conserved Plasmodium protein, unknown function			PF3D7_1439600	conserved Plasmodium protein, unknown function	unknown function	0	0	0	na	null	null	mitochondrion
PBANKA_130390	conserved Plasmodium protein, unknown function	-3.01	0.75	PF3D7_1440000	conserved Plasmodium protein, unknown function	unknown function	0	0	0	na	RNA-directed RNA polymerase activity	viral genome replication	null
PBANKA_130510	60S ribosomal protein L10a, putative			PF3D7_1441200	60S ribosomal protein L1, putative	ribosome	0	0	0	na	RNA binding	RNA processing	null
PBANKA_130590	ADP-ribosylation-like factor, putative			PF3D7_1442000	ADP-ribosylation factor, putative	trafficking	0	0	0	na	GTP binding	intracellular protein transport, small GTPase mediated signal transduction	intracellular
PBANKA_130600	conserved Plasmodium protein, unknown function			PF3D7_1442100	conserved Plasmodium protein, unknown function	DNA repair	0	0	0	na	null	null	null
PBANKA_130620	tRNA binding protein, putative			PF3D7_1442300	tRNA binding protein, putative	translation	0	0	0	na	tRNA binding	null	null
PBANKA_130770	zinc finger protein, putative			PF3D7_1443800	zinc finger protein, putative	mRNA	0	0	0	na	nucleic acid binding, protein binding, zinc ion binding	null	null
PBANKA_130810	calmodulin-like protein			PF3D7_1444200	calmodulin-like protein	kinase	0	0	0	na	null	null	null
PBANKA_130920	serine/threonine kinase-1, putative			PF3D7_1445400	protein serine/threonine kinase-1 (CLK1)	kinase	0	0	0	KO not successful	ATP binding, protein serine/threonine kinase activity, protein tyrosine kinase activity	protein phosphorylation	null
PBANKA_130930	conserved Plasmodium protein, unknown function			PF3D7_1445500	conserved Plasmodium protein, unknown function	unknown function	0	0	0	na	null	null	null
PBANKA_131040	centrin, putative			PF3D7_1446600	centrin-2 (CEN2)	chromatin	0	0	0	na	calcium ion binding	null	null
PBANKA_131080	40S ribosomal protein S2, putative			PF3D7_1447000	40S ribosomal protein S2, putative	ribosome	0	0	0	na	RNA binding, structural constituent of ribosome	translation	intracellular, ribosome, small ribosomal subunit
PBANKA_131090	conserved Plasmodium protein, unknown function			PF3D7_1447100	conserved Plasmodium protein, unknown function	unknown function	0	0	0	na	null	null	null
PBANKA_131440	conserved Plasmodium protein, unknown function			PF3D7_1450700	conserved Plasmodium protein, unknown function	unknown function	0	0	0	na	null	null	null
PBANKA_131480	elongation factor 2, putative			PF3D7_1451100	elongation factor 2	translation	0	0	0	na	GTP binding, GTPase activity	null	null
PBANKA_131490	conserved Plasmodium protein, unknown function	-2.62	0.75	PF3D7_1451200	conserved Plasmodium protein, unknown function	unknown function	0	0	0	na	null	null	null
PBANKA_131550	sortilin, putative			PF3D7_1451800	sortilin, putative	large tethering	0	0	0	na	null	null	integral to membrane
PBANKA_131740	co-chaperone p23, putative			PF3D7_1453700	co-chaperone p23 (P23)	chaperone	0	0	0	na	null	null	null
PBANKA_131890	conserved Plasmodium protein, unknown function			PF3D7_1455200.1/2	conserved Plasmodium protein, unknown function	unknown function	0	0	0	na	methyltransferase activity	metabolic process	null
PBANKA_132010	conserved Plasmodium protein, unknown function (UIS25)			PF3D7_1456400	conserved Plasmodium protein, unknown function	unknown function	0	0	0	na	null	null	null

PBANKA_132020	conserved Plasmodium protein, unknown function			PF3D7_1456500	conserved Plasmodium protein, unknown function	unknown function	0	0	0	na	null	null	null
PBANKA_132070	signal peptide peptidase, putative (SPP)			PF3D7_1457000	signal peptide peptidase (SPP)	post-translational modification	0	0	0	na	aspartic-type endopeptidase activity	null	endoplasmic reticulum, integral to membrane
PBANKA_132150	conserved Plasmodium protein, unknown function			PF3D7_1457800	conserved Plasmodium protein, unknown function	unknown function	0	0	0	na	null	null	null
PBANKA_132400	ribosomal protein L29, putative			PF3D7_1460300	ribosomal protein L29, putative	ribosome	0	0	0	na	structural constituent of ribosome	translation	intracellular, ribosome
PBANKA_132510	conserved Plasmodium protein, unknown function			PF3D7_1461400	conserved Plasmodium protein, unknown function	kinase	0	0	0	na	null	null	null
PBANKA_132520	conserved Plasmodium protein, unknown function			PF3D7_1461500	conserved Plasmodium protein, unknown function	meiosis	0	0	0	na	null	null	null
PBANKA_132610	conserved Plasmodium protein, unknown function			PF3D7_1462500	conserved Plasmodium protein, unknown function	unknown function	0	0	0	na	null	null	null
PBANKA_132640	glyceraldehyde-3-phosphate dehydrogenase, putative			PF3D7_1462800	glyceraldehyde-3-phosphate dehydrogenase (GAPDH)	glycolysis	0	0	0	na	NAD binding, glyceraldehyde-3-phosphate dehydrogenase (NAD+) (phosphorylating) activity	glucose metabolic process	null
PBANKA_132730	conserved Plasmodium protein, unknown function					unknown function	0	0	0	na	null	null	null
PBANKA_132830	ATP-dependent protease la, putative			PF3D7_1464900	ATP-dependent protease la, putative	protease	0	0	0	na	ATP binding, metalloendopeptidase activity, nucleoside-triphosphatase activity, zinc ion binding	protein catabolic process, proteolysis	integral to membrane, membrane
PBANKA_132840	conserved Plasmodium protein, unknown function			PF3D7_1465000	conserved Plasmodium protein, unknown function	unknown function	0	0	0	na	null	null	null
PBANKA_132900	conserved Plasmodium protein, unknown function			PF3D7_1465600	conserved Plasmodium protein, unknown function	unknown function	0	0	0	na	null	null	null
PBANKA_132930	40S ribosomal protein S3, putative			PF3D7_1465900	40S ribosomal protein S3, putative	ribosome	0	0	0	na	RNA binding, structural constituent of ribosome	translation	intracellular, ribosome, small ribosomal subunit
PBANKA_132980	transcription factor with AP2 domain(s) (AP2-SP)			PF3D7_1466400	transcription factor with AP2 domain(s) (ApiAP2)	transcription	0	0	0	different in oocyst and sporozoite	sequence-specific DNA binding transcription factor activity	regulation of transcription, DNA-dependent	null
PBANKA_133140	conserved Plasmodium protein, unknown function			PF3D7_1468100	conserved Plasmodium protein, unknown function	galactose metabolism	0	0	0	KO not successful	ATP binding	null	null
PBANKA_133210	conserved Plasmodium protein, unknown function			PF3D7_1468900	conserved Plasmodium protein, unknown function	unknown function	0	0	0	na	null	null	null
PBANKA_133300	conserved Plasmodium protein, unknown function			PF3D7_1469800	conserved Plasmodium protein, unknown function	unknown function	0	0	0	na	null	null	null
PBANKA_133320	conserved Plasmodium protein, unknown function			PF3D7_1470000	ATG12	autophagy	0	0	0	na	null	autophagic vacuole assembly	cytoplasm
PBANKA_133480	conserved Plasmodium protein, unknown function	-2.16	0.72	PF3D7_1471600	conserved Plasmodium protein, unknown function	unknown function	0	0	0	na	null	null	null
PBANKA_133490	conserved Plasmodium protein, unknown function	-4.61	0.75			unknown function	0	0	0	na	null	null	null
PBANKA_133520	pre-mRNA-splicing factor, putative			PF3D7_1472000	pre-mRNA-splicing factor ISY1 homolog, putative	mRNA	0	0	0	na	null	null	null
PBANKA_133570	conserved Plasmodium protein, unknown function			PF3D7_1472500	conserved Plasmodium protein, unknown function	unknown function	0	0	0	na	null	null	null
PBANKA_133660	conserved Plasmodium protein, unknown function			PF3D7_1473400	conserved Plasmodium protein, unknown function	unknown function	0	0	0	na	null	null	null
PBANKA_133690	conserved Plasmodium protein, unknown function					unknown function	0	0	0	na	null	null	null
PBANKA_133710	splicing factor, putative			PF3D7_1321700	splicing factor, putative	mRNA	0	0	0	na	RNA binding	mRNA processing	nucleus
PBANKA_133840	vacuolar ATP synthase subunit g, putative			PF3D7_1323200	vacuolar ATP synthase subunit g, putative	vacuolar ATP synthase	0	0	0	KO not successful	hydrolase activity, acting on acid anhydrides, catalyzing transmembrane movement of substances	proton transport	vacuolar proton-transporting V-type ATPase complex
PBANKA_133940	microfibril-associated protein homologue, putative			PF3D7_1324200	micro-fibrillar-associated protein, putative	gliding motility	0	0	0	na	null	null	extracellular region
PBANKA_134010	L-lactate dehydrogenase (LDH)			PF3D7_1324900	L-lactate dehydrogenase (LDH)	glycolysis	0	0	0	na	binding, oxidoreductase activity, acting on the CH-OH group of donors, NAD or NADP as acceptor	glycolysis	null
PBANKA_134020	U6 snRNA-associated Sm-like protein LSm6, putative (LSM6)	-2.49	0.75	PF3D7_1325000	U6 snRNA-associated Sm-like protein LSm6, putative (LSM6)	mRNA	0	0	0	na	null	null	null
PBANKA_134040	oxidoreductase, putative			PF3D7_1325200	oxidoreductase, putative	glycolysis	0	0	0	na	binding, oxidoreductase activity, acting on the CH-OH group of donors, NAD or NADP as acceptor	glycolysis	null
PBANKA_134150	RNA binding protein, putative			PF3D7_1326300	RNA binding protein, putative	mRNA	0	0	0	na	nucleic acid binding	null	null

PBANKA_134320	conserved Plasmodium protein, unknown function	PF3D7_1328000	conserved Plasmodium protein, unknown function	unknown function	0	0	0	na	null	null	null
PBANKA_134560	elongation factor Tu, putative	PF3D7_1330600	elongation factor Tu, putative	translation	0	0	0	na	GTP binding, GTPase activity, translation elongation factor activity	translational elongation	intracellular
PBANKA_134610	conserved Plasmodium protein, unknown function	PF3D7_1331200	conserved Plasmodium protein, unknown function	unknown function	0	0	0	na	null	null	null
PBANKA_134840	histone h3-like centromeric protein cse4, putative (CENPA)	PF3D7_1333700	histone H3 variant, putative (CenH3)	chromatin	0	0	0	na	DNA binding	nucleosome assembly	nucleosome, nucleus
PBANKA_135000	conserved Plasmodium protein, unknown function	PF3D7_1336100	conserved Plasmodium protein, unknown function	unknown function	0	0	0	na	null	null	null
PBANKA_135030	glucose-induced degradation protein 8, putative (GID8)	PF3D7_1336400	glucose-induced degradation protein 8, putative (GID8)	protein-protein interaction	0	0	0	na	null	null	null
PBANKA_135050	conserved Plasmodium protein, unknown function	PF3D7_1336700	conserved Plasmodium protein, unknown function	unknown function	0	0	0	na	calcium ion binding	null	null
PBANKA_135130	conserved Plasmodium protein, unknown function			unknown function	0	0	0	na	null	null	null
PBANKA_135210	conserved Plasmodium protein, unknown function	PF3D7_1338400	conserved Plasmodium protein, unknown function	unknown function	0	0	0	na	null	null	null
PBANKA_135430	sodium-dependent phosphate transporter, putative (PiT)	PF3D7_1340900	sodium-dependent phosphate transporter (PiT)	acidocalcisome, transporter	0	0	0	na	inorganic phosphate transmembrane transporter activity	phosphate ion transport	membrane
PBANKA_135440	60S ribosomal protein L18, putative	PF3D7_1341200	60S ribosomal protein L18, putative	ribosome	0	0	0	na	structural constituent of ribosome	translation	intracellular, ribosome
PBANKA_135450	60S ribosomal protein L18-2, putative	PF3D7_1341300	60S ribosomal protein L18-2, putative	ribosome	0	0	0	na	structural constituent of ribosome	translation	intracellular, ribosome
PBANKA_135500	vacuolar ATP synthase subunit d, putative	PF3D7_1341900	vacuolar ATP synthase subunit d, putative	vacuolar ATP synthase	0	0	0	na	proton-transporting ATPase activity, rotational mechanism	ATP synthesis coupled proton transport	proton-transporting two-sector ATPase complex, catalytic domain
PBANKA_135510	40S ribosomal protein S6, putative	PF3D7_1342000	40S ribosomal protein S6, putative	ribosome	0	0	0	na	structural constituent of ribosome	translation	intracellular, ribosome
PBANKA_135550	casein kinase II beta chain, putative	PF3D7_1342400	casein kinase II beta chain (CK2beta2)	kinase	0	0	0	na	protein kinase regulator activity	null	protein kinase CK2 complex
PBANKA_135580	transcription activator, putative	PF3D7_1342700	transcription activator, putative	transcription	0	0	0	na	DNA binding, DNA-directed RNA polymerase activity	transcription, DNA-dependent	null
PBANKA_135630	conserved Plasmodium protein, unknown function	PF3D7_1343300	conserved Plasmodium protein, unknown function	unknown function	0	0	0	na	null	null	null
PBANKA_135670	kelch protein k13, putative	PF3D7_1343700	kelch protein, putative	galactose metabolism	0	0	0	na	protein binding, voltage-gated potassium channel activity	potassium ion transport	membrane, voltage-gated potassium channel complex
PBANKA_135780	conserved Plasmodium protein, unknown function	PF3D7_1344900	conserved Plasmodium protein, unknown function	unknown function	0	0	0	na	null	null	null
PBANKA_135830	conserved Plasmodium protein, unknown function	PF3D7_1345400	conserved Plasmodium protein, unknown function	unknown function	0	0	0	na	null	null	null
PBANKA_135980	conserved Plasmodium protein, unknown function	PF3D7_1346900	conserved Plasmodium protein, unknown function	unknown function	0	0	0	na	null	null	null
PBANKA_136030	DNA/RNA-binding protein Alba 4, putative (ALBA4)	PF3D7_1347500	DNA/RNA-binding protein Alba 4 (ALBA4)	mRNA	0	0	0	na	DNA binding, RNA binding	null	cytoplasm, nucleus
PBANKA_136120	conserved Plasmodium protein, unknown function	PF3D7_1348400	conserved Plasmodium membrane protein, unknown function	unknown function	0	0	0	na	null	null	null
PBANKA_136150	conserved Plasmodium protein, unknown function	PF3D7_1348700	conserved Plasmodium protein, unknown function	phototransduction	0	0	0	na	null	null	null
PBANKA_136200	glutamate - tRNA ligase, putative	PF3D7_1349200	glutamate--tRNA ligase, putative	translation	0	0	0	na	ATP binding, glutamate-tRNA ligase activity	glutamyl-tRNA aminoacylation, translation	cytoplasm
PBANKA_136420	60S ribosomal protein L17, putative	PF3D7_1351400	60S ribosomal protein L17, putative	ribosome	0	0	0	na	structural constituent of ribosome	translation	intracellular, large ribosomal subunit, ribosome
PBANKA_140010	BIR protein			BIR protein	0	0	0	na	null	null	null
PBANKA_140060	cytoadherence linked asexual protein, putative				0	0	0	KO not successful	null	null	null
PBANKA_140070	Plasmodium exported protein, unknown function			unknown function	0	0	0	na	null	null	null
PBANKA_140150	conserved Plasmodium protein, unknown function	PF3D7_1303000	conserved Plasmodium protein, unknown function	unknown function	0	0	0	na	null	null	null
PBANKA_140260	DNA ligase I, putative	PF3D7_1304100	DNA ligase I (LigI)	DNA repair	0	0	0	KO not successful	ATP binding, DNA binding, DNA ligase (ATP) activity	DNA recombination, DNA repair, DNA replication	null

PBANKA_140490	26s protease regulatory subunit 10b, putative (RPT4)	PF3D7_1306400	26S proteasome regulatory subunit, putative	proteasome	0	0	0	na	ATP binding, nucleoside-triphosphatase activity	protein catabolic process	cytoplasm, nucleus
PBANKA_140610	DNA-directed RNA polymerase alpha chain, putative	PF3D7_1307600	DNA-directed RNA polymerase alpha chain, putative	transcription	0	0	0	na	DNA binding, DNA-directed RNA polymerase activity, protein dimerization activity	transcription, DNA-dependent	null
PBANKA_140700	conserved Plasmodium protein, unknown function	PF3D7_1308500	conserved Plasmodium protein, unknown function	glycolysis	0	0	0	na	null	null	null
PBANKA_140740	mRNA-decapping enzyme 2, putative (DCP2)	PF3D7_1308900	mRNA-decapping enzyme 2, putative (DCP2)	mRNA	0	0	0	na	hydrolase activity	null	null
PBANKA_140760	60S ribosomal protein L24, putative	PF3D7_1309100	60S ribosomal protein L24, putative	ribosome	0	0	0	na	structural constituent of ribosome	translation	intracellular, ribosome
PBANKA_140780	U4/U6 small nuclear ribonucleoprotein PRP3, putative (PRPF3)	PF3D7_1309300	U4/U6 small nuclear ribonucleoprotein PRP3, putative (PRPF3)	mRNA	0	0	0	na	null	null	null
PBANKA_140920	rna-binding protein, putative	PF3D7_1310700	conserved Plasmodium protein, unknown function	unknown function	0	0	0	na	nucleic acid binding	null	null
PBANKA_140960	conserved Plasmodium protein, unknown function	PF3D7_1311100	conserved Plasmodium protein, unknown function	unknown function	0	0	0	na	null	null	null
PBANKA_141000	26s protease regulatory subunit 7, putative (RPT1)	PF3D7_1311500	26S proteasome regulatory subunit 7, putative	proteasome	0	0	0	na	ATP binding, nucleoside-triphosphatase activity	protein catabolic process	cytoplasm, nucleus
PBANKA_141120	conserved Plasmodium protein, unknown function	PF3D7_1312800	conserved Plasmodium protein, unknown function	unknown function	0	0	0	na	null	null	null
PBANKA_141140	ubiquitin-like protein nedd8 homologue, putative	PF3D7_1313000	ubiquitin-like protein nedd8 homologue, putative (Nedd8)	proteasome	0	0	0	na	null	null	null
PBANKA_141170	peptidyl-prolyl cis-trans isomerase, putative	PF3D7_1313300	peptidyl-prolyl cis-trans isomerase, putative	chaperone	0	0	0	na	null	protein folding	null
PBANKA_141320	conserved Plasmodium protein, unknown function	PF3D7_1314700	conserved Plasmodium protein, unknown function	unknown function	0	0	0	na	null	null	null
PBANKA_141400	conserved Plasmodium protein, unknown function	PF3D7_1315500	conserved Plasmodium protein, unknown function	unknown function	0	0	0	na	null	null	null
PBANKA_141470	ADP-ribosylation factor, putative	PF3D7_1316200	ADP-ribosylation factor, putative	trafficking	0	0	0	KO not successful	GTP binding	small GTPase mediated signal transduction	intracellular
PBANKA_141560	minchromosome maintenance (MCM) complex subunit, putative (MCM4)	PF3D7_1317100	minchromosome maintenance (MCM) complex subunit (MCM4)	DNA replication	0	0	0	na	ATP binding, DNA binding	DNA replication, DNA-dependent DNA replication initiation	nucleus
PBANKA_141630	40S ribosomal protein S15/S19, putative	PF3D7_1317800	40S ribosomal protein S15/S19, putative	ribosome	0	0	0	na	structural constituent of ribosome	translation	intracellular, ribosome, small ribosomal subunit
PBANKA_141690	chromosome segregation protein, putative	PF3D7_1318400	chromosome segregation protein, putative	chromatin	0	0	0	na	ATP binding, protein binding	chromosome organization	chromosome
PBANKA_141730	translocation protein sec63, putative	PF3D7_1318800	secretory complex protein 63 (SEC63)	trafficking	0	0	0	na	heat shock protein binding, unfolded protein binding	protein folding	null
PBANKA_141840	ATP-dependent Clp protease adaptor protein, putative	PF3D7_1320100	ATP-dependent Clp protease adaptor protein ClpS, putative	chaperone	0	0	0	na	null	protein catabolic process	null
PBANKA_142050	conserved Plasmodium protein, unknown function	PF3D7_0713900	conserved Plasmodium protein, unknown function	unknown function	0	0	0	na	null	null	null
PBANKA_142070	conserved Plasmodium protein, unknown function	PF3D7_0714100	conserved Plasmodium protein, unknown function	unknown function	0	0	0	na	null	null	null
PBANKA_142100	calmodulin, putative	PF3D7_0714400	calmodulin, putative	kinase	0	0	0	na	null	null	null
PBANKA_142350	60S ribosomal protein L13-2, putative	PF3D7_0814000	60S ribosomal protein L13-2, putative	ribosome	0	0	0	na	structural constituent of ribosome	translation	intracellular, ribosome
PBANKA_142360	40S ribosomal protein S16, putative	PF3D7_0813900	40S ribosomal protein S16, putative	ribosome	0	0	0	na	structural constituent of ribosome	translation	intracellular, ribosome
PBANKA_142430	conserved Plasmodium protein, unknown function	PF3D7_0813300	conserved Plasmodium protein, unknown function	unknown function	0	0	0	na	null	null	null
PBANKA_142490	ubiquitin-conjugating enzyme, putative	PF3D7_0812600	ubiquitin conjugating enzyme, putative	proteasome	0	0	0	na	small conjugating protein ligase activity	post-translational protein modification, regulation of protein metabolic process	null
PBANKA_142510	karyopherin alpha, putative (KARalpha)	PF3D7_0812400	karyopherin alpha (KARalpha)	nuclear pore	0	0	0	na	binding, protein transporter activity	protein import into nucleus	cytoplasm, nuclear pore, nucleus
PBANKA_142620	CCR4-associated factor 1, putative (CAF1)	PF3D7_0811300	CCR4-associated factor 1 (CAF1)	mRNA	0	0	0	KO not successful	nucleic acid binding	null	nucleus
PBANKA_142690	RNA helicase, putative	PF3D7_0810600	RNA helicase, putative	mRNA	0	0	0	na	ATP binding, ATP-dependent helicase activity, helicase activity, nucleic acid binding	null	null
PBANKA_142750	acyl CoA binding protein, putative	PF3D7_0810000	acyl-CoA binding protein, putative	lipid metabolism	0	0	0	na	fatty-acyl-CoA binding	null	null

PBANKA_142870	eukaryotic translation initiation factor 3 subunit 10, putative			PF3D7_1212700	eukaryotic translation initiation factor 3 subunit 10, putative	translation	0	0	0	na	null	null	null
PBANKA_142890	bromodomain protein, putative			PF3D7_1212900	bromodomain protein, putative	chromatin	0	0	0	na	null	null	nucleus
PBANKA_142940	dynein light chain 1, putative			PF3D7_1213600	dynein light chain 1, putative	dynein/kinesin	0	0	0	na	microtubule motor activity	microtubule-based process	microtubule associated complex
PBANKA_143120	conserved Plasmodium protein, unknown function			PF3D7_1215500	conserved Plasmodium protein, unknown function	unknown function	0	0	0	na	cAMP-dependent protein kinase regulator activity	signal transduction	null
PBANKA_143140	conserved Plasmodium protein, unknown function			PF3D7_1215700	conserved Plasmodium protein, unknown function	unknown function	0	0	0	na	null	null	null
PBANKA_143200	signal recognition particle 19 kD protein, putative			PF3D7_1216300	signal recognition particle SRP19 (SRP19)	post-translational modification	0	0	0	na	7S RNA binding	SRP-dependent cotranslational protein targeting to membrane	signal recognition particle
PBANKA_143220	male development gene 1 (MDV1)			PF3D7_1216500	male development gene 1 (MDV1)	egress, sexual development	0	0	0	different in fertilization, ookinete, oocyst and sporozoite	null	null	null
PBANKA_143240	perforin like protein 2 (PPLP2)			PF3D7_1216700	perforin like protein 2 (PPLP2)	perforin	0	0	0	different in fertilization, ookinete and oocyst	null	null	null
PBANKA_143300	conserved Plasmodium protein, unknown function			PF3D7_1217400	conserved Plasmodium protein, unknown function	unknown function	0	0	0	na	null	null	null
PBANKA_143330	conserved Plasmodium protein, unknown function			PF3D7_1217700	conserved Plasmodium protein, unknown function	unknown function	0	0	0	na	null	null	null
PBANKA_143400	phosphate translocator, putative			PF3D7_1218400	triose or hexose phosphate/phosphate translocator, putative	transporter	0	0	0	na	null	null	null
PBANKA_143440	secreted ookinete protein, putative (PSOP17)			PF3D7_1218800	conserved Plasmodium protein, unknown function (PSOP17)	adhesin	0	0	0	KO not successful	null	null	null
PBANKA_143480	aminophospholipid-transporting P-ATPase, putative			PF3D7_1219600	aminophospholipid-transporting P-ATPase (ATPase2)	transporter	0	0	0	KO not successful	ATP binding, ATPase activity, coupled to transmembrane movement of ions, phosphorylative mechanism, magnesium ion binding, phospholipid-translocating ATPase activity	ATP biosynthetic process, phospholipid transport	integral to membrane, membrane
PBANKA_143520	conserved Plasmodium protein, unknown function			PF3D7_1220000	conserved Plasmodium protein, unknown function	unknown function	0	0	0	na	null	null	null
PBANKA_143570	ribosome biogenesis protein TSR3, putative (TSR3)			PF3D7_1220500	ribosome biogenesis protein TSR3, putative (TSR3)	ribosome	0	0	0	na	null	null	null
PBANKA_143610	heterochromatin protein 1, putative (HP1)			PF3D7_1220900	heterochromatin protein 1 (HP1)	chromatin	0	0	0	na	chromatin binding	chromatin assembly or disassembly	chromatin, nucleus
PBANKA_143650	conserved Plasmodium protein, unknown function	-1.92	0.74	PF3D7_1221300	conserved Plasmodium protein, unknown function	unknown function	0	0	0	na	null	null	null
PBANKA_143710	conserved Plasmodium protein, unknown function			PF3D7_1221900	conserved Plasmodium membrane protein, unknown function	unknown function	0	0	0	na	null	null	null
PBANKA_143730	endoplasmin homolog precursor, putative			PF3D7_1222300	endoplasmin homolog precursor, putative		0	0	0	na	ATP binding, unfolded protein binding	protein folding, response to stress	null
PBANKA_143740	ATP-binding protein, putative			PF3D7_1222500	ATP-binding protein, putative		0	0	0	na	null	null	null
PBANKA_143800	CAMP-dependent protein kinase regulatory subunit, putative (PKAr)			PF3D7_1223100	cAMP-dependent protein kinase regulatory subunit (PKAr)	kinase	0	0	0	na	cAMP-dependent protein kinase regulator activity	regulation of protein phosphorylation	cAMP-dependent protein kinase complex
PBANKA_143920	polyadenylate-binding protein, putative (PABP)			PF3D7_1224300	polyadenylate-binding protein, putative (PABP)	mRNA	0	0	0	na	RNA binding	null	null
PBANKA_143980	splicing factor 3b subunit, putative (SF3B14)			PF3D7_1224900	splicing factor 3b subunit, putative (SF3B14)	mRNA	0	0	0	na	nucleic acid binding	null	null
PBANKA_144110	hydrolase, putative			PF3D7_1226300	cof-like hydrolase, had-superfamily, subfamily iib	hydrolase	0	0	0	na	hydrolase activity	metabolic process	null
PBANKA_144180	conserved Plasmodium protein, unknown function			PF3D7_1227000	conserved Plasmodium protein, unknown function	unknown function	0	0	0	na	null	null	null
PBANKA_144300	NIMA related kinase 1, putative (NEK1)			PF3D7_1228300	NIMA related kinase 1 (NEK1)	kinase	0	0	0	KO not successful	ATP binding, protein serine/threonine kinase activity, protein tyrosine kinase activity	protein phosphorylation	null
PBANKA_144540	transport protein Sec13, putative			PF3D7_1230700	protein transport protein Sec13, putative	trafficking	0	0	0	na	null	null	null
PBANKA_144570	conserved Plasmodium protein, unknown function	-1.74	0.7	PF3D7_1231000	conserved Plasmodium protein, unknown function	unknown function	0	0	0	na	null	null	null
PBANKA_144590	conserved Plasmodium protein, unknown function			PF3D7_1231200	conserved Plasmodium protein, unknown function	unknown function	0	0	0	na	null	null	null
PBANKA_144900	conserved Plasmodium protein, unknown function			PF3D7_1234400	conserved Plasmodium protein, unknown function	unknown function	0	0	0	KO not successful	aspartic-type endopeptidase activity	proteolysis	null
PBANKA_144950	conserved Plasmodium protein, unknown function			PF3D7_1234900	conserved Plasmodium protein, unknown function	unknown function	0	0	0	na	null	null	null

PBANKA_145020	serine hydroxymethyltransferase, putative (SHMT)			PF3D7_1235600	serine hydroxymethyltransferase (SHMT)	pyrimidine metabolism	0	0	0	na	glycine hydroxymethyltransferase activity	L-serine metabolic process, glycine metabolic process	null
PBANKA_145070	clustered-asparagine-rich protein, putative			PF3D7_1236100	clustered-asparagine-rich protein		0	0	0	na	nucleic acid binding, protein binding	null	null
PBANKA_145150	conserved Plasmodium protein, unknown function			PF3D7_1236900	conserved Plasmodium protein, unknown function	unknown function	0	0	0	na	null	null	null
PBANKA_145240	ubiquitin-like modifier hub1, putative (HUB1)			PF3D7_1237800	ubiquitin-like protein, putative	proteasome	0	0	0	na	null	null	null
PBANKA_145250	conserved Plasmodium protein, unknown function			PF3D7_1237900	conserved Plasmodium protein, unknown function	RNA	0	0	0	na	null	null	null
PBANKA_145270	conserved Plasmodium protein, unknown function			PF3D7_1238200	conserved Plasmodium protein, unknown function	unknown function	0	0	0	na	null	null	null
PBANKA_145340	protein kinase 2 (PK2)	-2.9	0.75	PF3D7_1238900	protein kinase 2 (PK2)	kinase	0	0	0	KO not successful	ATP binding, protein serine/threonine kinase activity, protein tyrosine kinase activity	protein phosphorylation	null
PBANKA_145480	RNA binding protein, putative			PF3D7_1241400	RNA binding protein, putative	mRNA	0	0	0	na	nucleic acid binding	null	null
PBANKA_145510	replication factor C subunit 4, putative			PF3D7_1241700	replication factor C subunit 4, putative	DNA replication	0	0	0	na	ATP binding, DNA clamp loader activity	DNA replication	DNA replication factor C complex
PBANKA_145570	conserved Plasmodium protein, unknown function			PF3D7_1242300	conserved Plasmodium membrane protein, unknown function	unknown function	0	0	0	na	null	null	null
PBANKA_145680	endosome sorting protein (SNF7 homologue), putative			PF3D7_1243500	endosome sorting protein (SNF7 homologue), putative	large tethering	0	0	0	na	null	protein transport	null
PBANKA_145690	translation initiation factor SUI1, putative			PF3D7_1243600	translation initiation factor SUI1, putative	translation	0	0	0	na	translation initiation factor activity	translation, translational initiation	null
PBANKA_145720	double C2-like domain-containing protein, putative (DOC2)			PF3D7_1243900	double C2-like domain-containing protein (DOC2)	trafficking, transporter	0	0	0	na	null	entry into host	null
PBANKA_145780	ADP-ribosylation factor, putative			PF3D7_1244600	ADP-ribosylation factor GTPase-activating protein, putative	trafficking	0	0	0	na	ARF GTPase activator activity, zinc ion binding	regulation of ARF GTPase activity	null
PBANKA_145790	conserved Plasmodium protein, unknown function			PF3D7_1244700	conserved Plasmodium protein, unknown function	unknown function	0	0	0	na	null	null	null
PBANKA_145850	nedd8-conjugating enzyme ubc12, putative (UBC12)			PF3D7_1245300	ubiquitin conjugating enzyme E2, putative	proteasome	0	0	0	na	ATP binding, small conjugating protein ligase activity	post-translational protein modification, regulation of protein metabolic process	null
PBANKA_145930	actin I			PF3D7_1246200	actin I (ACT1)	gliding motility	0	0	0	na	protein binding	null	cytoplasm, nucleus
PBANKA_146060	conserved Plasmodium protein, unknown function			PF3D7_1247700	conserved Plasmodium protein, unknown function	unknown function	0	0	0	na	uroporphyrinogen-III synthase activity	tetrapyrrole biosynthetic process	null
PBANKA_146120	conserved Plasmodium protein, unknown function			PF3D7_1248300	conserved Plasmodium membrane protein, unknown function	unknown function	0	0	0	na	null	null	null
PBANKA_146140	conserved Plasmodium protein, unknown function			PF3D7_1248500	conserved protein, unknown function	apoptosis	0	0	0	na	null	null	null
PBANKA_146180	tat-binding protein homolog, putative			PF3D7_1248900	tat-binding protein homolog	proteasome	0	0	0	na	ATP binding, nucleoside-triphosphatase activity	protein catabolic process	cytoplasm, nucleus
PBANKA_146300	osmiophilic body protein (G377)			PF3D7_1250100	osmiophilic body protein (Pfg377)	adhesin	0	0	0	not different from wild type	null	null	osmiophilic body
