# Supplementary material for: Genome-wide RIP-Chip analysis of translational repressor-bound mRNAs in the Plasmodium gametocyte
Source: Genome Biol. 2014 Nov 3;15(11):493. doi: 10.1186/s13059-014-0493-0 (PMC4234863; doi:10.1186/s13059-014-0493-0)
Supplement: Additional file 2: Figure S1. — Presents the strategy employed for the generation of PBANKA_072090 null mutants_a and _b. Only data pertaining to mutant _a are shown. Data for mutant _b are available upon request. [file 13059_2014_493_MOESM2_ESM.pdf]

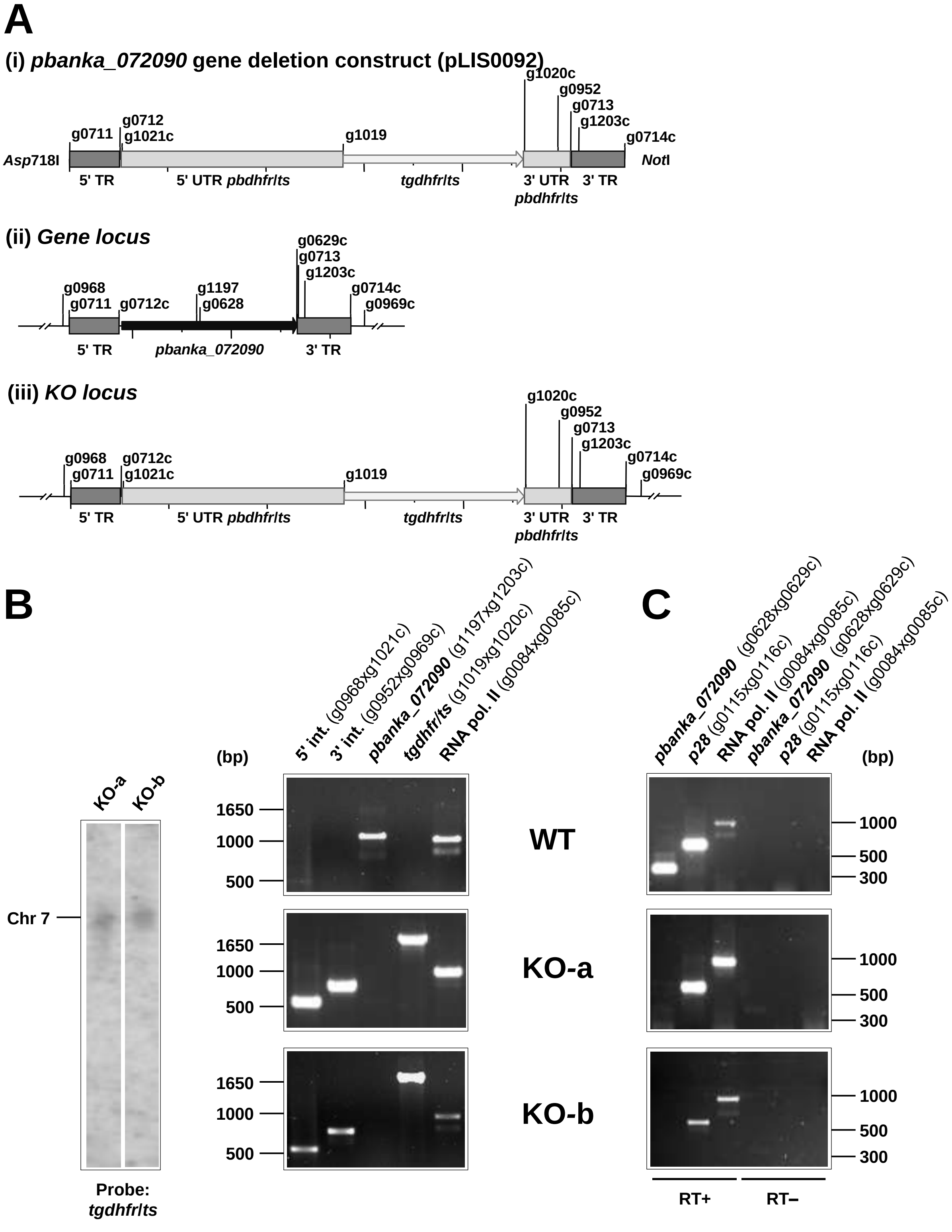

Figure S1 – Generation and genotyping of pbanka_072090 knock-out (KO) parasite lines. (A) *pbanka_072090* gene deletion construct pLIS0092 (i) was obtained by cloning 5' and 3' targeting regions (TR) upstream and downstream of the *Toxoplasma gondii dhfr/ts* selectable marker cassette, respectively. The *tgdhfr/ts* gene is under the control of *P. berghei dhfr/ts* 5' and 3' UTRs. The construct was integrated into the *pbanka_072090 locus* (ii) of Fluo-frmg WT line by double homologous recombination, resulting in the complete deletion of the ORF in the null mutant (iii). **(B)** Correct deletion of *pbanka_072090* was shown by Southern analysis of separated chromosomes (left) and diagnostic PCR analyses (right). Hybridisation of separated chromosomes with a probe against the *tgdhfr/ts* selectable marker cassette recognised integrated pLIS0092 into chromosome 7. PCR analyses confirm 5' and 3' integration (int.) of pLIS0092, absence of the ORF and presence of the *tgdhfr/ts* gene. **(C)** Absence of *pbanka_072090* mRNA was confirmed by RT-PCR. *p28* and RNA polymerase II serve as control genes.
