# Supplementary material for: Genome-wide RIP-Chip analysis of translational repressor-bound mRNAs in the Plasmodium gametocyte
Source: Genome Biol. 2014 Nov 3;15(11):493. doi: 10.1186/s13059-014-0493-0 (PMC4234863; doi:10.1186/s13059-014-0493-0)
Supplement: Additional file 3: Table S2. — Reports the GO terms enriched in Plasmodium genes found associated with DOZI and/or CITH. [file 13059_2014_493_MOESM3_ESM.pdf]

Table S2: GO terms enriched in *Plasmodium* genes found associated with DOZI and / or CITH. Enrichement analysis was performed using the GOstats Bioconductor package (Falcon S & Gentleman R, 2007). GO id = Gene ontology term identification number. Term = annotation. Observed = number of genes found annotaed with that particular term. Phyper = p value given by the hypergeometric test. Categories = BP for biological process, CC for cellular componenet and MF for molecular function.

GO Id	Term	Observed	Phyper	Categories
GO:0006310	DNA recombination	2	0.00948199	BP
GO:0051603	proteolysis	4	0.03189045	BP
GO:0006396	RNA processing	11	0.00889958	BP
GO:0015031	protein transport	6	0.0440648	BP
GO:0005739	mitochondrion	19	1.49E-03	CC
GO:0020039	pellicle	2	1.08E-02	CC
GO:0016020	membrane	20	2.43E-02	CC
GO:0030529	ribonucleoprotein complex	19	4.85E-02	CC
GO:0015301	anion-anion antiporter activity	2	0.00703586	MF
GO:0016853	isomerase activity	5	0.01726434	MF
GO:0008134	transcription factors binding	2	0.01994221	MF
