# Supplementary material for: Genome-wide RIP-Chip analysis of translational repressor-bound mRNAs in the Plasmodium gametocyte
Source: Genome Biol. 2014 Nov 3;15(11):493. doi: 10.1186/s13059-014-0493-0 (PMC4234863; doi:10.1186/s13059-014-0493-0)
Supplement: Additional file 4: Table S3. — Reports the gene enrichment analysis of P. berghei genes found associated with DOZI and/or CITH. [file 13059_2014_493_MOESM4_ESM.pdf]

Table S3: Gene enrichement analysis of *P. berghei* genes found associated with DOZI and / or CITH. A hypergeometric test was used to identify manually grouped genes. Term = manual grouping of genes. Observed = number of genes found annotated with that particular term. p value = p value given by the hypergeometric test. FDR corrected = p values corrected for multiple testing.

**Term**	**Observed**	**p value**	**FDR corrected**
adhesins	22	0.0024102	0.028922434
gliding motility	26	8.281E-06	0.000505112
trafficking	31	4.041E-05	0.001848853
transporters	26	5.44E-08	3.62024E-06
chaperone	17	7.2E-12	7.52945E-10
kinase	15	1.68E-13	2.04942E-11
RNA binding	16	2.225E-09	2.03562E-07
RNA metabolisn	20	0.0003892	0.010550801
splicing	21	1.101E-13	1.61198E-11
microtubule	10	3.36E-05	0.001639588
phosphatase	7	0.0204386	0.1010882
proteasome	16	6.708E-20	1.22761E-17
redox	17	0.0022518	0.027937801
