# Supplementary material for: Genome-wide RIP-Chip analysis of translational repressor-bound mRNAs in the Plasmodium gametocyte
Source: Genome Biol. 2014 Nov 3;15(11):493. doi: 10.1186/s13059-014-0493-0 (PMC4234863; doi:10.1186/s13059-014-0493-0)
Supplement: Additional file 5: Figure S2. — Presents a schematic of the gliding motility apparatus in Plasmodium parasites and lists the D/C-bound transcripts involved in this process. [file 13059_2014_493_MOESM5_ESM.pdf]

A

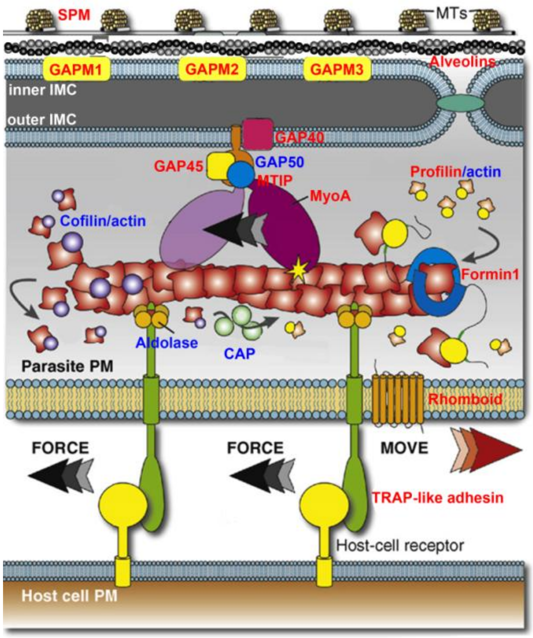

B

PBANKA 040270 | ALV2
PBANKA_052390 | GAPM2
PBANKA 070710 | membrane skeletal IMC1-related
PBANKA 081070 | SPM1
PBANKA_081900 | GAP50
PBANKA 083300 | profilin, putative (PFN)
PBANKA 090710 | IMC1b
PBANKA_103540 | GAPM3
PBANKA 110310 | ADF1
PBANKA_111530 | GAP40
PBANKA 112040 | Pf77 protein (ALV7)
PBANKA 120200 | ALV5
PBANKA_120940 | conserved, unknown function
PBANKA 124060 | membrane skeletal IMC1-related
PBANKA 133890 | GAPM1
PBANKA_134980 | TRAP
PBANKA 135490 | conserved Plasmodium, unknown function
PBANKA 135570 | myosin A (MyoA)
PBANKA_135850 | conserved Plasmodium, unknown function
PBANKA 136440 | ALV6
PBANKA 143660 | ALV3
PBANKA_143760 | GAP45
PBANKA 144500 | SPM2
PBANKA 145950 | MTIP

Figure S2. A model of the gliding motility complex and selected, associated IMC proteins. (A) All transcripts found associated with Dozi/Cith are highlighted in red. MTs = microtubules, IMC = inner membrane complex, PM = plasma membrane. Adapted from Baum et al., 2008, Trends Parasitol. 24(12): 557–563, with kind permission of Cell Press. **(B)** All transcripts enriched in D/C-bound fractions listed with gene ID and annotations (taken from www.genedb.org) .
