# Supplementary material for: Genome-wide RIP-Chip analysis of translational repressor-bound mRNAs in the Plasmodium gametocyte
Source: Genome Biol. 2014 Nov 3;15(11):493. doi: 10.1186/s13059-014-0493-0 (PMC4234863; doi:10.1186/s13059-014-0493-0)
Supplement: Additional file 6: Table S4. — List the primers used in GFP-tagged parasite lines and KO lines generation, as well as RT-PCRs. [file 13059_2014_493_MOESM6_ESM.pdf]

Table S4. Primers used in GFP-tagged and KO parasite lines generation and RT-PCRs. Nucleotide stretches in capital letter correspond to the complementary sequence to the respective gene.

Primer name	sequence	gene
g0084	aaagaattcTGATGGTTTACAATCACC	PBANKA_080700
g0085	aaagcggccgctTTCTTCCTGCATCTCCTC	PBANKA_080700
g0115	TTCGATATCATGAATTTTAAATACAG	p28 | PBANKA_051490
g0116	tccgcggccgcGCATTACTATCACGTAAATAAC	p28 | PBANKA_051490
g0333	aaatgatcaTTCTTTAATTGTAATTTCATTG	api2-o | PBANKA_090590
g0334	aaatgatcACTAGTTTAAATAAAGATG	api2-o | PBANKA_090590
g0408	GTATGTTGCATCACCTTC	*gfp*
g0546	TAATTGTGTCGCTTCAAATG	dozi | PBANKA_121770
g0548	TAATTCTTTTATCATAGCAG	dozi | PBANKA_121770
g0549	GAAAAAAGCAAAGATGTATTATCTG	cith | PBANKA_130130
g0550	ATAGGCTGGGTATCTGTTAAATG	cith | PBANKA_130130
g0583	aaagaattcTTACTTTTGGCAAAAGC	PBANKA_010770
g0584	aaaggatccTTTTTTCACATTCAATTC	PBANKA_010770
g0626	aaagaattcCTTTTAGTTTCATTTAATG	PBANKA_082120
g0627	aaagcggccgcAATAGAATAACTCTTCATTATC	PBANKA_082120
g0628	aaagaattcCATATATTAGAGTATTG	PBANKA_072090
g0629	aaagcggccgcAATTTGCCTTTTGTGCATC	PBANKA_072090
g0630	aaagaattcAAAATTTTAGATGTGTTAG	PBANKA_010770
g0637	aaacaattgAATACTAATGTTTATGAC	PBANKA_133470
g0638	aaagcggccgcATCTATAATGATATACTC	PBANKA_133470
g0639	aaagaattcCAATTATTTTCATTGTC	PBANKA_111410
g0640	aaagcggccgcAAATAATCAATTTTGTTAAT	PBANKA_111410
g0647	aaagaattcATGAATACTTATTACAG	p25 | PBANKA_051500
g0648	aaagcggccgcAAATGATATTTGAAAATATTAG	p25 | PBANKA_051500
g0711	aaaggtaccTTGTTATTCTTTGCAAG	PBANKA_072090
g0712	aaaaagcttTGAATAATTAAAAAAAAGC	PBANKA_072090
g0713	aaagaattcTTATTTGATGATCAACC	PBANKA_072090

g0714	aaagcggccgcAATATATATAGTTATTTCC	PBANKA_072090
g0952	GATTCATAAATAGTTGGACTTG	PBANKA_072091
g0968	TACATTGAAGTGTTGGTATG	5' UTR PBANKA_072090
g0969	TGCATGCACATATATGTCAC	3' UTR PBANKA_072091
g1019	ATGCATAAACCGGTGTGTC	tgdhfr/ts
g1020	AGCTTCTGTATTTCCGC	tgdhfr/ts
g1021	ATTGTTGACCTGCAGGCATG	PBANKA_072090
g1197	GCCTTATGGAATTAGTGC	PBANKA_072090
g1203	GTATTAATGCATGACTTG	PBANKA_072090
g1267	AATTAGACTTAATAGATG	PBANKA_133470
g1268	AATTACTTTTGCTATATG	PBANKA_111410
g1269	ATATAAACCATCAATATG	PBANKA_082120
